# Assessing the sustainability of compliance with surgical site infection prophylaxis after discontinuation of mandatory active reporting: study protocol

**DOI:** 10.1186/s43058-022-00288-0

**Published:** 2022-04-25

**Authors:** Westyn Branch-Elliman, A. Rani Elwy, Rebecca L. Lamkin, Marlena Shin, Ryann L. Engle, Kathryn Colborn, Jessica Rove, Jacquelyn Pendergast, Kierstin Hederstedt, Mary Hawn, Hillary J. Mull

**Affiliations:** 1 Center for Healthcare Organization and Implementation Research, VA Boston Healthcare System, Boston, USA; 2 Department of Medicine, Infectious Disease Section, VA Boston Healthcare System, Boston, USA; 3 Harvard Medical School, Boston, USA; 4 Department of Medicine, Section of Infectious Diseases, VA Boston Healthcare System, 1400 VFW Parkway, West Roxbury, MB 02132 USA; 5 Center for Healthcare Organization and Implementation Research, VA Bedford Healthcare System, Bedford, USA; 6 Department of Psychiatry and Human Behavior, Warren Alpert Medical School, Brown University, Providence, USA; 7 Eastern Colorado VA Healthcare System, Aurora, USA; 8 Department of Surgery, University of Colorado Anschutz Medical Campus, Aurora, USA; 9 Department of Surgery, Stanford University School of Medicine, Stanford, USA; 10 Department of Surgery, Boston University School of Medicine, Boston, USA

**Keywords:** Dynamic sustainability framework, Sustainability, Informatics, Antimicrobial prophylaxis, Surgical care improvement project, Policy discontinuation, Diffusion of innovation, Scale up, Spread, ERIC (Expert Recommendations for Implementation Change)

## Abstract

**Background:**

Surgical site infections are common. Risk can be reduced substantially with appropriate preoperative antimicrobial administration. In 2005, the VA implemented the Surgical Care Improvement Project (SCIP) in the setting of high rates of non-compliance with antimicrobial prophylaxis guidelines. SCIP included public reporting of evidenced-based antimicrobial guideline compliance metrics in high-risk surgeries. SCIP was highly successful and led to high rates of adoption of preoperative antimicrobials and early discontinuation of postoperative antimicrobials (>95%). The program was retired in 2015, as the manual measurement and reporting process was costly with limited expected additional benefit. To our knowledge, no studies have assessed whether the gains achieved by SCIP were sustained since active support for the program was discontinued. Furthermore, there has been no investigation of the spread of antimicrobial prophylaxis guideline adoption beyond the limited set of procedures that were included in the program.

**Methods:**

Using a mixed methods sequential exploratory approach, this study will (1) quantitatively measure compliance with SCIP metrics over time and across all procedures in the five major surgical specialties targeted by SCIP and (2) collect qualitative data from stakeholders to identify strategies that were effective for sustaining compliance. Diffusion of Innovation Theory will guide assessment of whether improvements achieved spread to procedures not included under the umbrella of the program. Electronic algorithms to measure SCIP antimicrobial use will be adapted from previously developed methodology. These highly novel data mining algorithms leverage the rich VA electronic health record and capture structured and text data and represent a substantial technological advancement over resource-intensive manual chart review or incomplete electronic surveillance based on pharmacy data. An interrupted time series analysis will be used to assess whether SCIP compliance was sustained following program discontinuation. Generalized linear models will be used to assess whether compliance with appropriate prophylaxis increased in all SCIP targeted and non-targeted procedures by specialty over the duration the program’s active reporting. The Dynamic Sustainability Framework will guide the qualitative methods to assess intervention, provider, facility, specialty, and contextual factors associated with sustainability over time. Barriers and facilitators to sustainability will be mapped to implementation strategies and the study will yield an implementation playbook to guide future sustainment efforts.

**Relevance:**

Sustainability of practice change has been described as one of the most important, but least studied areas of clinical medicine. Learning how practices spread is also a critically important area of investigation. This study will use novel informatics strategies to evaluate factors associated with sustainability following removal of active policy surveillance and advance our understanding about these important, yet understudied, areas.

**Supplementary Information:**

The online version contains supplementary material available at 10.1186/s43058-022-00288-0.

Contributions to the literature

- Sustainability is an important, yet understudied area of implementation science and clinical medicine. Factors that support long-term sustainability following discontinuation of active programmatic support and surveillance are poorly characterized.
- This theory-driven study uses a mixed methods sequential exploratory approach and advanced informatics guided by the Dynamic Sustainability Framework and Theory of Diffusion of Innovations to identify factors associated with long-term practice change and spread of evidence-based practices.
- This study will provide insight into factors that drive sustainability and diffusion of innovation, and can be used to design future late-stage implementation interventions to promote long-term practice improvement. Barriers and facilitators to sustainability will be mapped to implementation strategies to identify best practices for supporting guideline compliance after active support for a program is discontinued.

## Background

Surgical site infections (SSIs) are one of the most common types of healthcare-associated infections, accounting for substantial morbidity and mortality. Pre-incision antimicrobial prophylaxis, administered within 1 h prior to incision, is highly effective for reducing SSI. Postoperative antimicrobials given after skin closure do not reduce SSI and increase severe adverse events, including acute kidney injuries and *Clostrideriodies difficile* infections [1–3]. Beyond the direct impact on the patient, excess antimicrobial use contributes to the burden of antimicrobial resistance, a critical healthcare threat and a major target of VA and non-VA initiatives [4].

In 2005, the VA implemented the Surgical Care Improvement Project (SCIP) to increase compliance with a bundle of SSI prevention and other quality improvement measures. SCIP was a Joint Commission initiative, which included a set of publicly reported evidenced-based antimicrobial guideline compliance metrics primarily targeting high-risk surgeries in five specialties, such as cardiac bypasses and orthopedic total joint replacements [5]. Public reporting of SCIP metrics required resource-intense manual review by a trained reviewer as part of the VA’s External Peer Review Program (EPRP) to assess compliance with the antimicrobial administration metrics. Following implementation of active reporting, VA compliance with guideline-concordant preoperative antimicrobial use (SCIP INF-1) and prompt discontinuation of antimicrobials postoperatively (SCIP INF-3) exceeded 95% [6–8]. After this high level of compliance was achieved, SCIP was retired in 2015, as the measurement and reporting process was felt to be costly with limited additional expected benefit [9, 10]. Since the program’s retirement, no studies have examined if the practice changes achieved through the active SCIP program were sustained, or if improvements spread beyond the originally targeted surgeries to procedures not included under the umbrella of the original program.

Sustainability is defined as “the extent to which an evidence-based intervention can deliver its intended benefits over an extended period of time after external support from the donor agency is terminated” [11, 12]. Sustainability of practice change has been called “one of the most significant translational research problems of our time” [13]. However, despite the critical importance of the topic, in 2004 Greenhalgh et al. noted a “near absence” of studies that aim to answer questions about factors driving high and low levels of sustainability [14]. In the past 17 years, there has continued to be a dearth of research providing insights into this important area [15]. The proposed study aims to address this important scientific gap by examining whether guideline concordant pre- and postoperative antimicrobial use adopted during SCIP were *sustained*, using the Dynamic Sustainability Framework [11] as a guide for the investigations. VA longitudinal databases, advanced informatics approaches, and qualitative data will be used to address the study aims. Assessment of program spread to uncovered specialties will be guided by the Diffusion of Innovations theory [16].

### Theoretical basis and frameworks

**Fig. 1:**
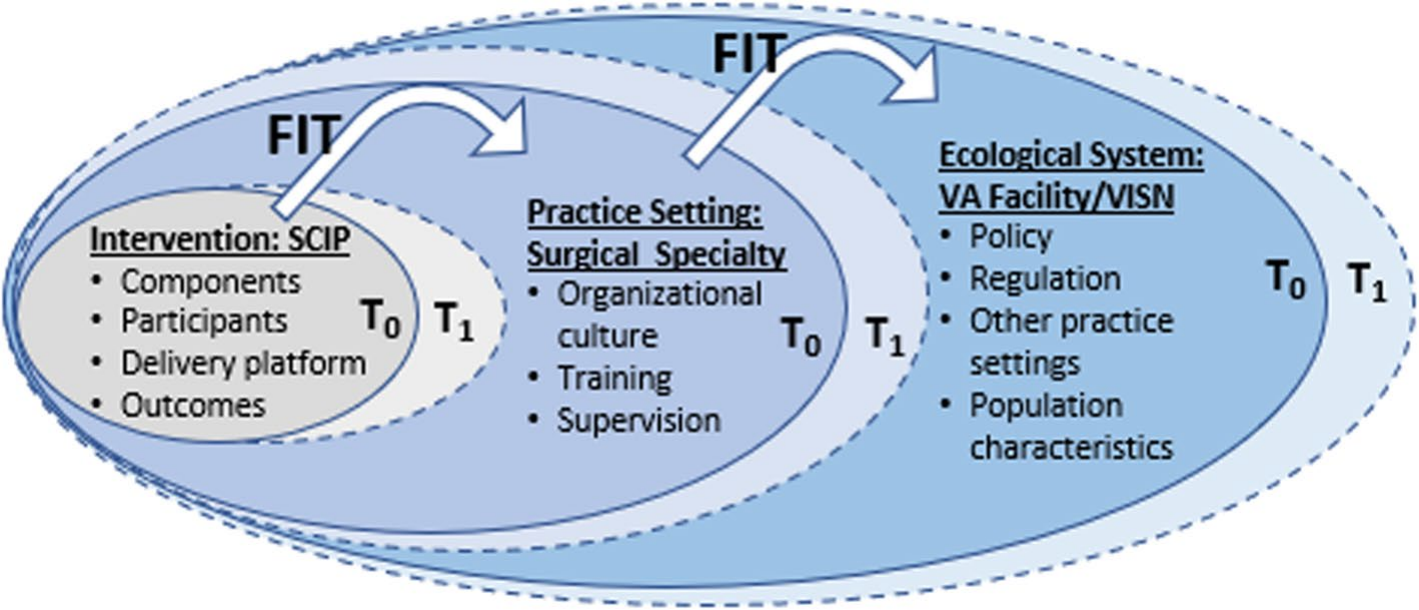
Dynamic Sustainability Framework (DSF), adapted to evaluate the sustainability of the Surgical Care Improvement Program

The research in this study is guided by Chambers et al. 2013 Dynamic Sustainability Framework (DSF; see Fig. 1 for DSF adapted to evaluate late-stage implementation outcomes for SCIP). The DSF emphasizes that *sustainability* is an ongoing aspect of implementation that requires continual adaptations and support. It highlights that successful sustainment of an intervention, such as evidence-based antimicrobial use practices, requires that the characteristics of the intervention be “consistently tracked, using valid, reliable, and relevant measures” and expects that the system will change and evolve overtime. Further, the framework underscores the importance of “ongoing assessment and quality improvement efforts” to “improve sustainment” and “identify opportunities for intervention improvement.” The DSF also highlights the importance of informatics advances to improve and enhance ongoing quality monitoring and improving efforts as part of a larger “learning healthcare system” model [11, 17].

**Fig. 2:**
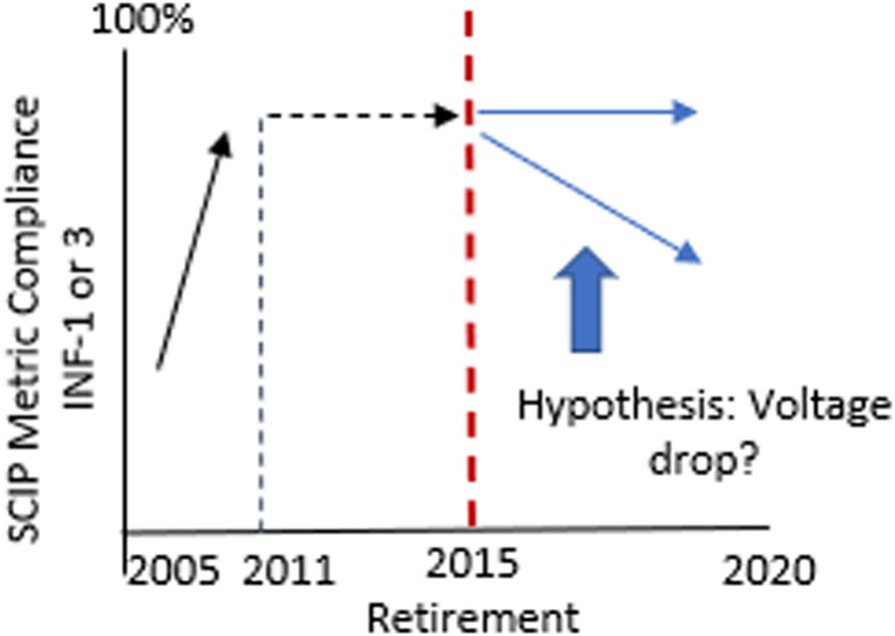
Theoretical “voltage drop” following discontinuation of the active Surgical Care Improvement Project

Relevant to the proposed protocol, after discontinuation of active implementation support, the DSF theorizes that programs may undergo *voltage drop*, which refers to the extent to which providers return to practices that were in place prior to an active implementation strategy that promoted successful change (Fig. 2) [18]. Figure 1 shows the main constructs of the DSF, including characteristics of the intervention, the practice setting, and the ecological system, and how they interact to impact sustainability over time. In the case of perioperative antimicrobial use and the SCIP program, there are two ways in which “voltage drop” may occur: (1) providers stop appropriately administering pre-incision antimicrobial prophylaxis, i.e., non-compliance with SCIP INF-1, and (2) providers may inappropriately prolong antimicrobials after skin closure, i.e., non-compliance with SCIP INF-3. The deviation from the intervention in the years SCIP was active (T_0_) to post-retirement (T_1_) may be due to features of the intervention itself, specialty-specific practice setting issues, and/or policy or other facility-level changes that make up the ecological system, such as surgical or infection control local guidelines.

**Fig. 3:**
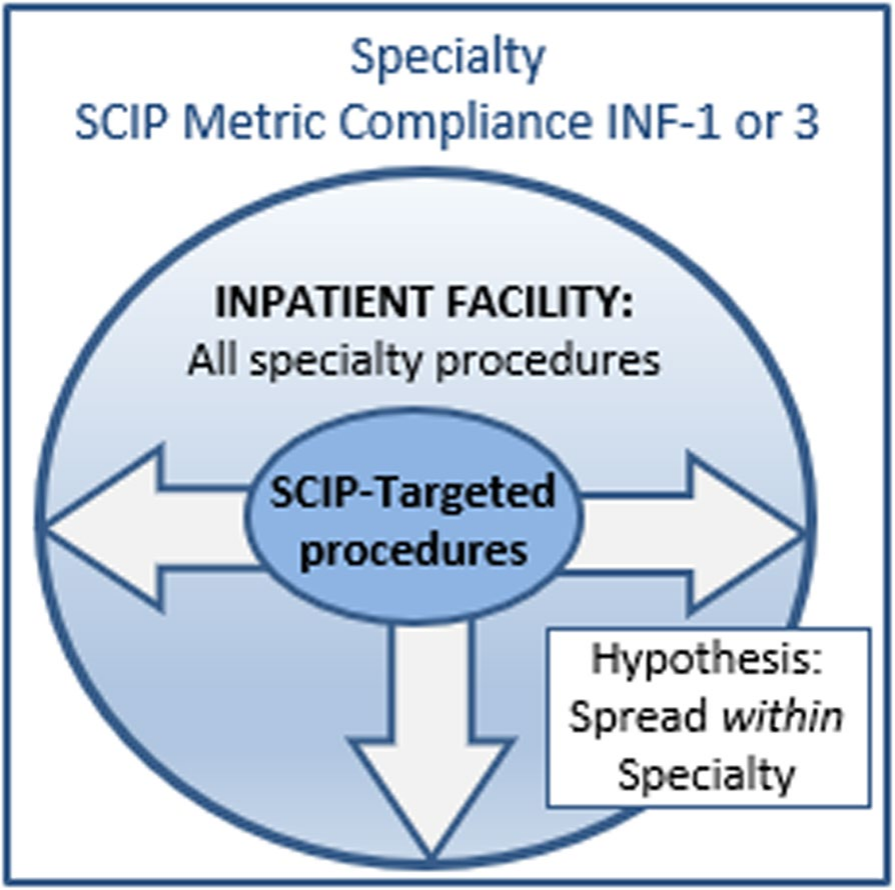
Diffusion or spread of evidence-based practice to uncovered procedures within the same specialty

The SCIP program primarily targeted only a limited number of high-risk surgeries within five surgical specialties, such as coronary artery bypass grafting, cardiac valve replacements, and orthopedic arthroplasties (total joint replacements). Other types of common but less invasive orthopedic procedures, such as arthroscopy and others, were not specifically targeted by all elements of the SCIP program. This raises the question about whether evidence-based antimicrobial practices promoted by the policy may have *diffused* within specialties. In other words, once orthopedic surgeons adopted a practice change for hip and knee replacements, was it also adopted for less invasive procedures not specifically targeted by the program? As described in the Diffusion of Innovation Theory, provider-level increases in adoption depend on awareness, persuasion, decision, implementation, and continuation [19]. Adoption of evidence-based practices may *spread* to procedures not covered by SCIP, which was limited in scope and primarily targeted a sub-set of major procedures most frequently performed in the Medicare population (Fig. 3). In other words, it is possible that practice change promoted by the program led to practice improvements for surgeries not included in its umbrella. For example, improvements may have been achieved through provider education or changes to preoperative protocols and order sets that were then used for surgeries not targeted by the program (Fig. 3). At the system level, the policies and practices may have changed for *all* surgical care and antimicrobial stewardship/infection control. For example, the Surgical Office or Infection Control may set policies or standards to effect change, such as surgical time outs that include a discussion of preoperative antimicrobial prophylaxis, and we may see consistently high compliance across all specialties in the inpatient facility. This possible spread beyond the initial program scope is consistent with themes highlighted in the Theory of Diffusion of Innovation, and distinct from “program drift” highlighted in the DSF, which focuses on changes to a program that can occur over time, rather than how practice improvements may have impacts beyond their initial targets [19]. These considerations lead to the hypothesis that compliance with SCIP INF-1 and 3 Metrics is Lower in SCIP-Excluded Procedures Compared to those Targeted by SCIP within a given specialty.

#### Gaps and rationale

Prior work demonstrates that while sustainability is an essential aspect of implementation science, few studies have measured factors associated with sustainability and how policy changes, such as the discontinuation of SCIP, impacted ongoing compliance with evidence-based practices. Guided by the DSF, this study will address these critically important translational research questions and will yield important insights about how to achieve sustained adoption of evidence-based practices after the active reporting program has ended. Informatics tools developed during the investigations will be adapted to measure compliance at the facility and specialty levels and will be included in an implementation playbook. Another major gap that will be addressed is an evaluation of how practice changes *spread* after an initial implementation directed at a small segment of clinical care; these aspects of the study will be guided by the Diffusion of Innovations theory. SCIP targeted major inpatient surgeries, but more minor procedures were not included in the antimicrobial use metrics. Lessons learned about factors driving the spread of practice change will provide critical insights for implementation science and will be included in our implementation playbook, one of the final products of this study.

## Methods and scientific approach

### Overall study design

**Fig. 4:**
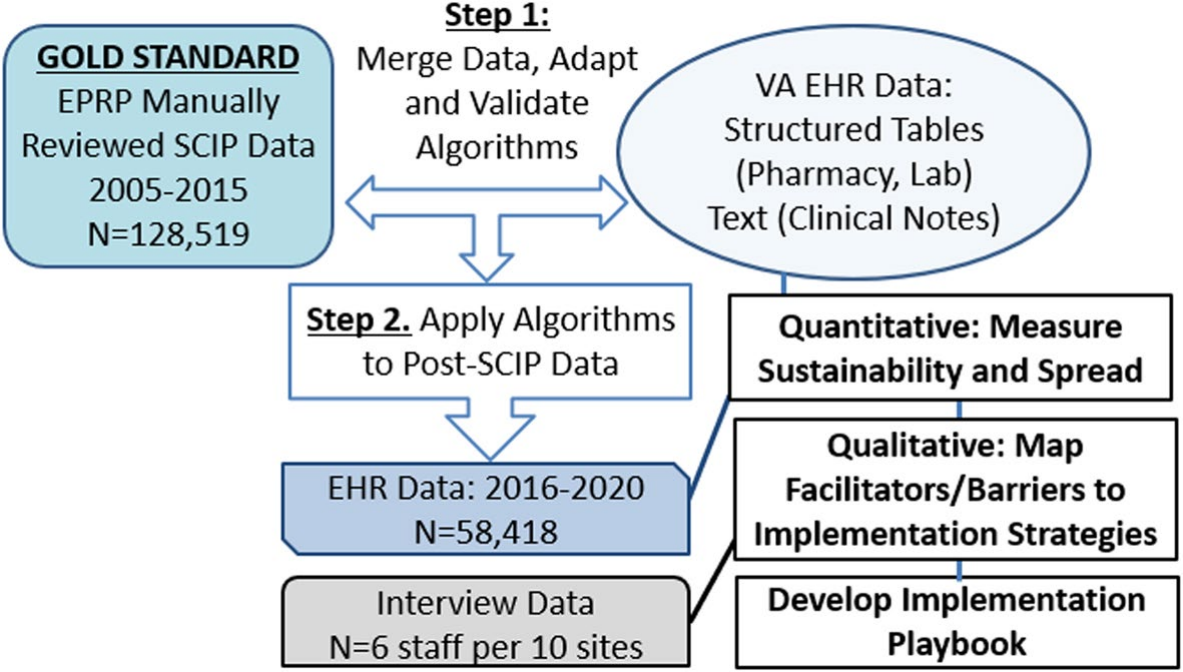
Project overview. Note: EPRP, External Peer Reviewed Program

**Fig. 5:**
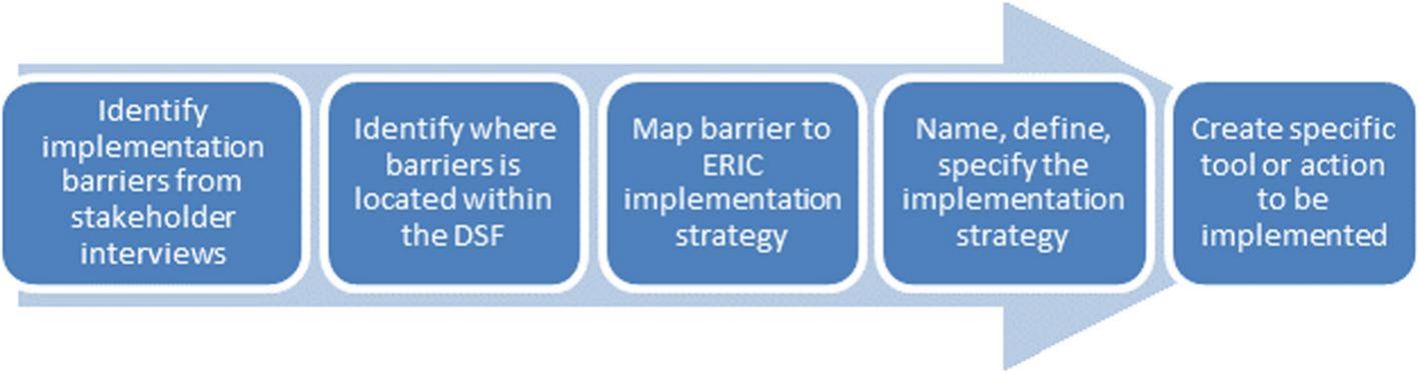
Process map for identifying barriers and linking them to evidence-based implementation strategies to inform future practice. DSF, Dynamic Sustainability Framework [11]; ERIC, Expert Recommendations for Implemention Change [20]

This study will use mixed methods sequential exploratory approach to address the study’s aims, which are to assess the sustainability and spread of appropriate pre- and postoperative antimicrobial use achieved by the SCIP program (Fig. 4). The research will adhere to the STROBE reporting checklist. Guided by the DSF, the final product of this study will yield an implementation playbook that will comprehensively describe for future implementation sites the need for adapting SCIP metrics and implementation strategies to fit local practice settings and the local environment (ecological system) (Fig. 5) as well as the different evidence-based implementation strategies (ERIC) [20] that can be used to support program sustainability.

### Quantitative data collection and analysis

#### Study population and setting

VA surgical data from FY 2005 to 2020 from approximately 70 complex VA inpatient facilities will potentially be used to complete the quantitative analysis. This large, national dataset will include inpatient and outpatient clean or clean contaminated surgeries in cardiac, orthopedic, general, gynecology, and vascular specialties as defined by Current Procedural Terminology (CPT) codes. Surgical data will be combined with manually validated data about perioperative antimicrobial use collected from the External Peer Reviewed Program (EPRP) dataset. Thus, the EPRP data can be used as the “gold standard” manual review for the purposes of developing, iteratively refining, and validating electronic measurement tools that do not require the time and resource intensive process the original SCIP program required.

#### Development cohort

After obtaining EPRP data on SCIP compliance by facility and specialty, SCIP-eligible procedures will be identified in the VA electronic health record (EHR) from 2005 to 2015 to build a dataset for each specialty cohort with SCIP compliance information and structured/unstructured data relevant to our SCIP INF-1 and 3 algorithms. EPRP data contains manually reviewed surgeries targeted by SCIP, and based on previously published work, we anticipate that our sample will include many non-compliant cases, particularly in the early period following program implementation.

#### Algorithm development

First, a list of appropriate antimicrobials for surgical care as specified in the original SCIP guidelines and in current multi-society SSI prevention guidelines will be reviewed and mapped for each of the included surgical specialties [5, 21, 22]. Then, this list will be used to develop electronic measurement tools. Based on our prior work [23], we will develop each algorithm to detect SCIP INF-1 and 3 antimicrobial prophylaxis iteratively over several stages, by varying (1) types of variables included in the tool (e.g., text note extraction only, orders only, administration only, or combinations of the three), (2) timing of the searches (e.g., including or excluding the procedure date), and (3) types of antimicrobials included in the tool. The list will be used to search clinical notes for documentation of antibiotic administration pre- and/or postoperatively to measure compliance with SCIP metrics. The list will also be mapped to structured data in the VA EHR and orders and administration of relevant medications will be extracted.

The two algorithms (one for preoperative antimicrobial administration and one for postoperative administration) will be applied to half of all EPRP reviewed surgeries targeted by SCIP from 2005 to 2015. Algorithm performance, i.e., criterion validity, will be assessed as sensitivity (how many true positive cases were identified as positive by the algorithm) and specificity (how many true negative cases were identified as negative by the algorithm). Manually reviewed EPRP data will be used as the gold standard for algorithm development. To finalize the algorithm, a sample of discordant cases (e.g., algorithm flagged positive but EPRP manual review was negative, or algorithm flagged negative but EPRP manual review was positive) will undergo a second round of manual review to identify reasons for the discordant flag and to qualitatively classify the reason for discordance. These findings will be used to adjust and adapt the algorithms for each SCIP metric and each specialty procedure and facility to improve accuracy. We will also conduct an analysis stratified by facility, to determine if facility-level effects, such as coding differences, impact algorithm performance and accuracy.

#### Algorithm validation

We will validate the final SCIP INF-1 and 3 algorithms by applying the structured and unstructured data extracts to SCIP-eligible procedures in the validation half of the 2005–2015 EPRP data. We will measure and criterion validity: sensitivity, specificity, and positive predictive validity for each algorithm per specialty (cardiac, orthopedics, general, gynecology, and vascular surgeries). Clinical documentation practices in the EHR may have evolved over time, thus algorithm performance may be substantially different in 2005. If we observe this, we will train and validate the algorithm with the smaller sample of more recent data, but retain the earlier, already manually reviewed dataset for analyzing scale-up, spread, and sustainability of the policy.

#### Assessment of voltage drop: analysis (interrupted time series)

After the algorithms for measuring pre-and postoperative antimicrobial use are validated, we will then apply them to the 2016–2020 period, e.g., after SCIP discontinuation and after discontinuation of the EPRP manual review. SCIP INF-1 and 3 compliance stratified by specialty will be assessed separately.

We will test the hypotheses that SCIP INF-1 and 3 compliance experienced “voltage drop,” defined as decreasing adherence to antimicrobial use guidelines over time, after SCIP retirement in 2015 and an overall change in rate over time using an interrupted time series model. Observations will be at the procedure level with facility repeated measures; thus, we will control for facility-level random effects. The outcome measure will be a binary indicator of compliance for each procedure, and the models will include a binary indicator of pre- or post-SCIP retirement, a continuous variable for calendar time, and an interaction between these two variables. The beta coefficient for the interaction term will represent the difference in slopes between pre- and post-SCIP retirement, the coefficient for pre and post will indicate the “voltage drop,” or immediate drop after the SCIP retirement, and the coefficient for time will indicate the slope for the pre-period. For each outcome, SCIP INF-1 and 3, and for each surgical specialty, we will fit separate models. To adjust for multiple comparisons, we will perform false discovery rate (FDR) correction on the betas for the interaction terms for each of the 10 comparisons. For each of the 10 comparisons, setting alpha at 0.01 to be conservative (given that we will adjust for multiple comparisons), assuming there will be correlation among patients from the same facilities (0.02), we can detect at least a 5% difference in slopes between pre- and post-SCIP retirement for each surgical specialty with greater than 90% power. This assumes approximately 95% SCIP compliance between 2011 and 2015 [9].

#### Assessment of diffusion of practices to surgeries not covered by SCIP

SCIP active reporting demonstrably increased appropriate antimicrobial prophylaxis compliance in SCIP-targeted procedures, largely due to changes in provider behavior that may have been in response to local policies designed to facilitate adoption. With respect to diffusion, the provider behavior change for one type of surgery may have led to changes for all procedures, not just those specifically targeted by SCIP.

#### Diffusion of practices cohort

All clean or clean/contaminated surgical procedures within the five specialties performed from 2016 to 2020 will be identified and an expanded cohort will be created. Our analysis will be limited to surgeries where preoperative antibiotics are recommended.

#### Statistical analysis

We will use binomial generalized linear mixed models to estimate the association between SCIP-targeted versus excluded procedures and the two SCIP metrics, adjusting for correlation among observations within the same facility using facility random effects. The variance-covariance matrix will allow us to estimate the correlation coefficients for nesting so that we can make inferences on the strength of these sources of correlation. Separate models will be fit for each outcome (SCIP INF-1 and 3) and surgical specialty, a total of 10 models. We will again apply FDR correction on the *p*-values for the primary comparisons related to our hypothesis.

#### Power calculation

For each of the 10 models, we will have greater than 90% power to detect at least a 10% difference in the two SCIP compliance outcome measures between the comparison groups for each hypothesis. This assumes correlation of 0.02 at the facility level and alpha set at 0.01. For example, if compliance is 85% for females and 75% for males, we can detect this difference with an *effective* sample size of 938 individuals.

### Qualitative data collection and analysis

#### Overview of qualitative analysis

For the qualitative aspects of the study, based on current guidance [24], we will interview up to six key stakeholders in different VA hospitals for each of the five specialties, for a total of 60 interviews in ten VA facilities. Interview participants will have work duties related to SSI prevention and antimicrobial stewardship: surgical staff (including anesthesiologists), infectious diseases staff, pharmacists, and surgical nurses. We will analyze interview data to identify facilitators and barriers to implementation sustainability and map findings to DSF constructs. Practice spread will also be assessed and evaluated using the Diffusion of Innovations Theory as a guide. As a final step, we will map facilitators and barriers to the ERIC implementation strategies and will develop an implementation playbook that can be used by VA hospitals to support long-term sustainability of the quality improvements in perioperative antimicrobial use achieved by SCIP.

#### Key stakeholders

Key stakeholders for perioperative antimicrobial use include members of the surgical staff (surgeons, anesthesiologists, nurses), infectious diseases staff (physicians and infection control/antimicrobial stewardship team members), and inpatient pharmacists. These are the providers who will be targeted at each of the participating VA facilities.

#### Selection of sites and recruitment

We will purposefully recruit two facilities for each specialty (*N*=10 facilities) from the 70 high complexity VA facilities, targeting a range of sites, including regional diversity and urban, suburban, and rural variation. Through this selection process, we will recruit 60 stakeholders from 10 facilities (6 interview participants at each site). Our goal is to reach saturation within each of the five SCIP-targeted specialties across the sites; typical sample sizes for achieving this in implementation research range from 5 to 10 individuals in key roles [25].

#### Recruitment

Operational partners from the national patient safety office and the national antimicrobial stewardship office will co-sign a letter outlining support for the project; the PIs will then send out this letter to a purposefully sampled selection of sites and inform them of the study prior to sending recruitment emails to providers. Thereafter, the project manager will send out recruitment emails to providers. We will use an opt-in approach. If we do not get enough stakeholders who agree to participate, then we will identify additional stakeholders to recruit using the approach described above.

#### Data collection

Two co-investigators will conduct semi-structured telephone or video interviews with key stakeholders; interviews are estimated to take approximately 30 min. All interviews will be digitally audio-recorded for transcription; informed consent will be obtained prior to starting the interview. Interviewers will follow a semi-structured interview guide, which will consist of both structured and open-ended questions. The interview guide will be revised with input from operational partners prior to pilot testing and data collection. Through this process, we will ensure that we develop a set of questions that will allow us to collect rich information about facilitators and barriers of sustainability and also about factors that lead to diffusion of the practice changes, or lack of spread.

Informed by DSF and the Theory of Diffusion of Innovations, interviews will elicit information about the intervention (SCIP), the practice setting/context (surgical specialty), and the ecological system (VA facility/VISN). The overall purpose of the interviews will be to understand, from the perspectives of surgical staff, infectious diseases staff, and inpatient pharmacists, what types of processes/practices have been implemented to help with perioperative and postoperative antimicrobial use and compliance; whether and how those processes/practices were adapted and sustained after SCIP was retired in 2015; whether and how those antimicrobial use and compliance processes/practices spread to other settings; and the facilitators that helped with and the barriers that hindered implementation, maintenance, and spread of those processes/practices. Information will be collected about the contextual factors within the practice setting that affect implementation and sustainment of antimicrobial use and compliance by asking about the culture of the practice setting as well as trainings and resources that are available to help with antimicrobial use and compliance. Additionally, interviews will probe to understand whether there are other metrics, policies, regulations, or guidelines that are being used for perioperative and postoperative antimicrobial compliance and how those have influenced processes/practices.

#### Qualitative data analysis

Coding of interview transcripts will be organized using NVivo, a qualitative analytic software. Transcripts will be initially coded using a priori constructs consistent with the DSF and/or the Diffusion of Innovations theory. We will use a directed content analysis approach with allowance for new themes to emerge [26]. As coding proceeds, new emergent themes will iteratively be identified, elaborated on, and expanded based on team discussions, a process known as the constant comparative method. Inter-rater reliability will be established using the “check-coding” process. Coders will independently code the same interview transcripts and will then meet to compare their coding, discuss areas of difficulty, and reach consensus on the definitions and examples in the codebook. A new interview will then be independently coded by all, and the process will be repeated until a mutual understanding of the code definitions and when to apply the codes is achieved across all coders. After coding is complete, we will summarize the data by producing site-specific descriptive summaries, which will include key information (quotes and themes) about our findings for each of the DSF constructs—the intervention (SCIP), the practice setting/context, and the ecological system. Within the site-specific summaries, we will note any differences in perspectives between key stakeholders at the facility as well as differences by specialty. The site summaries will result in a rich description of each DSF constructs and the factors (e.g., facilitators and barriers) that affect implementation sustainability of SCIP.

#### Triangulation of qualitative and quantitative data

When site-specific summaries are complete, we will triangulate our quantitative and qualitative findings. Utilizing Miles and Huberman’s analytical approaches [27], we will triangulate the quantitative data elements from the facilities and specialties and the compliance rates with the qualitative findings to create a cross-site matrix. We will compare and contrast evidence to determine the key factors that may affect implementation sustainability of SCIP for sites with high or low SCIP compliance as well as for different specialties. We will then develop descriptive cross-site summaries based on our analysis of the integrated data.

#### Mapping of findings to Expert Recommendations for Implementation Change (ERIC) implementation strategies

The data matrices will be used to map DSF-defined barriers and facilitators to the evidence-based list of implementation strategies developed by the Expert Recommendations for Implementation Change (ERIC) group [20, 28]. Table 1 provides examples of implementation barriers we may find related to specific DSF constructs, and the selection and specification of ERIC implementation strategies that may be identified as a result of our mapping process (Fig. 5). This process will identify and specify implementation strategies to address relevant challenges particular to pre- and postoperative antimicrobial use and compliance, and will inform the creation of an implementation playbook.

**Table 1:** Process for mapping sustainability barriers and facilitators to ERIC implementation strategies for inclusion in implementation playbook

Formative evaluation barrier/facilitator	DSF construct	Example ERIC implementation strategy	Definition of implementation strategy	Example tool or action to be included in playbook
Lack of knowledge about pre-incision prophylaxis, and harms associated with prolonged prophylaxis	Providers involved in intervention (intervention)	Conduct educational meeting	Hold meetings targeted toward different stakeholder groups to teach them about the clinical innovation	Educational sessions to focus on developing these skills
Concern about “standard of care” and lack of resources to comply with established practices	Staffing, training (practice setting/context)	Conduct ongoing training Change clinic system	Plan for and conduct training in the innovation in an ongoing way Change clinic systems to allow better assessment of implementation or clinical outcomes	Development of webinar and in-person trainings, technology platform to provide new method of collaborating with surgical and infectious disease colleagues across sites
Concern about lack of national-level policy and surveillance program to detect infections	Population characteristics (ecological system)	Develop and distribute educational material	Develop and format manuals, toolkits, and other supporting materials in ways that make it easier for stakeholders to learn about the innovation	FAQ sheets for patients, family members, staff, clinicians prior to Hybrid Type III testing of implementation strategies for sustaining SCIP
Availability of Surgical Checklists, Protocols, and Order sets (facilitator)	Components (intervention)	Capture and share local knowledge	Capture local knowledge from sites on how clinicians made something work in their setting and share it with other sites	Example protocols that can be locally adapted and disseminated; Sharepoint site with resources for facilities

*ERIC* Expert Recommendations for Implementation Change [20]

Following completion of the triangulation of qualitative and quantitative data, and mapping of findings to ERIC implementation strategies, we will create an implementation playbook: a document that comprehensively describes how to sustain SCIP best practices in sites with low compliance on either of the SCIP antimicrobial use metrics; different implementation strategies may be required depending upon whether sites have low sustainability with SCIP INF 1, 3, or both. The playbook will reflect the DSF in describing to sites the need to adapt SCIP antimicrobial use metrics and the ERIC implementation strategies to fit local practice settings, the local environment, and Veteran populations (ecological system). Examples of sections that may be included in the implementation playbook are as follows: (1) evidence base for the SCIP metrics; (2) advice on communicating with facility leadership and staff about SCIP to generate interest; (3) assessment tools for sites to gauge their readiness; (4) planning tools, such as timelines to ensure appropriate rollout of relevant interventions; (5) operational tools, such as example policies, checklists, and example electronic order sets that can be locally adapted; (6) training curricula and educational materials for SCIP site champions to train other staff; and (7) a plan for supporting ongoing measurement through local adaptation and calibration of the informatics tools developed in our quantitative analyses, and measurement of clinical outcomes, including SSIs, and other adverse events. All of these elements are necessary to support future uptake and sustainment of guideline concordant antimicrobial use.

To ensure the implementation playbook is operationally useful, we will seek feedback from local and national stakeholders using a member checking process [29]. Member checking, also known as participant or respondent validation, is a technique for exploring the credibility of research results. This will ensure that the playbook has been developed as intended to increase the uptake and sustainability of SCIP antimicrobial use metrics at sites with low compliance with one or both of the INF metrics. We will interview a subset of our respondents from the qualitative interviews (*n*∼20) to assess the perceived acceptability and feasibility of elements of the draft implementation playbook, selected implementation strategies, and locations for targeting the sustainability, spread, and diffusion of SCIP practices. Using data collection and analysis methods previously described, we will identify areas of the playbook that require further changes and updates.

## Discussion and limitations of the approach

We will use electronic data and health informatics tools (i.e., data mining through text queries and algorithm development/validation) to measure guideline compliance with surgical antimicrobial prophylaxis. Lessons learned about the sustainability and diffusion of evidence-based practices will have a direct impact on VA surgical care and will advance the field of implementation science through improving our knowledge and understanding about factors that influence sustainability, a historically understudied, yet critically important area. In addition to the contributions to the advancement of implementation science, healthcare informatics tools adapted and optimized will lead to expansion of antimicrobial stewardship efforts to include a broad range of surgical types and specialties and are likely to directly translate into improvements in clinical care delivery.

There are several limitations to our approach to assessing both sustainment and spread of SCIP practices. First, this study will gather retrospective data from the EHR; it is not a prospective or interventional study. However, for the purposes of evaluating the long-term impact of a policy change, this is an appropriate study design and the process of mapping barriers to strategies will provide valuable data to inform the development of implementation bundles and toolkits for testing in a future clinical trial. Second, a major hypothesis of our study is that voltage drop has occurred over time, but it is possible that we may not identify substantial variation and that all sites will have maintained outstanding compliance with SCIP metrics. If this occurs, the qualitative aspects of the proposal will be used to gather information about factors that contributed to the long-term sustainability of the practice change so that the effective strategies can be replicated or tested in other clinical settings. Data about why providers did not revert to outdated standards of care are important for advancing the field of implementation science and may promote similar improvements in other settings of care. Thus, we expect to still be able to collect rich and useful qualitative interview data, even if voltage drop has been minimal and/or if variation between sites is less than anticipated.

## Conclusions

Our protocol presents an innovative approach to improving surgical care and antimicrobial stewardship by combining existing electronic data and health informatics tools (i.e., data mining through text queries and algorithm development/validation) to measure guideline compliance with surgical antimicrobial prophylaxis. Lessons learned about the sustainability and diffusion of evidence-based practices will have a direct impact on surgical care. Healthcare informatics tools adapted and optimized as part of the research will lead to expansion of antimicrobial stewardship efforts to include a broad range of surgical types and specialties.

## Supplementary Information

**Additional file 1.**

## Data Availability

Not applicable.
