# Supplementary material for: Assessing the sustainability of compliance with surgical site infection prophylaxis after discontinuation of mandatory active reporting: study protocol
Source: Implement Sci Commun. 2022 Apr 25;3:47. doi: 10.1186/s43058-022-00288-0 (PMC9036843; doi:10.1186/s43058-022-00288-0)
Supplement: Supplementary file 1 — Additional file 1. [file 43058_2022_288_MOESM1_ESM.pdf]

OMB Number: 4040-0010
Expiration Date: 12/31/2022

APPLICATION FOR FEDERAL ASSISTANCE
SF 424 (R&R)

**3. DATE RECEIVED BY STATE**	**State Application Identifier**

1. TYPE OF SUBMISSION*

❍ Pre-application ❍ Application ● Changed/Corrected Application

4.a. Federal Identifier
HX003231

b. Agency Routing Number
523-Boston

2. DATE SUBMITTED
2020-05-28

Application Identifier

c. Previous Grants.gov Tracking Number
GRANT13116745

5. APPLICANT INFORMATION **Organizational DUNS*:** 0344322650000

Legal Name*:	VA BOSTON HEALTH CARE SYSTEM
Department:	
Division:	
Street1*:	RESEARCH ADMINISTRATION (151B)
Street2:	150 SOUTH HUNTINGTON AVE
City*:	BOSTON
County:	
State*:	MA: Massachusetts
Province:	
Country*:	USA: UNITED STATES
ZIP / Postal Code*:	021304817

Person to be contacted on matters involving this application

Prefix: First Name*: Niquel Middle Name: Last Name*: Ortega Suffix:

Position/Title:	Adminstrative Officer
Street1*:	150 South Huntington Ave
Street2:	
City*:	Boston
County:	
State*:	MA: Massachusetts
Province:	
Country*:	USA: UNITED STATES
ZIP / Postal Code*:	02130-4817

Phone Number*: 857-364-5669 Fax Number: 857-364-4486 Email: Niquel.Ortega@va.gov

6. EMPLOYER IDENTIFICATION NUMBER *(EIN) or (TIN)** 741612229

7. TYPE OF APPLICANT* X: Other (specify)

Other (Specify): VA-ORD

Small Business Organization Type ❍ Women Owned ❍ Socially and Economically Disadvantaged

8. TYPE OF APPLICATION*

❍ New ● Resubmission
❍ Renewal ❍ Continuation ❍ Revision

If Revision, mark appropriate box(es).

❍ A. Increase Award ❍ B. Decrease Award ❍ C. Increase Duration
❍ D. Decrease Duration ❍ E. Other *(specify)* :

Is this application being submitted to other agencies?* ❍Yes ●No What other Agencies?

9. NAME OF FEDERAL AGENCY*
Office of Research and Development

10. CATALOG OF FEDERAL DOMESTIC ASSISTANCE NUMBER
64.054
TITLE: Research and Development

11. DESCRIPTIVE TITLE OF APPLICANT'S PROJECT*
Assessing the Sustainability of Compliance with Surgical Site Infection Prophylaxis After Discontinuation of Mandatory Active Reporting

12. PROPOSED PROJECT

Start Date*	Ending Date*
01/01/2021	12/31/2024

13. CONGRESSIONAL DISTRICTS OF APPLICANT
MA-007

SF 424 (R&R) APPLICATION FOR FEDERAL ASSISTANCE

14. PROJECT DIRECTOR/PRINCIPAL INVESTIGATOR CONTACT INFORMATION

Prefix: First Name*: Hillary Middle Name: Jane Last Name*: Mull Suffix:

Position/Title: Investigator

Organization Name*: VA Boston Healthcare System

Department:

Division:

Street1*: 150 South Huntington Ave

Street2:

City*: Boston

County:

State*: MA: Massachusetts

Province:

Country*: USA: UNITED STATES

ZIP / Postal Code*: 02130-4817

Phone Number*: 857-364-2766 Fax Number: Email*: hillary.mull@va.gov

15. ESTIMATED PROJECT FUNDING

a. Total Federal Funds Requested*	$1,198,134.00
b. Total Non-Federal Funds*	$0.00
c. Total Federal & Non-Federal Funds*	$1,198,134.00
d. Estimated Program Income*	$0.00

16.IS APPLICATION SUBJECT TO REVIEW BY STATE EXECUTIVE ORDER 12372 PROCESS?*

a. YES ❍ THIS PREAPPLICATION/APPLICATION WAS MADE AVAILABLE TO THE STATE EXECUTIVE ORDER 12372 PROCESS FOR REVIEW ON:

DATE:

b. NO ● PROGRAM IS NOT COVERED BY E.O. 12372; OR

❍ PROGRAM HAS NOT BEEN SELECTED BY STATE FOR REVIEW

17. By signing this application, I certify (1) to the statements contained in the list of certifications* and (2) that the statements herein are true, complete and accurate to the best of my knowledge. I also provide the required assurances * and agree to comply with any resulting terms if I accept an award. I am aware that any false, fictitious, or fraudulent statements or claims may subject me to criminal, civil, or administrative penalties. (U.S. Code, Title 18, Section 1001)

● I agree*

** The list of certifications and assurances, or an Internet site where you may obtain this list, is contained in the announcement or agency specific instructions.*

18. SFLLL or OTHER EXPLANATORY DOCUMENTATION File Name:

19. AUTHORIZED REPRESENTATIVE

Prefix: First Name*: Niquel Middle Name: Last Name*: Ortega Suffix:

Position/Title*: Administrative Officer

Organization Name*: VA Boston Healthcare System

Department: Research and Development

Division:

Street1*: 150 South Huntington Ave

Street2:

City*: Boston

County:

State*: MA: Massachusetts

Province:

Country*: USA: UNITED STATES

ZIP / Postal Code*: 02130-4817

Phone Number*: 857-364-5669 Fax Number: 857-364-4486 Email*: Niquel.Ortega@va.gov

**Signature of Authorized Representative***	**Date Signed***
Niquel Ortega	05/28/2020

20. PRE-APPLICATION File Name:

21. COVER LETTER ATTACHMENT File Name:

424 R&R
Table Of Contents

SF 424 R&R Cover Page 1
Table of Contents 3
Performance Sites 4
Research & Related Other Project Information 6
Project Summary/Abstract(Description) 7
Project Narrative 8
Bibliography & References Cited 9
Facilities & Other Resources 13
Other Attachments 14
Introduction to Revised Application 14
Specific Aims 17
Research Plan 18
Human Subjects 32
Multiple Leadership Plan 35
VA Medical Center Director Letter 36
Letters of Support 37
Data Management And Access Plan (DMAP) 42
Financial Disclosure 46
Appendix 1 Index 47
Appendix 2 Abbreviations 48
Appendix 3 SCIP_ICD-9_Procedure_Codes 49
Appendix 4 Summary_of_Existing_Studies 52
Appendix 5 Abx_Algorithm_CIED 53
Appendix 6 Interview_Guide 54
Research & Related Senior/Key Person 56
Research & Related Budget Year - 1 116
Research & Related Budget Year - 2 119
Research & Related Budget Year - 3 122
Research & Related Budget Year - 4 125
Budget Justification 128
Research & Related Cumulative Budget 133

OMB Number: 4040-0010
Expiration Date: 12/31/2022

Project/Performance Site Location(s)

Project/Performance Site Primary Location

● I am submitting an application as an individual, and not on behalf of a company, state, local or tribal government, academia, or other type of organization.

Organization Name: VA BOSTON HEALTH CARE SYSTEM
Duns Number: 0344322650000
Street1*: RESEARCH ADMINISTRATION (151B)
Street2: 150 SOUTH HUNTINGTON AVE
City*: BOSTON
County:
State*: MA: Massachusetts
Province:
Country*: USA: UNITED STATES
Zip / Postal Code*: 021304817
Project/Performance Site Congressional District*: MA-007

Project/Performance Site Location 1

❍ I am submitting an application as an individual, and not on behalf of a company, state, local or tribal government, academia, or other type of organization.

Organization Name: Edith Norse Rogers Memorial Hospital
DUNS Number: 0800423360000
Street1*: 200 Veterans Ave
Street2:
City*: Bedford
County:
State*: MA: Massachusetts
Province:
Country*: USA: UNITED STATES
Zip / Postal Code*: 01730-1114
Project/Performance Site Congressional District*: MA-006

Project/Performance Site Location 2

❍ I am submitting an application as an individual, and not on behalf of a company, state, local or tribal government, academia, or other type of organization.

Organization Name:	Palo Alto Health Care System
DUNS Number:	0460174550000
Street1*:	3801 Miranda Ave
Street2:	
City*:	Palo Alto
County:	
State*:	CA: California
Province:	
Country*:	USA: UNITED STATES
Zip / Postal Code*:	94304-1207
Project/Performance Site Congressional District*:	CA-014

Project/Performance Site Location 3

❍ I am submitting an application as an individual, and not on behalf of a company, state, local or tribal government, academia, or other type of organization.

Organization Name:	University of Colorado Denver - Anschutz Medical Center
DUNS Number:	0410963140000
Street1*:	13001 East 17th Place
Street2:	
City*:	Aurora
County:	
State*:	CO: Colorado
Province:	
Country*:	USA: UNITED STATES
Zip / Postal Code*:	80045-0000
Project/Performance Site Congressional District*:	CO-006

Additional Location(s) File Name:

OMB Number: 4040-0010
Expiration Date: 12/31/2022

RESEARCH & RELATED Other Project Information

1. Are Human Subjects Involved?* ● **Yes** ❍ **No**

1.a. If YES to Human Subjects

Is the Project Exempt from Federal regulations? ❍ Yes ● No

If YES, check appropriate exemption number: __ 1 __ 2 __ 3 __ 4 __ 5 __ 6 __ 7 __ 8

If NO, is the IRB review Pending? ● **Yes** ❍ **No**

IRB Approval Date:

Human Subject Assurance Number 00001270

2. Are Vertebrate Animals Used?* ❍ **Yes** ● **No**

2.a. If YES to Vertebrate Animals

Is the IACUC review Pending? ❍ Yes ❍ No

IACUC Approval Date:

Animal Welfare Assurance Number

3. Is proprietary/privileged information included in the application?* ❍ **Yes** ● **No**

4.a. Does this project have an actual or potential impact - positive or negative - on the environment?* ❍ **Yes** ● **No**

4.b. If yes, please explain:

4.c. If this project has an actual or potential impact on the environment, has an exemption been authorized or an environmental assessment (EA) or environmental impact statement (EIS) been performed? ❍ Yes ❍ No

4.d. If yes, please explain:

5. Is the research performance site designated, or eligible to be designated, as a historic place?* ❍ **Yes** ● **No**

5.a. If yes, please explain:

6. Does this project involve activities outside the United States or partnership with international collaborators?* ❍ **Yes** ● **No**

6.a. If yes, identify countries:

6.b. Optional Explanation:

	Filename
**7. Project Summary/Abstract***	06_VA_Project_Summary.pdf
**8. Project Narrative***	07_VA_Project_Narrative.pdf
**9. Bibliography & References Cited**	VA_Bibliography.pdf
**10. Facilities & Other Resources**	VA_Facilities_Other_Resources.pdf
**11. Equipment**	
**12. Other Attachments**	01_VA_Intro.pdf<br>02_VA_Specific_Aims.pdf<br>02a_VA_Research_Plan.pdf<br>04_VA_Human_Subjects.pdf<br>06_VA_Multiple_PI.pdf<br>08_VA_Director_Letter.pdf<br>08b_VA_Letters_of_Support.pdf<br>09_VA_DMAP.pdf<br>10_VA_Financial_Disclosure.pdf<br>11_VA_Appendix_1_Index.pdf<br>12_VA_Appendix_2_Abbreviations.pdf<br>13_VA_Appendix_3_SCIP_ICD-9_Procedure_Codes.pdf<br>14_VA_Appendix_4_Summary_of_Existing_Studies.pdf<br>15_VA_Appendix_5_Abx_Algorithm_CIED.pdf<br>16_VA_Appendix_6_Interview_Guide.pdf

Background: SSIs are common and preventable adverse surgical outcomes that account for substantial morbidity and mortality. Pre-operative antimicrobial administration reduces SSI; post-operative antimicrobials do not reduce SSI but do increase other post-operative adverse events. In 2005, the VA implemented the Surgical Care Improvement Project (SCIP) to increase compliance with peri-operative quality metrics, including appropriate antimicrobial use. SCIP included a set of publicly reported evidenced-based antimicrobial guideline compliance metrics targeting high-risk surgeries. The metrics required resource-intense manual review as part of the VA's External Peer Review Program (EPRP). In 2015, SCIP achieved sustained compliance with antimicrobial use metrics exceeding 95% and the program was retired due to the high costs of manual review with minimal expected additional benefit. This IIR proposal is guided by the *Dynamic Sustainability Framework* and examines whether the antimicrobial practice changes achieved by SCIP were *sustained* and how they *spread* to surgeries and practice areas beyond the original scope of the program. This multiple PI submission by Dr. Hillary Mull, PhD and Dr. Westyn Branch-Elliman, MD, MMSc builds and expands upon their prior VA-funded collaborative work applying electronic algorithms to measure antimicrobial use and identify adverse outcomes.

Significance/Impact: Improving antimicrobial prescribing and surgical quality are major VA goals. In FY 2018, within the VA, there were 190,000 surgeries among SCIP-targeted specialties (cardiac, orthopedic, general/colorectal, gynecology and vascular). Of these, 64% were outpatient, where the PIs found high rates guideline discordant post-operative antimicrobial use, which contribute to substantial patient harms. Thus, the potential impact of the research on direct clinical care is high. In addition, there are limited studies examining what happens to practice change after discontinuation of active programs designed to support compliance with evidence-based guidelines. The proposed study will close this gap in implementation science research.

Innovation: In line with the VA's Learning Health System directive, this project will use the VA's electronic health record data to develop algorithms to identify pre-and post-operative antimicrobial use based on the PI's prior work. These electronic data mining algorithms capture structured and text note data and represent a significant technological advancement over costly manual review. This study also answers important questions about the sustainability of practice change after discontinuation of an active policy with input from frontline staff.

Specific Aims: 1) Measure sustainability of antimicrobial prophylaxis guideline compliance after SCIP retirement and assess whether practice changes spread to SCIP-excluded procedures; 2) Assess facilitators and barriers to implementation sustainability through staff interviews, and map results to implementation strategies; 3) Develop an implementation playbook with assessment tools, training curricula and other elements to support scale-up and spread of evidence-based antimicrobial practices in a future HSR&D Hybrid III trial.

Methodology: Aim I will adapt existing informatics algorithms to extract pre-and post-operative antimicrobial use from clinical note texts and antimicrobial orders for each of the SCIP surgeries. Previously manually reviewed 2005-2015 EPRP data will be used as the gold-standard for adapting and refining the data mining tools. SCIP sustainability will be measured by applying the validated antimicrobial compliance algorithms to 2005-2020 data and performing interrupted time series analyses. SCIP spread within specialties will be assessed with regression models. Aim II will use qualitative interviews with frontline staff to identify reasons for sustainability of evidence-based practice using constructs in the Dynamic Sustainability Framework and will triangulate qualitative and quantitative data to ascertain the long-term impacts of SCIP. Aim III will develop an implementation playbook with input from our Operational Steering Committee comprised of national and local VA surgical stakeholders.

Next Steps/Implementation: Test implementation playbook in a Hybrid Type III study to improve practice.

Surgical site infections are common and morbid; however, they are preventable with administration of evidence-based antimicrobial prophylaxis. In 2005, in the setting of high rates of non-compliance with antimicrobial prophylaxis guidelines, the VA implemented the Surgical Care Improvement Project (SCIP). SCIP included public reporting of evidenced-based antimicrobial guideline compliance metrics in high-risk surgeries and led to high rates of adoption of evidence-based practices (>95%). The program was retired in 2015, as the manual measurement and reporting process was costly with limited expected additional benefit. This IIR will use novel electronic algorithms to measure compliance with SCIP metrics over time, and stakeholder feedback to identify intervention strategies to sustain compliance. The aims are guided by the Dynamic Sustainability Framework and will address 1) the sustainability and spread of practice changes achieved by SCIP, 2) factors influencing sustainability, and 3) implementation strategies to test in a future Hybrid III trial to improve compliance.

References Cited:

1. Kilbourne AM, Goodrich DE, Miake-Lye I, et al. Quality Enhancement Research Initiative Implementation Roadmap: Toward Sustainability of Evidence-based Practices in a Learning Health System. *Medical care.* 2019;57 Suppl 10 Suppl 3(10 Suppl 3):S286-S293.
2. VA National Surgery Office (NSO). Surgical Services Homepage. http://vaww.dushom.va.gov/surgery/index.asp. Published 2019. Accessed June 10, 2019.
3. Maguire EM, Bokhour BG, Wagner TH, et al. Evaluating the implementation of a national disclosure policy for large-scale adverse events in an integrated health care system: identification of gaps and successes. *BMC Health Serv Res.* 2016;16(1):648.
4. Elwy AR, Wasan AD, Gillman AG, et al. Using formative evaluation methods to improve clinical implementation efforts: Description and an example. *Psychiatry Res.* 2020;283:112532.
5. Brownson RC, Jacobs JA, Tabak RG, Hoehner CM, Stamatakis KA. Designing for dissemination among public health researchers: findings from a national survey in the United States. *American journal of public health.* 2013;103(9):1693-1699.
6. Elwy AR, Bokhour BG, Maguire EM, et al. Improving healthcare systems' disclosures of large-scale adverse events: a Department of Veterans Affairs leadership, policymaker, research and stakeholder partnership. *J Gen Intern Med.* 2014;29 Suppl 4:895-903.
7. Schoenfeld AJ, Sturgeon DJ, Dimick JB, et al. Disparities in Rates of Surgical Intervention Among Racial and Ethnic Minorities in Medicare Accountable Care Organizations. *Ann Surg.* 2019;269(3):459-464.
8. Berenguer CM, Ochsner MG, Jr., Lord SA, Senkowski CK. Improving surgical site infections: using National Surgical Quality Improvement Program data to institute Surgical Care Improvement Project protocols in improving surgical outcomes. *J Am Coll Surg.* 2010;210(5):737-741, 741-733.
9. Bratzler DW, Houck PM, Richards C, et al. Use of antimicrobial prophylaxis for major surgery: baseline results from the National Surgical Infection Prevention Project. *Arch Surg.* 2005;140(2):174-182.
10. Carey K, Stefos T, Zhao S, Borzecki AM, Rosen AK. Excess Costs Attributable to Postoperative Complications. *Med Care Res Rev.* 2011.
11. Centers for Disease Control (CDC). 2019 Antimicrobial Resistance Report: Biggest Threats and Data. https://www.cdc.gov/drugresistance/biggest-threats.html?deliveryName=USCDC_426-DHQP-DM13106.
12. The Joint Commission. Surgical Care Improvement Project (SCIP) Specifications Manual Version 2010A1. https://manual.jointcommission.org/releases/archive/TJC2010B/SurgicalCareImprovementProject.html. Published 2010. Accessed.
13. Munday GS, Deveaux P, Roberts H, Fry DE, Polk HC. Impact of implementation of the Surgical Care Improvement Project and future strategies for improving quality in surgery. *Am J Surg.* 2014;208(5):835-840.
14. Stulberg JJ, Delaney CP, Neuhauser DV, Aron DC, Fu P, Koroukian SM. Adherence to Surgical Care Improvement Project Measures and the Association With Postoperative Infections. *JAMA.* 2010;303(24):2479-2485.
15. Department of Veterans Affairs Office of Inspector General. Healthcare Inspection Surgical Care Improvement Project , Report No. 07-00773-106. 2008.
16. Hawn MT, Vick CC, Richman J, et al. Surgical site infection prevention: time to move beyond the surgical care improvement program. *Ann Surg.* 2011;254(3):494-499; discussion 499-501.
17. Itani KM. Fifteen years of the National Surgical Quality Improvement Program in review. *Am J Surg.* 2009;198(5 Suppl):S9-S18.
18. Greenhalgh T, Robert G, Macfarlane F, Bate P, Kyriakidou O. Diffusion of innovations in service organizations: systematic review and recommendations. *Milbank Q.* 2004;82(4):581-629.
19. Branch-Elliman W, Pizer SD, Dasinger EA, et al. Facility type and surgical specialty are associated with suboptimal surgical antimicrobial prophylaxis practice patterns: a multi-center, retrospective cohort study. *Antimicrob Resist Infect Control.* 2019;8:49.
20. Mull HJ, Stolzmann K, Kalver E, et al. Novel methodology to measure pre-procedure antimicrobial prophylaxis: integrating text searches with structured data from the Veterans Health Administration's electronic medical record. *BMC Med Inform Decis Mak.* 2020;20(1):15.
21. Veterans Health Administration (VHA). *2018 National Surgery Office Annual Surgery Report* 2019.
22. Branch-Elliman W, O'Brien W, Strymish J, Itani K, Wyatt C, Gupta K. Association of Duration and Type of Surgical Prophylaxis With Antimicrobial-Associated Adverse Events. *JAMA surgery.* 2019.
23. Chambers DA, Glasgow RE, Stange KC. The dynamic sustainability framework: addressing the paradox of sustainment amid ongoing change. *Implementation Science.* 2013;8(1):117.

24. Puig-Asensio M, Perencevich EN, Livorsi DJ. Prolonged postprocedural antimicrobial use: A survey of the Society for Healthcare Epidemiology of America Research Network. *Infection Control & Hospital Epidemiology.* 2019;40(11):1281-1283.
25. Schonberger RB, Barash PG, Lagasse RS. The Surgical Care Improvement Project Antibiotic Guidelines: Should We Expect More Than Good Intentions? *Anesthesia and analgesia.* 2015;121(2):397-403.
26. Cataife G, Weinberg DA, Wong HH, Kahn KL. The effect of Surgical Care Improvement Project (SCIP) compliance on surgical site infections (SSI). *Med Care.* 2014;52(2 Suppl 1):S66-73.
27. Ingraham AM, Cohen ME, Bilimoria KY, et al. Association of surgical care improvement project infection-related process measure compliance with risk-adjusted outcomes: implications for quality measurement. *J Am Coll Surg.* 2010;211(6):705-714.
28. Hysong SJ, Teal CR, Khan MJ, Haidet P. Improving quality of care through improved audit and feedback. *Implementation Science.* 2012;7(1):45.
29. Department of Veterans Affairs. 2012 VHA Facility Quality and Safety Report. http://www.va.gov/health/HospitalReportCard.asp. Published October 2012. Accessed May 20, 2020.
30. Department of Veterans Affairs. 2010 VHA Facility Quality and Safety Report. http://www.va.gov/health/docs/HospitalReportCard2010.pdf. Published October 2010. Accessed May 20, 2020.
31. Asundi A, Stanislawski M, Mehta P, et al. Prolonged antimicrobial prophylaxis following cardiac device procedures increases preventable harm: insights from the VA CART program. *Infect Control Hosp Epidemiol.* 2018;39(9):1030-1036.
32. Branch-Elliman W, Stanislawski M, Strymish J, et al. Cardiac Electrophysiology Laboratories: A Potential Target for Antimicrobial Stewardship and Quality Improvement? *Infect Control Hosp Epidemiol.* 2016;37(9):1005-1011.
33. Puig-Asensio M, Perencevich EN, Livorsi DJ. Prolonged postprocedural antimicrobial use: A survey of the Society for Healthcare Epidemiology of America Research Network. *Infect Control Hosp Epidemiol.* 2019;40(11):1281-1283.
34. Agency for Healthcare Research and Quality (AHRQ). Health Care-Associated Infections. https://www.ahrq.gov/professionals/quality-patient-safety/patient-safety-resources/resources/hais/index.html. Published 2018. Updated 6/2018. Accessed September 20, 2019.
35. Lessa FC, Mu Y, Bamberg WM, et al. Burden of Clostridium difficile Infection in the United States. *New England Journal of Medicine.* 2015;372(9):825-834.
36. Feazel LM, Malhotra A, Perencevich EN, et al. Effect of antibiotic stewardship programmes on Clostridium difficile incidence: a systematic review and meta-analysis. *J Antimicrob Chemother.* 2014;69(7):1748-1754.
37. Linsenmeyer K, O'Brien W, Brecher SM, et al. Clostridium difficile Screening for Colonization During an Outbreak Setting. *Clin Infect Dis.* 2018;67(12):1912-1914.
38. Chawla LS. Acute kidney injury leading to chronic kidney disease and long-term outcomes of acute kidney injury: the best opportunity to mitigate acute kidney injury? In: *Controversies in Acute Kidney Injury.* Vol 174. Karger Publishers; 2011:182-190.
39. Chertow GM, Burdick E, Honour M, et al. Acute kidney injury, mortality, length of stay, and costs in hospitalized patients. *Journal of the American Society of Nephrology.* 2005;16(11):3365-3370.
40. Forster AJ, Andrade J, van Walraven C. Validation of a discharge summary term search method to detect adverse events. *Journal of the American Medical Informatics Association : JAMIA.* 2005;12(2):200-206.
41. Gibbs J, Clark K, Khuri S, Henderson W, Hur K, Daley J. Validating risk-adjusted surgical outcomes: chart review of process of care. *Int J Qual Health Care.* 2001;13(3):187-196.
42. Musy SN, Ausserhofer D, Schwendimann R, et al. Trigger Tool-Based Automated Adverse Event Detection in Electronic Health Records: Systematic Review. *J Med Internet Res.* 2018;20(5):e198.
43. Bates DW, Evans RS, Murff H, Stetson PD, Pizziferri L, Hripcsak G. Detecting adverse events using information technology. *Journal of the American Medical Informatics Association : JAMIA.* 2003;10(2):115-128.
44. Ferranti J, Horvath MM, Cozart H, et al. Reevaluating the safety profile of pediatrics: a comparison of computerized adverse drug event surveillance and voluntary reporting in the pediatric environment. *Pediatrics.* 2008;121(5):e1201-1207.
45. Murff HJ, Patel VL, Hripcsak G, Bates DW. Detecting adverse events for patient safety research: a review of current methodologies. *J Biomed Inform.* 2003;36(1-2):131-143.
46. Chambers DA, Glasgow RE, Stange KC. The dynamic sustainability framework: addressing the paradox of sustainment amid ongoing change. *Implement Sci.* 2013;8:117.

47. Rabin BA, Brownson RC, Haire-Joshu D, Kreuter MW, Weaver NL. A glossary for dissemination and implementation research in health. *Journal of public health management and practice : JPHMP.* 2008;14(2):117-123.
48. Proctor E, Luke D, Calhoun A, et al. Sustainability of evidence-based healthcare: research agenda, methodological advances, and infrastructure support. *Implementation science : IS.* 2015;10:88-88.
49. Moore JE, Mascarenhas A, Bain J, Straus SE. Developing a comprehensive definition of sustainability. *Implementation Science.* 2017;12(1):110.
50. Agency for Healthcare Research and Quality (AHRQ). About Learning Health Systems. https://www.ahrq.gov/learning-health-systems/about.html. Published 2019. Updated May 2019.
51. Kilbourne AM, Neumann MS, Pincus HA, et al. Implementing evidence-based interventions in health care: application of the replicating effective programs framework. *Implement Sci.* 2007;2:42.
52. Rogers EM. *Diffusion of innovations Vol. 5.* New York: Free Press; 2003.
53. Institute of Medicine (IOM). The Learning Health System Series. http://www.nap.edu/catalog/13301/the-learning-health-system-series. Published 2015. Accessed Nov 20, 2019.
54. Powell BJ, Waltz TJ, Chinman MJ, et al. A refined compilation of implementation strategies: results from the Expert Recommendations for Implementing Change (ERIC) project. *Implementation science : IS.* 2015;10:21-21.
55. Proctor EK, Powell BJ, McMillen JC. Implementation strategies: recommendations for specifying and reporting. *Implement Sci.* 2013;8:139.
56. Kelly AA, Jones MM, Echevarria KL, et al. A report of the efforts of the Veterans Health Administration national antimicrobial stewardship initiative. *infection control & hospital epidemiology.* 2017;38(5):513-520.
57. White House. National action plan for combating antibiotic-resistant bacteria. *Washington, DC.* 2015.
58. House Committee on Veterans Affairs. THE VA MISSION ACT OF 2018 https://veterans.house.gov/uploadedfiles/va_mission_act_summary.pdf. Published 2018. Accessed.
59. VA Office of Research and Development (ORD). Health Services Research and Development Updated Research Priorities. https://www.hsrd.research.va.gov/funding/PriorityDomains2019.pdf. Published 2019.
60. Department of Veterans Affairs. Program Announcement: Health Services Research and Development Updated Research Priorities https://www.hsrd.research.va.gov/funding/PriorityDomains2019.pdf.
61. VA Health Services Research and Development (HSRD). SERVE Toolkit for Veteran Engagement https://www.hsrd.research.va.gov/for_researchers/serve/default.cfm. Published 2020. Accessed May 20, 2020.
62. Asundi A, Stanislawski M, Mehta P, et al. Development and Validation of a Semi-Automated Surveillance Algorithm for Cardiac Device Infections: Insights from the VA CART program. *Sci Rep.* 2020;10(1):5276.
63. Dasinger EA, Branch-Elliman W, Pizer SD, et al. Association between postoperative opioid use and outpatient surgical adverse events. *Am J Surg.* 2019;217(4):605-612.
64. Linsenmeyer K, Branch-Elliman W, Kalver E, Mull HJ. Surgical site infections in outpatient surgeries: Less invasive procedures contribute substantially to the overall burden. *Infect Control Hosp Epidemiol.* 2019;40(10):1191-1193.
65. Mull HJ, Gellad ZF, Gupta RT, et al. Factors Associated With Emergency Department Visits and Hospital Admissions After Invasive Outpatient Procedures in the Veterans Health Administration. *JAMA surgery.* 2018;153(8):774-776.
66. Mull H, Stolzmann K, Schweizer M, Branch-Elliman W. Novel Methodology to Flag Cardiac Implantable Device Infections by Integrating Text Mining with Structured Data in the Veterans Health Administration's Electronic Medical Record. *JAMA Network Open.* 2020;in press.
67. Branch-Elliman W. A Roadmap for Reducing Cardiac Device Infections: a Review of Epidemiology, Pathogenesis, and Actionable Risk Factors to Guide the Development of an Infection Prevention Program for the Electrophysiology Laboratory. *Curr Infect Dis Rep.* 2017;19(10):34.
68. Branch-Elliman W, Strymish J, Itani KM, Gupta K. Using clinical variables to guide surgical site infection detection: a novel surveillance strategy. *American journal of infection control.* 2014;42(12):1291-1295.
69. Pindyck T, K. G, Itani K, et al. Validation of an Electronic Tool for Flagging Surgical Site Infections Based on Clinical Practice Patterns for Triaging Surveillance: Operational Successes and Barriers. *American journal of infection control.* 2017.
70. Mull HJ, Chen Q, Shwartz M, Itani KM, Rosen AK. Measuring Surgical Quality: Which Measure Should We Trust? *JAMA surgery.* 2014.
71. Mull HJ, Itani KMF, Pizer SD, et al. Development of an Adverse Event Surveillance Model for Outpatient Surgery in the Veterans Health Administration. *Health Serv Res.* 2018.

72. Mull HJ, Rosen AK, Charns MP, Itani KMF, Rivard PE. Identifying Risks and Opportunities in Outpatient Surgical Patient Safety: A Qualitative Analysis of Veterans Health Administration Staff Perceptions. *Journal of Patient Safety.* 2017;Publish Ahead of Print.
73. Branch-Elliman W, Ripollone JE, O'Brien WJ, et al. Risk of surgical site infection, acute kidney injury, and Clostridium difficile infection following antibiotic prophylaxis with vancomycin plus a beta-lactam versus either drug alone: A national propensity-score-adjusted retrospective cohort study. *PLoS Med.* 2017;14(7):e1002340.
74. Fetters MD, Curry LA, Creswell JW. Achieving integration in mixed methods designs-principles and practices. *Health Serv Res.* 2013;48(6 Pt 2):2134-2156.
75. Creswell JW, Plano Clark VL. *Designing and Conducting Mixed Methods Research.* Thousand Oaks, CA: Sage; 2011.
76. Hamilton AB, Finley EP. Reprint of: Qualitative methods in implementation research: An introduction. *Psychiatry Res.* 2020;283:112629.
77. VA Office of Information and Technology. VistAWeb Version 16.1.8.2 User Manual. Veterans Health Administration. https://www.va.gov/vdl/documents/Clinical/VistaWeb/vistawebusermanual.pdf. 2016.
78. Department of Veterans Affairs. VHA HANDBOOK 1102.01, National Surgery Office, Available at: http://www.va.gov/vhapublications/ViewPublication.asp?pub_ID=2861. 2013.
79. Asundi A, Stanislawski M, Mehta P, et al. Real-world effectiveness of infection prevention interventions for reducing procedure-related cardiac device infections: Insights from the veterans affairs clinical assessment reporting and tracking program. *Infect Control Hosp Epidemiol.* 2019;40(8):855-862.
80. Berrios-Torres SI, Umscheid CA, Bratzler DW, et al. Centers for Disease Control and Prevention Guideline for the Prevention of Surgical Site Infection, 2017. *JAMA surgery.* 2017;152(8):784-791.
81. Bratzler DW, Dellinger EP, Olsen KM, et al. Clinical practice guidelines for antimicrobial prophylaxis in surgery. *Am J Health Syst Pharm.* 2013;70(3):195-283.
82. Hamilton AB, Finley EP. Qualitative methods in implementation research: An introduction. *Psychiatry Res.* 2019;280:112516.
83. Hsieh HF, Shannon SE. Three approaches to qualitative content analysis. *Qual Health Res.* 2005;15(9):1277-1288.
84. Miles MB, Huberman AM. *Qualitative data analysis.* Thousand Oaks: Sage; 1994.
85. Elwy AR, Wasan AD, Gillman AG, et al. Using formative evaluation methods to improve clinical implementation efforts: Description and an example. *Psychiatry Res.* 2019:112532.
86. Birt L, Scott S, Cavers D, Campbell C, Walter F. Member Checking: A Tool to Enhance Trustworthiness or Merely a Nod to Validation? *Qual Health Res.* 2016;26(13):1802-1811.
87. Souden M, Breeling J, Atkins D. *Quality Safety Value EHRM Council – Research Workgroup and ORD Strategic Initiative for Research & EHR Synergy: Research and the EHRM: AMIA VA Breakfast Update.* Nov. 18, 2019.
88. Cerner. Cerner EHR Clinical Solutions. https://www.cerner.com/solutions/clinical-solutions. Published 2019. Accessed May 22, 2019.
89. Kreuter MW, Wang ML. From Evidence to Impact: Recommendations for a Dissemination Support System. *New Directions for Child and Adolescent Development.* 2015;2015(149):11-23.
90. Shin MH, Sullivan JL, Rosen AK, et al. Examining the validity of AHRQ's patient safety indicators (PSIs): is variation in PSI composite score related to hospital organizational factors? *Med Care Res Rev.* 2014;71(6):599-618.
91. Engle RL, Lopez ER, Gormley KE, Chan JA, Charns MP, Lukas CV. What roles do middle managers play in implementation of innovative practices? *Health Care Manage Rev.* 2017;42(1):14-27.
92. Engle RL, Tyler DA, Gormley KE, et al. Identifying barriers to culture change: A qualitative analysis of the obstacles to delivering resident-centered care. *Psychological services.* 2017;14(3):316-326.
93. Hawn MT, Gray SH, Vick CC, et al. Timely administration of prophylactic antibiotics for major surgical procedures. *J Am Coll Surg.* 2006;203(6):803-811.
94. Bronsert M, Singh AB, Henderson WG, Hammermeister K, Meguid RA, Colborn KL. Identification of postoperative complications using electronic health record data and machine learning. *Am J Surg.* 2019.
95. Colborn KL, Bronsert M, Hammermeister K, Henderson WG, Singh AB, Meguid RA. Identification of urinary tract infections using electronic health record data. *American journal of infection control.* 2019;47(4):371-375.

VA Boston Healthcare System (Boston, MA):

All research activities will occur at the VA Boston Healthcare System's (VABHS) Center for Healthcare Organization and Implementation Research (CHOIR), located at the Jamaica Plain campus in Boston, MA. CHOIR and VABHS have telework approvals in place for all members of the project team. Staff also have office space in VABHS. Co-Principal Investigator, **Dr. Hillary Mull** is located in Building 1 on the 11th floor where all project meetings will occur, and co-Principal Investigator **Dr. Westyn Branch-Elliman,** co-investigators **Marlena Shin** and **Ryann Engle**, project manager **Rebecca Lamkin**, programmer **Daniel Sturgeon** sit in Building 9. CHOIR is a VA HSR&D Center of Innovation (COIN) with the infrastructure, resources, and experience to support a broad range of research projects. CHOIR will be the site for all project meetings, and off-site investigators and team members will participate via Lync and/or VANTS conference lines. CHOIR occupies approximately 10,000 square feet of contiguous office and meeting space on the VA Boston campus. Both Drs. Mull and Branch-Elliman use this space, with direct access to the VA computing infrastructure. CHOIR investigators use file servers in the VABHS Research domain, managed by OI&T, as well as the VHA VINCI computational resources. All VA physical and information system security requirements are in compliance with VA standards, with maintaining data security as a high priority at CHOIR. All study data will be stored in data directories on a server behind the VA firewall with specific validated access only to project members. CHOIR has access to all software necessary to perform the research for this project, including SAS and SQL. Appropriate members of the project team have this software installed.

Other co-investigators and consultants are VA clinician researchers located in VA facilities around the country. They each have sufficient office space and equipment to support their project activities. Offsite co-investigators will participate in project meetings by phone to Lync and/or VANTS conference lines and will have secure access to shared storage folders on VINCI. These investigators include **Dr. Rani Elwy** (Bedford VAMC) and **Dr. Mary Hawn** (Palo Alto VAMC).

Dr. Kathyrn Colborn will obtain a Without Compensation appointment with the Denver VAMC upon project funding. She has an office on the University of Colorado campus in Aurora, CO. She will be working remotely and performing data analysis securely through VPN, in which all analysis and findings will reside on the VA system.

1. INTRODUCTION TO THE REVISED APPLICATION (Response to Reviewers)

Thank you to the team of reviewers for their very helpful and insightful feedback on this proposal and for highlighting the project's substantial strengths and potential to advance the field of implementation science and contribute to our understanding about the important question of how to sustain positive practice change. We appreciate your encouraging feedback in the summary points including: 1) the investigative team is well qualified with the prior collaboration and work in the relevant field; 2) this is a timely proposal on an important topic with support from the clinical program, and 3) the potential to contribute to implementation science and sustainability is significant. Based on their input, we revised the application to include a stronger focus on future implementation efforts (Aim III) and addressed other concerns. Specific responses to summary points and reviewer comments are included, below. In the revised application, changes are bracketed in the text, with the exception of Aim III, which is entirely new and thus not marked.

Responses to SMRB Committee Summary Discussion Comments:

Summary Point 4*. Some reviewers thought that methods and details of aims were not sufficiently developed (see Reviewers 1 and 2). R1: More explicit outcomes for Aim 3 would be helpful… I'm not a fan of Aim 3 — "Measure the scale and spread of pre and post-operative antimicrobial prophylaxis guideline compliance by surgical specialty to procedures not targeted by SCIP and in low complexity facilities". I feel that this should be explored as part of the qualitative analysis conducted in Aim 2 that seeks to identify facilitators and barriers of implementing the antimicrobial administration guidelines. R2: The hypotheses in Aim 3 do not achieve the stated goal. The outcome is not defined and the methods are vague… There are no metrics for "scale and spread" in Aim 3…. The hypotheses stated do not seem to assess the scale and spread of the guidance. Data will include only post-retirement data, comparing compliance for SCIP-included versus excluded procedures. This will not identify whether or not any difference in compliance was the result of SCIP. Associations with patient, procedure, and facility characteristics will also not indicate spread. A comparison using pre-test – post-test methods may answer these questions.*

We agree that it is critical to assess whether practice change and spread of best practices was or was not impacted by SCIP. In our revised application, based on reviewer input, we incorporated the following changes into the proposal, Section 6.3: 1) We will determine whether SCIP may have impacted antimicrobial practices in targeted procedures beyond the retirement of active reporting with a pre-post analysis in Sub-Aim 1.2; 2) We will assess the effect of SCIP spread by measuring differences in compliance with evidence-based antimicrobial between SCIP targeted and excluded procedures in Sub-Aim 1.3 (part of Aim III in the initial submission); 3) Finally, through qualitative interviews in Aim II, we will collect data on perceptions about how SCIP changed long-term practice, exploring the specific factors associated with ongoing compliance. This will include questions about whether and how SCIP impacted practice change in the eyes of surgical stakeholders (See updated interview guide, Appendix 6). Finally, we will triangulate the qualitative and quantitate data and use a mixed methods approach to determine factors that were associated with sustained practice change, including the role of SCIP (Section 6.3). We will then map these constructs to the Expert Recommendations for Implementing Change (ERIC) implementation strategies. This mapping will then be included in an implementation playbook (new Aim III) that will be used to guide a future implementation trial.

Our original Aim III hypotheses included that practices promoted by SCIP were more likely to be disseminated within facilities with active SCIP programs, however, based on reviewer feedback, this hypothesis is no longer evaluated in this proposal. Instead, we will focus on the other Aim III (now Sub-Aim 1.3) hypothesis: surgery-level compliance with SCIP best practices is impacted by whether a specific procedure was or was not included under the umbrella of the program. To address this question, we will apply the SCIP INF 1 and 3 algorithms to all surgeries performed in cardiac, orthopedic, general, gynecology and vascular specialties, both SCIP included and excluded, and evaluate whether practices spread within specialties. We will also examine other factors related to uptake of evidence-based antimicrobial practice within facilities that were part of SCIP, including temporal effects, facility factors, and patient characteristics.

Summary Point 5*. The next steps and implementation phase of this study are not explicit such as how to use the information collected to develop an intervention for low performing sites. R1: It would have greater impact on care if an intervention were to be developed and tested as part of the proposal. This project would be much better suited with a stronger translational component. For example, after identifying similar facilities with low compliance and identifying the barriers of implementation in the qualitative analysis, it would be really nice to synthesize the results into a low cost intervention to improve compliance and then apply the intervention to some facilities while keeping other similar facilities as controls so as to compare clinical outcomes such as rates of*

SSI. R1: This research ultimately seeks to provide guidance for implementing antimicrobial administration guidelines clinics of various structures and complexity. I feel that this science would be even more meaningful by translating the research conducted under Aims 1 & 2 into an intervention and then subsequently evaluated.

We agree that developing a proposal for future implementation and dissemination is critical for translating best practices into bedside clinical care. In response to reviewer feedback about the need to focus on translation of findings to improve patient safety at low-performing sites, we created a new Aim III, which will develop and refine an implementation playbook as a final deliverable (see Section 6.3). An implementation playbook is a toolkit and roadmap of key steps, best practices, resources, and implementation strategies. We will develop this tool for improving compliance with SCIP quality metrics.[1] As stated above, we will use results from Aims I and II to identify barriers and facilitators to sustainability of practice change and map these to ERIC implementation strategies. In Aim III, we will develop and refine additional information about the SCIP evidence base, advice for communicating with leaders, assessment tools, planning tools, training curricula, evaluation methods, and a plan for supporting ongoing measurement, all of which are necessary for future scaling-up and spreading of SCIP best practices. Following the completion of the proposed work, we will test the implementation playbook in a future HSR&D Hybrid Type III study. We will include measurement tools as a component of our playbook. For example, algorithms from Aim I will be included to help sites assess compliance rates with guideline-concordant antimicrobial use. These quality metrics will be paired with clinical outcomes data, for example, data from the VA Surgical Quality Improvement Program about rates of SSI. The implementation playbook will be presented to our newly proposed Operational Steering Committee (Section 2.0), which will be comprised of national and local VA surgical stakeholders, including representatives from the Pharmacy Benefits Management Antimicrobial Stewardship Office and the VHA Office of Reporting, Analytics, Performance, Improvement & Deployment (RAPID), the VA National Surgery Office (NSO) and Veteran representatives from Veteran Consultant Network, as well as surgeon leaders from the NSO's specialty-level Surgical Advisory Board.[2] The Operational Steering Committee will be asked to provide feedback about playbook elements, and the playbook will be updated and refined based on their input and recommendations.

Responses to All Other Comments (not covered above):

REVIEWER 2

R2.1: Aim 1.1: The informatics approach is well-designed and clearly described. Minor issues such as section references for INF-1 and INF-3 and statements that "prior to EPRP discontinuation" in 5.2 should be corrected.

Thank you, we have made these changes in Section 5.2 and Section 6.3, Sub-Aim 1.1.

R2.2: The Aim 1 outcome measure is not specified and the details in the analysis and power calculations are insufficient… Aim 1.2: Analysis section is at least confusing and possibly has conflicting statements. The data are said to be at the procedure level, as a repeated measure within facility. This indicates a 0/1 response. The test for coefficient significance is for a difference in slope between pre- and postretirement. This indicates some aggregation of the 0/1 response. The power calculation adjusts for intra-facility correlation and is targeting a difference in slope. There is a time aggregation but none is mentioned.

We appreciate the reviewer's concern and added additional information to Section 6.3. There is no time aggregation. The binary indicators for compliance for each procedure are the outcomes. Time will take on continuous values, likely in days, and an interaction between time and pre/post SCIP retirement, a binary factor, will allow us to estimate separate slopes for the time periods. To obtain linear trends through the data, we are aggregating the 0/1's between the pre-SCIP time points and post-SCIP time points separately, but we are not aggregating over the entire pre and post time periods because that would lose detail in the trends over time.

R2.3: The pre-SCIP retirement cohort includes only cases when SCIP compliance had reached a threshold. How was this determined and why is it important? I don't see that it was incorporated into the power calculation or how they will interpret the results given this exclusion.

In Section 6.3 Sub-Aim 1.2, we will assess voltage drop in a time-series analysis using 2011-2015 pre-SCIP retirement cases and 2016-2020 post-SCIP retirement cases. We are measuring whether compliance rates were lower after retirement, but our goal is not to assess change over time from the initiation of SCIP in 2005 through 2020. Therefore, we are limiting our pre-SCIP retirement cohort to include all reviewed cases starting in 2011 when average compliance rates leveled off around 90-95%. Approximately 5-10% of cases in the SCIP pre-retirement sample are non-compliant.

R2.4: The difference-in-difference model is specified but the data set described would not support use of such a

model; there are no data included that were not SCIP-eligible procedures.

Thank you for pointing this out. We clarified the analysis in Section 6.3, Sub-Aim 1.2.

R2.5: *What was the assumed slope (2011-2015) for the power calculation?*

We assumed between 2011-2015 the slope was relatively flat because compliance was stable. We have the power to detect a 5% difference in slopes between pre and post SCIP retirement (see Section 6.3, Sub-Aim 1.2).

R2.6: *Would a voltage drop be indicated by a change in slope or a change in intercept?*

We described the difference in slopes between pre/post SCIP and the immediate drop after 2015 as the voltage drop in our model. Indeed, the immediate drop is the change in intercept between slope pre and slope post.

R2.7: *The interview questions in Aim 2 may not provide the level of detail needed to identify facilitators and barriers… Aim 2: The questions in the draft interview are open-ended. I am not convinced that information needed to support your specific expected outcomes, identifying facilitators and barriers, will be provided without the addition of direct questions or a survey.*

We revised the interview guide so that it now consists of both structured and open-ended questions, which will identify facilitators and barriers (see Appendix 6 and description in Section 6.3, Aim II). We will use the Dynamic Sustainability Framework to guide our interview development and subsequent analyses of interview transcripts. Facilitators and barriers identified within these interviews will subsequently be mapped to ERIC implementation strategies, using a process that our team has developed over the course of many studies.[3,4] These strategies will be compiled into an implementation playbook (new Aim III).

R2.8: *The model specified is a binomial generalized linear mixed model but the outcome measure is not specified. The details of the power calculation are missing but state that 10% difference in the outcome measure can be detected. There are missing details in the power calculations so the adequacy of the data cannot be assessed.*

Our model tests the yes/no outcome of whether evidence-based antimicrobial practices were associated with each individual surgical procedure in our Sub-Aim 1.3 hypothesis test. We will calculate separate models for each of the two SCIP metrics and each of the five specialties. We revised the power calculation in Section 6.3 Sub-Aim 1.3 to provide additional details about our data and analyses.

R2.9: *The study aligns with VA goals and involves operational partners but the dissemination plan is lacks detail.*

We agree that a strong dissemination plan is important for integrating our findings into VA clinical care. We significantly expanded this section of our proposal (Section 7.0). Additionally, we also designed our study for dissemination from the outset by integrating an Operational Steering Committee, Section 2.0, and establishing a plan for face-to-face communication twice a year (e.g., via Skype) for the duration of the study.[5] We will also send our OSC brief reports (approximately 1 page) on study updates each quarter, a dissemination process used in our prior studies with significant operational engagement.[6]

R2.10: *The project seems feasible in the timeline and staffing with the exception of support personnel for the informatics work… Budget for the statistician is minimal: 15% for years 2 and 4 only.*

Mr. Daniel Sturgeon, an accomplished biostatistician, is our new analyst on the project team at 50% effort. Mr. Sturgeon has significant experience in informatics and statistical modeling and will work with Dr. Colborn.[7] Additionally, we updated our budget to include funding for Dr. Colborn for years 2-4 to increase her role.

REVIEWER 3:

R3.1: *It isn't immediately obvious why the algorithms developed and tested previously for non-SCIP procedures (p22-23) need to be substantially adapted and re-validated for application to SCIP procedures in sub-aim 1.1.*

SCIP metrics evaluate compliance with appropriate antimicrobial prophylaxis administration; best practices vary substantially between procedure types. For example, the bacteria that cause infections following cardiac surgeries (primarily Staphylococcal organisms, which live in the skin and respiratory tract) are different from the bacteria that cause infections following colorectal surgeries (primarily gram-negative organisms, such as E. coli, which live in the gastrointestinal tract). Because the bacteria causing the infections are different, antimicrobial prophylaxis regimens are different, necessitating tailoring of the algorithm to the types of antimicrobials documented in the medical record for each specialty. We clarified the rationale behind Sub-Aim 1.1, Section 6.3.

Title: Assessing the Sustainability of Compliance with Surgical Site Infection Prophylaxis After Discontinuation of Mandatory Active Reporting

Background: Surgical site infections (SSIs) are one of the most common types of healthcare-associated infections, accounting for substantial morbidity and mortality. Pre-incision antimicrobial prophylaxis is highly effective for reducing SSI; however, post-operative antimicrobials do not reduce SSI and can lead to increases in potentially fatal adverse events, such as acute kidney injuries and *C. difficile* infections.[8-10] Beyond the direct impact on the patient, excess antimicrobial use contributes to the burden of antimicrobial resistance, a critical healthcare threat and a major target of VA and non-VA initiatives.[11] In 2005, to increase compliance with SSI prevention measures, the VA implemented the Surgical Care Improvement Project (SCIP), which included a set of publicly reported evidenced-based antimicrobial guideline compliance metrics targeting high-risk surgeries in five specialties, such as cardiac bypasses and orthopedic total joint replacements.[12] The metrics required resource-intense manual review by a trained reviewer as part of the VA's External Peer Review Program (EPRP). Following implementation and active reporting, VA compliance with guideline-concordant pre-operative antimicrobial use (SCIP INF-1) and prompt discontinuation of antimicrobials postoperatively (SCIP INF-3) exceeded 95%.[13-15] After this level of compliance was achieved, SCIP was retired in 2015, as the measurement and reporting process was felt to be costly with limited additional expected benefit.[16,17] Since the program's retirement, no studies have examined if practice changes have been sustained. Greenhalgh *et al.* (2004) called sustainability of practice change "one of the most significant translational research problems of our time;" however, there remains a dearth of research about factors that influence sustainability.[18] **The proposed study addresses this gap by examining whether the positive changes in peri-operative antimicrobial use adopted during SCIP were *sustained* and how they *spread,* using the Dynamic Sustainability Framework.**

Prior Work: Preliminary findings by the two PIs demonstrate the need for research to evaluate the sustainability of guideline-concordant practices in surgeries targeted by SCIP, and the spread of these practices to the many surgeries that were not included under the umbrella of the program.[12] The PIs demonstrated that guideline-discordant pre- and post-operative antimicrobial use is common, particularly in outpatient surgery.[19,20] There were 190,000 VA surgeries in FY18 in the five SCIP specialties: cardiac, orthopedic, general, gynecology and vascular surgery;[21] of these, 64% were SCIP-excluded outpatient procedures.[12,19] The study PIs have also demonstrated that SCIP non-compliance results in substantial patient harms in the VA, including deaths from *C. difficile* infections directly caused by inappropriate and excessively prolonged antimicrobial exposure.[19,22]

Objectives: We will use the Dynamic Sustainability Framework (DSF) as our guide for the qualitative and quantitative aspects of the project, and to assess intervention, provider, facility, specialty and contextual factors associated with "voltage drop," which occurs when compliance with an intervention decreases with time.[1,23] The proposed research will address 1) the sustainability of practice changes associated with SCIP, 2) factors influencing sustainability and voltage drop, and 3) whether antimicrobial guideline compliance spread beyond the limited set of procedures targeted by SCIP. [Findings will be used to develop and refine an implementation playbook to inform a future VA HSR&D Hybrid Type III effectiveness-implementation study to support SCIP sustainability and spread.] Electronic algorithms to measure SCIP antimicrobial use will be adapted from methods developed in the PIs' prior work. These highly novel data mining algorithms leverage the rich VA EHR and capture structured data (e.g., pharmacy orders) and text data (e.g., documentation of antimicrobial administration in clinical notes) and represent a substantial technological advancement over costly manual chart review or incomplete electronic surveillance based on pharmacy data. We propose the following Specific Aims:

Aim I: Quantitatively measure sustainability [and spread] of antimicrobial prophylaxis guideline compliance.

Sub-Aim 1.1 Adapt existing informatics tools for clinical note text and antimicrobials unique to the surgical specialties targeted by SCIP. Use previously manually validated 2005-2015 SCIP/EPRP chart review data as the gold standard to assess criterion validity and to optimize algorithm performance.

Sub-Aim 1.2 Apply the final informatics tools to data from 2011-2020 and analyze change in compliance using interrupted time series analyses. Identify contextual factors associated with "voltage drop."

[Sub-Aim 1.3 Measure the spread within specialties of pre- and post-operative antimicrobial prophylaxis guideline compliance to procedures not targeted by SCIP.]

Aim II: Qualitatively assess SCIP sustainability in a sample of facilities through analysis of key stakeholder interview data. Identify facilitators and barriers to implementation sustainability according to DSF constructs, [and map these to Expert Recommendations for Implementing Change (ERIC) implementation strategies].

[Aim III: Develop an implementation playbook that will be tested in a future HSR&D Hybrid Type III trial, using input from an Operational Steering Committee comprised of national and local VA surgical stakeholders]

2a. Research Plan

1.0 Background

Surgical site infections (SSIs) are among the most common types of healthcare-associated infections, accounting for substantial morbidity and mortality. Evidence-based SSI prevention guidelines from the Centers for Disease Control and Prevention (CDC) and multi-society guidelines highlight the importance of antimicrobial prophylaxis for reducing SSI. These guidelines emphasize the importance of ***pre-incision*** antimicrobial prophylaxis for reducing SSI. However, post-operative antimicrobials do **not** reduce SSI and are associated with *increases* in other post-operative adverse events, such as acute kidney injuries and *C. difficile* infections.[8-10] Further, excess antimicrobial use contributes to the growing burden of antimicrobial resistance, a critical threat to the health and wellbeing of Veterans.

In order to increase compliance with SSI prevention measures to optimize peri-operative outcomes, the VA implemented the Surgical Care Improvement Project (SCIP) in 2005. The SCIP program included public reporting of compliance with evidence-based antimicrobial prophylaxis (i.e., appropriate pre-incision administration and discontinuation within 24 hours for non-cardiac surgeries, and 48 hours for cardiac surgeries). SCIP was developed by the Joint Commission and Centers for Medicare and Medicaid Services (CMS) and was broadly implemented, including in the VA. Tracking SCIP metrics required resource-intense manual review by a trained chart reviewer, which was completed as part of the VA's External Peer Review Program (EPRP). SCIP process measures were coupled with SSI measurement through the existing VA Surgical Quality Improvement Program (VASQIP). Following implementation and active reporting, compliance with guideline-concordant pre-operative antimicrobial use and prompt discontinuation of antimicrobials postoperatively exceeded 95%.[13-15] After this level of compliance was achieved, SCIP was retired in 2015, as the measurement and reporting process was felt to be costly with limited additional expected benefit.[16,17]

Prior HSR&D funded projects by the study's multiple PIs (Mull – CDA 13-270; Branch-Elliman – HSR&D I21 18-031) demonstrate the need for additional research to evaluate the **sustainability** of guideline-adoption among surgeries included in SCIP and the **spread** of guideline-based prophylaxis to procedures not targeted by the program. As part of their collaborative formative work, the PIs found that prolonged post-operative and post-procedural antimicrobial use is common, particularly in procedures and settings where SCIP was not applied, such as lower complexity facilities, outpatient surgeries, and surgical specialties not targeted by the program.

In FY18, there were 190,000 cardiac, orthopedic, general, gynecologic, and vascular surgeries performed within the national VA healthcare system. Of these, 64% were outpatient procedures not targeted by SCIP; in outpatient settings, the PIs found high rates of guideline discordant post-operative antimicrobial use.[19] Outside of the VA, less than 30% of facilities reported adoption of evidence-based antimicrobial prophylaxis guidelines.[24] The importance of early discontinuation of unnecessary antimicrobials during the early post-operative period is highlighted by additional work performed by the PIs, which demonstrated that guideline-discordant antimicrobial use results in substantial patient harms with each additional day of exposure, including deaths from *C. difficile* infections.[19,22] <u>The proposed research will yield important insights about how to sustain adoption of evidence-based antimicrobial practices after active policy and financial support for a program is discontinued</u>.

1.1 SSI Prophylaxis and the Evolution of SCIP

Following a 1999 report by the CDC on SSI incidence and prevention, the CDC joined with CMS to create the Surgical Infection Prevention (SIP) Project in 2002. This work evolved into SCIP in 2005 and included metrics related to antimicrobial prophylaxis (Table 1).[25] SCIP increased compliance with evidence-based guidelines and was associated with a decrease in SSI.[13,25-27] Of critical importance to the proposed studies, only a small subset of highly invasive surgical procedures within cardiac, orthopedic, general, gynecology and vascular specialties were included in SCIP (details are available in Appendix 3).[12] Although evidence based practices measured by SCIP INF-1 and 3 were recommended for nearly all surgical care, **less invasive surgeries were not covered**.

Table 1: SCIP Metrics and Targeted Procedures

Metric	Description	Specialty + Procedures
SCIP INF-1	Prophylactic antibiotic given ≤ 60 minutes before incision (120 minutes for vancomycin or fluoroquinolones)	<u>Cardiac</u>: bypass procedures, valve repairs, and repair of structural heart disease<br><u>Orthopedic</u>: joint (hip or knee) replacement<br><u>General</u>: colorectal surgery<br><u>Gynecology</u>: hysterectomy<br><u>Vascular</u>: peripheral and central bypass and shunting procedures, arterial and vein resection procedures, and endarterectomies
SCIP INF-3	Discontinuation of prophylactic antibiotic ≤ 24 hours after surgery (48 hrs for cardiac cases)	

VA compliance with SCIP metrics was high when the program was active and quality of care improved through the audit and feedback process conducted by EPRP. [28] In reports by the Office of the Inspector General, VA Facility and Quality Safety reports that average compliance rates with SCIP composite

metrics improved to greater than 95% by FY11.[29,30] Dr. Hawn et al., a co-investigator on the proposed study, published several papers on VA SCIP, finding that compliance rates for SCIP INF-1 averaged 93% while INF-3 averaged 84% from 2005-2010, with compliance increasing throughout the duration of the active program.[16]

1.2 Impact and Significance of Non-Compliance with Antimicrobial Guidelines

Data demonstrate that more than 30% of antimicrobial prescriptions are unnecessary[11] and that research into the best ways to implement antimicrobial stewardship strategies in surgical settings are urgently needed. As demonstrated by the study's PIs, **guideline-discordant post-operative use is high** in many surgical and procedural settings, including outpatient surgery and cardiac device procedures, underscoring the need for more research into how to improve peri-operative antimicrobial use.[19,31] Studies outside of the VA also demonstrate limited dissemination of clinical guidelines beyond the procedures covered by the SCIP INF measures, underscoring the scope of the challenge and the need for additional research in this area.[32,33]

Dr. Branch-Elliman's prior work demonstrated that unnecessary antimicrobial exposures lead to ***preventable*** harm, particularly *C. difficile* infections and acute kidney injuries (AKI), and contributes to the growing public health crisis of antimicrobial resistance.[11,34] *C. difficile* is the most common healthcare-associated infection in the US, causing more than 15,000 deaths each year.[11,35,36] Measurement of healthcare-associated *C. difficile* infections is one of the core hospital quality metrics monitored by the VA healthcare system. Given its importance to the rating of the hospital, identifying ways to reduce transmissions of *C. difficile* is of great interest to VA hospital directors and infection prevention personnel.[37] Recent data have found that healthcare-associated *C. difficile* infections may be particularly high in the VA surgical population,[37] underscoring the need for more research into how to improve peri-operative antimicrobial use. AKI is another antimicrobial-associated adverse event that contributes to excess post-operative length of stay and mortality and increases the risk of long-term end-stage kidney disease.[38,39] Beyond direct harms to the patient, unnecessary antimicrobial exposure is the major driver of **antimicrobial resistance, which the CDC has labeled an "urgent threat" to the health of the population.**[11] Highlighting the significance of Dr. Branch-Elliman's prior work on this topic, her paper was nominated as an HSR&D Best Paper for 2019 and VA HSR&D Director Dr. David Atkins recognized its importance at the 2019 HSR&D Meeting. The research in this proposal will address these critical health challenges and provide insights into how to ***sustain*** and ***spread*** strategies to optimize antimicrobial use in surgical settings and thereby improve Veteran and population health.

1.3 Transition from Manual Review to Electronic Surveillance of Quality Metrics

Surveillance and measurement of quality metrics has historically relied on time-intensive manual record review – the resource burden of SCIP was a major driver of the decision to retire the program. While manual record review is the gold standard in surveillance,[40,41] research is moving toward computerized tools to more efficiently scan electronic health record (EHR) data and measure quality and safety outcomes.[42] These electronically-augmented strategies may approach the accuracy of human chart review at a fraction of the cost, and have the potential to improve case ascertainment and data collection obtained through the manual review process.[43-45] There is potential to apply electronic surveillance algorithms to VA EHR data to measure compliance with SCIP INF-1 and 3, as further highlighted in Section 5, Prior Work and in Section 6, Methods.

1.4 Theoretical Basis and Frameworks

Sustainability is "the extent to which an evidence-based intervention can deliver its intended benefits over an extended period of time after external support from the donor agency is terminated."[46,47] Sustainability of practice change has been called "one of the most significant translational research problems of our time,"[48] however, despite the critical importance of the topic, in 2004 Greenhalgh *et al*. noted a "near absence" of studies that aim to answer questions about factors driving high and low levels of sustainability.[18] In the past 16 years, there has continued to be a dearth of research providing insights into this important topic.[49] The research in this study will aim to close this gap and is guided by Chambers *et al*. 2013 **Dynamic Sustainability Framework** (DSF, Figure 1). The DSF emphasizes the concept that ***sustainability*** is an ongoing aspect of implementation that requires continual adaptations and support. It highlights that successful sustainment of an intervention, such as evidence-based antimicrobial use practices, requires that the characteristics of the intervention be "consistently tracked, using valid, reliable, and relevant measures" and expects that the system will change and evolve overtime. Further, the framework underscores the importance of "ongoing assessment and quality improvement efforts" to "improve sustainment" and "identify opportunities for intervention improvement." In addition, the DSF highlights the importance of informatics advances to improve and enhance ongoing quality monitoring and improving efforts as part of a larger "**learning health system**" model.[46,50]

After discontinuation of active implementation support, the DSF highlights that programs can undergo ***voltage drop***. Voltage drop refers to the extent to which providers return to practices that were in place prior to an active

Fig. 1. Application of Dynamic Sustainability Framework[23]

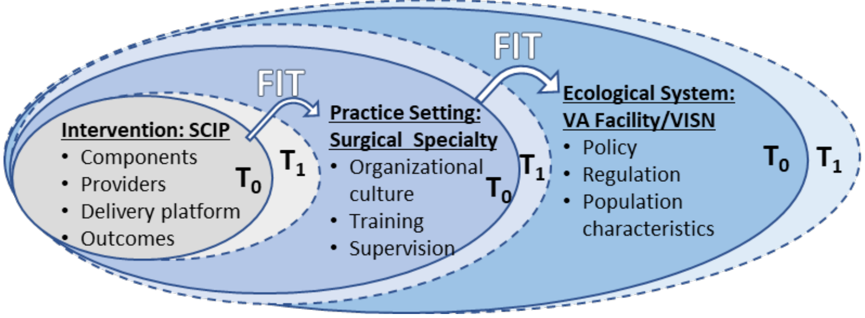

Figure adapted from Chambers 2013. T_0, T_1 = time pre/post SCIP

implementation strategy.[51] Figure 1 shows the main constructs of the DSF, including characteristics of the **intervention**, the **practice setting**, and the **ecological system**, and how they interact to impact sustainability over time. In the case of peri-operative antimicrobial use and the SCIP program, there are two ways in which voltage drop may occur: 1) Providers may stop appropriately administering pre-incision antimicrobial prophylaxis, i.e., non-compliance with SCIP INF-1, and 2) providers may inappropriately continue antimicrobials after skin closure i.e., non-compliance with SCIP INF-3. The deviation from the intervention in the years SCIP was active (T_0) to post-retirement (T_1) may be due to features of the intervention itself, specialty-specific practice setting issues, and/or policy or other facility-level changes that make up the ecological system, such as surgical or infection control local guidelines. In the context of optimizing surgical outcomes, voltage drop can lead to preventable harm. Reduction in pre-incision antimicrobials increases SSI, while inappropriately prolonged antimicrobials leads to an increase in adverse events, including resistance.

Beyond sustainability, there is also the potential that adoption of evidence-based practices *spread* to procedures not covered by SCIP. This is consistent with the **Diffusion of Innovation Theory**.[52] For example, the SCIP program targeted only a limited number of high-risk surgeries, such as coronary artery bypass grafting, cardiac valve replacements, and orthopedic total joint replacements. Other types of orthopedic procedures, such as arthroplasties and others, were not specifically targeted by all elements of the SCIP program. This raises the question about whether evidence-based antimicrobial practices may have diffused within specialties. In other words, once orthopedic surgeons adopted a practice change for hip and knee replacements, was it also adopted for less invasive procedures not specifically targeted by the program?

The research in this proposal addresses how and why voltage drop may occur, and the critical need for evaluations of the sustainability and spread of evidence-based practices. Our analysis will be guided by key factors highlighted in the DSF. In line with the DSF and the VA initiative to promote **Learning Healthcare Systems**,[53] the research in Aim I will adapt novel data extraction algorithms developed by the PIs (see Section 5.1 and 5.2) for measuring ongoing compliance with evidence-based antimicrobial use practices (SCIP INF-1 and 3) following the discontinuation of SCIP, and determine how uptake of evidence-based practices spread within specialties to procedures not included under the original program. In Aim II, qualitative interviews of stakeholders will identify factors impacting sustainability and spread by applying the DSF as an *a priori* coding framework in the analysis of these qualitative data. [We will then map identified barriers to the list of 73 Expert Recommendations for Implementing Change (ERIC) implementation strategies and will specify and operationalize these strategies to make them usable in a future trial.[54] In Aim III, we will create an implementation playbook.[55] Our implementation playbook will include lessons learned from Aims I and Aim II about factors associated with high and low SCIP compliance, and will include additional information on the SCIP evidence base, advice for communicating with leaders, assessment tools, planning tools, training curricula, evaluation methods, and a plan for supporting ongoing measurement with the tools developed in Aim I—all of these are needed for future scaling-up and spreading of the best practices promoted by the SCIP program. The playbook will be used to guide testing of implementation strategies in a subsequent Hybrid Type III effectiveness-implementation trial to improve peri-operative antimicrobial management].

2.0 VA Significance

Prior work demonstrates that, while sustainability is an essential aspect of implementation science, few studies have measured factors associated with sustainability, and how policy changes, such as the discontinuation of SCIP, impacted ongoing compliance with evidence-based practices. This study uses the **Dynamic Sustainability Framework** to address these significant questions and will yield important insights about how to achieve sustained adoption of evidence-based practices after the active program has ended. Informatics tools developed during the investigations will be adapted to measure compliance at the facility and specialty levels and will be included in an implementation playbook, laying the groundwork for a future VA HSR&D Hybrid Type III trial designed to sustain care improvements. Another major gap that will be addressed is an evaluation of how practice changes *spread* after an initial implementation directed at a small segment of clinical care. SCIP targeted major inpatient surgeries, but more minor procedures were not included in the

antimicrobial use metrics. The PIs' preliminary data suggests that there was differential spread of best practices; however, additional data are needed to determine factors associated with dissemination, or lack thereof. Lessons learned about factors driving the spread of practice change will provide critical insights for implementation science, and will be included in our implementation playbook, one of the final products of this study.

Operational Partners and Operational Steering Committee: [We designed our investigation for active dissemination throughout the study by involving, from the outset, an Operational Steering Committee (OSC), with whom we will work closely throughout the funding period.[5] Our OSC is comprised of enthusiastic operational partners at the Pharmacy Benefits Management Antimicrobial Stewardship Office, the VHA Office of Reporting, Analytics, Performance, Improvement & Deployment (RAPID) (See Letters of Support from Drs. Kelly Echevarria and Joe Francis). With our existing strong connections to the National Surgery Office (NSO) and the Women's Health Research Consortium, we will also seek their participation. Letters from them are not available due to HSR&D recommendations in the setting of the SARS-CoV-2 pandemic. Through our NSO connections, we will invite surgeon leaders from the NSO's specialty-level Surgical Advisory Board to participate.[2] Lastly, we will include a Veteran representative from Veteran Consultant Network (as described below). Face-to-face meetings via Skype will occur twice per year; brief, quarterly reports will be emailed to all members for information on study updates. All partners are committed to high-quality peri-operative care and compliance with evidence-based antimicrobial use metrics and have expressed interest in using the novel informatics tools that will be adapted and optimized during this study for real-time quality measurement. The OSC will be involved in validating each stage of our project work and confirming the potential utility data elements and proposed implementation strategies in our implementation playbook, which is the subject of Aim III.] The importance of the work is also highlighted by strong support from the Society for Healthcare Epidemiology (See Letter of Support, Dr. Hilary Babcock), which includes combating antimicrobial resistance through improved stewardship as one of its core missions.

Relevance to VA and HSR&D Priorities: This research complements legislative and research priorities of Veterans Affairs, the VA Office of Research and Development and HSR&D. Improving antimicrobial prescribing is a major goal of the VA,[56] the CDC, and the executive branch.[57] The national VA Antimicrobial Stewardship Directive (1031) states that, "antimicrobial resistance in hospitals and communities has risen and continues to rise at an alarming rate… antimicrobial stewardship programs are thought to be one of the most effective ways to improve antimicrobial utilization."[56] The VA MISSION Act identifies the goal of measuring safety, including complications from inappropriate antimicrobial use in surgery.[58] Our work directly contributes to two of the 2020 research priorities: **quality and safety** and **healthcare informatics**.[59] Additionally, the HSR&D Learning Healthcare System priority requests proposals that "take advantage of VA Electronic Health Records and other data and generates usable knowledge that guide continuous improvements and innovations in VHA healthcare system,"[60] which is the overarching mission of our proposed work in this IIR. We propose to use rich data only available within the VA EHR to assess compliance with evidence-based guidelines and determine the sustainability of a VA investment into active reporting. Lessons learned from our research will support continuous improvement in sustaining evidence-based care in VA surgery as part of a "learning healthcare system."

3.0 Innovation and Potential for Overlap

Our proposal presents an innovative approach to improving surgical care and antimicrobial stewardship. We propose to combine existing electronic data and health informatics tools (i.e., data mining through text queries and algorithm development/validation) to measure guideline compliance with surgical antimicrobial prophylaxis. Lessons learned about the sustainability and diffusion of evidence-based practices will have a direct impact on VA surgical care. Healthcare informatics tools adapted and optimized during the course of the investigations will lead to expansion of antimicrobial stewardship efforts to include a broad range of surgical types and specialties. [Our team will develop an implementation playbook (Aim III) that will be used in a future trial to test implementation strategies designed and specified to improve guideline uptake and long-term sustainability of improved practices]. In reviewing current and past research, ours is a unique proposal (see Appendix 4).

4.0 Patient Experience and Veteran Engagement

Veteran engagement is the cornerstone of patient-centered healthcare. Our HSR&D COIN, the Center for Healthcare Organization and Implementation Research (CHOIR), the primary site for the proposed research, has an active Veterans Engagement in Research Group (VERG). [CHOIR was one of the COINs involved in developing the Strengthening Excellence in Research through Veteran Engagement (SERVE) Toolkit to support VA Research Centers and investigators in efforts to include Veterans and other stakeholders in the development, implementation, and dissemination of research studies.[61] We will work with the VERG and invite members of its Veteran Consultant Network to learn about our project, share ideas on how to disseminate our findings, and provide feedback on our implementation playbook, described in Aim III.]

5.0 Research Team Qualifications and Prior Work

The project team has extensive and complementary healthcare informatics experience, clinical expertise, qualitative and implementation science expertise, and well-established collaborations to support the research in this proposal. [Please see Biosketches and Section 8.1 for additional details. The Multiple PIs have a strong working relationship,[19,20,62-66] and complementary expertise for completing the proposed projects.[22,67-73]] They also have extensive experience using the proposed databases, as outlined further in the Prior Work section. In addition, the team is strengthened by a strong team of collaborators and co-investigators, who provide expertise in surgery and surgical outcomes (Dr. Mary Hawn), qualitative methods (Ms. Marlena Shin and Ryann Engle), dissemination and implementation science (Ms. Shin and Dr. Rani Elwy), and advanced statistical methodology (Dr. Kathryn Colborn and [Mr. Daniel Sturgeon]).

5.1 Informatics Tool to Measure Pre-Procedure Antimicrobial Prophylaxis (SCIP INF-1)

The study PIs recently published the development and validation of an informatics tool to measure pre-procedure antimicrobial prophylaxis (SCIP INF-1) in cardiac device procedures. We propose to adapt this methodology, which combines structured data and SQL-based text queries, to measure administration of antimicrobial prophylaxis in surgical care.[20]

The PIs' highly novel methodology involved reviewing clinical prophylaxis guidelines and seeking input from clinicians knowledgeable about peri-procedural prophylaxis. Clinicians on the study team, guided by Dr. Branch-Elliman, developed a list of relevant antimicrobial names used for peri-procedural prophylaxis. Next, the team iteratively combined positive flags for an antimicrobial order or drug fill from structured data fields in the EHR, and hits on text string searches of antimicrobial names documented in electronic clinical notes, to optimize an algorithm to flag pre-incisional antimicrobial use with high sensitivity and specificity (see Appendix 5 for a detailed description of the EHR data sources and antimicrobial keywords). The algorithm was trained using an existing chart reviewed dataset and validated in a separate, national VA dataset. Discordant cases underwent expert manual review to identify reasons for algorithm misclassification and to identify barriers to implementation of the tool for real-time quality measurement, including facility-level variation.

Fig 2. Facility Variation in Pre-Incisional Prophylaxis (SCIP INF-1)

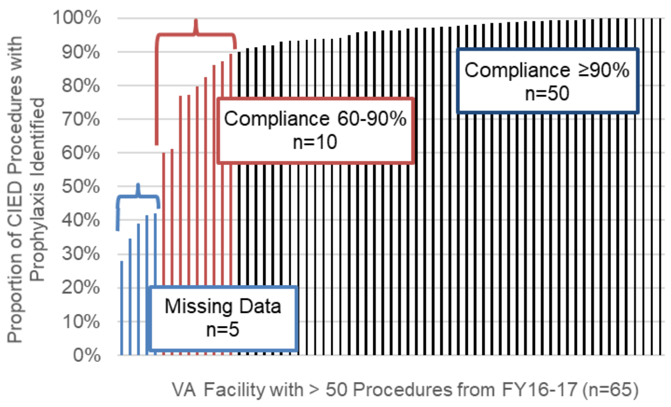

Of relevance to the proposed research, algorithm validation identified antimicrobial prophylaxis in 16,334 of 18,903 cardiac device procedures (87.8%) and found significant variation in pre-procedural antimicrobial use across facilities (Figure 2). Misclassification was evident in 5 facilities due to EHR documentation issues, such as notation of administration in hand-written notes only, documentation of "prophylaxis" but not which type, and vague information about antimicrobial administration timing. The algorithm identified 10 facilities where documentation appeared to be sufficient but compliance with recommended prophylaxis fell below 90%. In summary, the PIs' methodology has high accuracy in identifying guideline concordant use of antimicrobial prophylaxis prior cardiac device procedures, using data fields present in modern EHRs, including the VA.

5.2 Informatics Tool to Measure SCIP INF-3 and Spread of SCIP Practice to Outpatient Surgery

Drs. Branch-Elliman and Mull, the M-PIs, examined rates of prolonged antimicrobial use, SCIP INF-3, in outpatient surgery.[19] This study developed and tested the use of the SCIP INF-3 informatics algorithm to measure prescriptions for post-procedure antimicrobials. We measured the association between surgical specialty and SCIP INF-3 guideline compliance in VA outpatient surgeries from FY2016-17. General surgery and orthopedics procedures were targeted by SCIP in the inpatient setting; however, neither were evaluated in outpatient care. Data were analyzed using logistic regression with adjustments for patient and procedural factors. Among 153,097 outpatient surgeries, 7,712 (5.0%) received guideline-discordant antimicrobial prophylaxis lasting >24 hours after surgery. Rates of discordant antimicrobial prescribing ranged from 0.4% for eye surgeries, where systemic antimicrobials are ineffective, to 15-20% for urology procedures, which were not covered by SCIP INF-3. SCIP INF-3 compliance was ~3% among inguinal hernia repairs, a general surgery procedure, and 3.9% among outpatient orthopedic procedures. These preliminary findings suggest that compliance with SCIP metrics may have diffused *within* specialties but not *between* specialties; orthopedic and general surgery procedures not covered under SCIP appeared to have high rates of guideline compliance, whereas compliance was low in specialties that were not included in the program, such as urology.

6.0 Methods

This four-year proposal integrates quantitative and qualitative methods and will yield an implementation playbook to guide a future HSR&D IIR focused on dissemination and implementation of findings. The research in Aim I quantitatively measures compliance with antimicrobial prophylaxis guidelines to test the hypothesis that there has been voltage drop among SCIP-targeted procedures after SCIP discontinuation in 2015 (Sub-Aim 1.2) [and that there has been variable spread of best practices within different specialties (Sub-Aim 1.3).] To achieve the research in Aim I (see Figure 3), we will adapt two informatics tools previously developed by our team[19,20] and use 2005-2015 SCIP chart review data as the gold standard for assessing criterion validity (Sub-Aim 1.1). In Sub-Aim 1.2, we will apply the informatics tool to 2011-2020 data and analyze compliance rates with SCIP INF-1 and 3 over time using interrupted time series analyses. [In Sub-Aim 1.3, we will explore how SCIP practices spread within specialties by the validated SCIP INF-1 and 3 algorithms to procedures that were not targeted by the program.] Aim II will involve qualitative interviews with key stakeholders to identify factors affecting sustainability of evidence-based practice using the **Dynamic Sustainability Framework** (DSF) constructs. [We will then triangulate qualitative and quantitative findings to identify barriers and facilitators that were associated with high and low sustainability. These barriers and facilitators will then be mapped to the Expert Recommendations for Implementing Change (ERIC) implementation strategies,[54] using a validated process previously developed by our team.[4] Using a mixed methods approach in this study will provide us with rich data on factors affecting SCIP sustainability that we cannot obtain through quantitative methods alone.[74,75]

Fig. 3. Project Overview

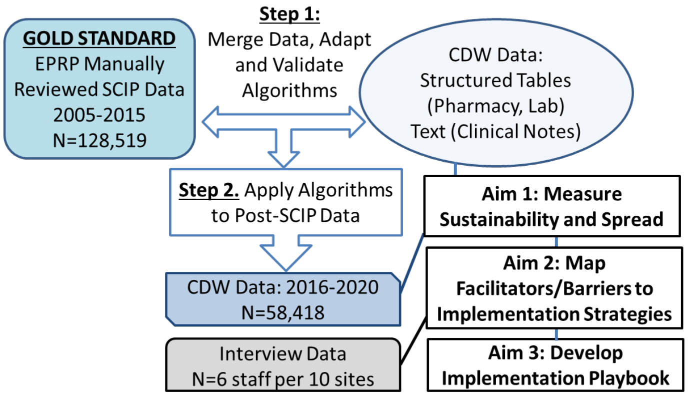

After data triangulation and mapping to the ERIC implementation strategies, we will develop and refine, based on stakeholder input, an implementation playbook (Aim III). It will consist of key components relevant to improving and sustaining compliance with SCIP metrics. The implementation playbook is essential for guiding a future HSR&D Hybrid Type III trial of a practice change intervention designed to support sustainability, scale-up and spread of SCIP throughout the VA healthcare system.] Figure 3 shows the flow of aims, steps and hypothesis over the course of the project. These aims and the associated hypotheses were developed based on strong prior work and preliminary data collected by the two PIs and the study team.

6.1 Study Population and Design

We will use VA data from FY2005 – 2020 [in surgically complex VA inpatient facilities (N=70)] to complete the study aims; this dataset will include inpatient and outpatient surgeries in cardiac, orthopedic, general, gynecology, and vascular specialties. The National Surgery Office (NSO) uses a clinician-defined set of Current Procedural Terminology (CPT) codes to classify procedures as surgical. EPRP-reviewed SCIP procedures will be identified using established methods (see Appendix 3). The volume of surgical procedures we anticipate based on prior work and the FY18 NSO Annual Report are presented Tables 2 and 3.[21]

For the qualitative aspects of the study, based on current guidance,[76] we propose to interview up to six key stakeholders in different VA hospitals for each of the five specialties, for a total of 60 interviews in ten VA facilities. Interview participants will have work duties related to SSI prevention and antimicrobial stewardship: surgical staff (including anesthesiologists), infectious diseases staff, pharmacists, and surgical nurses.

6.2 Data Sources and Access

Multiple VA data sets will be combined to get a comprehensive view of surgical utilization and antimicrobial prophylaxis practices. Prior to the project start date, we will apply for EPRP data from 2005-2015 for all surgeries that underwent SCIP INF-1 and 3 review. We will request visits, hospital stays, pharmacy and laboratory use and clinical notes for surgical care (e.g., operative notes, anesthesia records) performed in the five SCIP-targeted specialties from 2005-2020 from the CDW through VINCI. Per SCIP guidelines, only clean and clean-contaminated surgeries will be used in our study.[12] We will obtain antimicrobial use using the SAS statistical software coded programs established in Prior Work (Section 5; see Appendix 5 for algorithm details). As we have done in previous projects, we will request an extract of inpatient and outpatient VA utilization data on our cohort in the form of SQL tables and download these data into the Veterans Informatics and Computing Infrastructure (VINCI) workspace. We will also access laboratory data and medication orders from CDW. Facility identifiers (i.e., sta6a codes) will be used to differentiate locations of care and identify Ambulatory Surgical Centers (ASCs), as defined by the NSO. Lastly, we will use the VistAWeb/CAPRI national interface with the VA

EHR for chart review.[77] Both EPRP and VistAWeb identify patients by social security numbers (SSNs) and our CDW-derived data uses a primary key of 'PatientSID' to link patients across files; we will obtain permission to crosswalk PatientSID to SSN in order to match to EPRP and for those patients sampled for chart review.

6.3 Analysis Plan

<u>AIM I</u>: Quantitatively Measure Sustainability and Spread of Antimicrobial Prophylaxis Guideline Compliance

Sub-Aim 1.1: Adapt existing informatics tools for clinical note text and antimicrobials unique to the surgical specialties targeted by SCIP. Use previously manually validated 2005-2015 SCIP/EPRP chart review data as the gold standard to assess criterion validity and to optimize algorithm performance.

As presented in Sections 5.1 and 5.2, we developed and tested methods to identify compliance with SCIP INF-1 and INF-3; however, this work was applied to procedures and specialties not included in SCIP and thus requires adaptation and validation prior to application in SCIP-covered surgeries. Differences in the types of bacteria that cause SSI following different SCIP surgical types [(e.g., skin organisms following cardiac surgeries and gram negative and anaerobic infections that originate from a gastrointestinal source following colorectal procedures) lead to known variation in choice of antibiotic for prophylaxis. Thus, algorithms must be adapted to pull relevant antimicrobials for each surgical type.] In the first aim of the proposed study, we will adapt our informatics algorithms to inpatient surgical procedures targeted by SCIP (Table 1) and use manually reviewed data on SCIP INF-1 and 3 compliance from the EPRP to establish criterion validity.

Cohort: After obtaining EPRP data on SCIP compliance by facility and specialty, we will match SCIP-eligible procedures identified in the CDW from 2005-2015 to build a dataset for each specialty cohort with SCIP compliance information and structured/unstructured data relevant to our SCIP INF-1 and 3 algorithms (see Section 5). [The dataset will be limited to the SCIP-targeted complex surgical facilities (n=70).[78]] EPRP data contains all manually reviewed surgeries targeted by SCIP, and we expect our sample will include many non-compliant cases, particularly in the early period after program implementation.

Algorithm Development: We will follow the same process for adapting our previously developed electronic algorithms for SCIP INF-1 and INF-3, detailed in Appendix 5, and create separate algorithms for each of the five specialties covered by SCIP. For the purposes of this study, we will aggregate procedures within specialties (procedure codes associated with each specialty according to SCIP guidelines are included in Appendix 3). The data used in our algorithms are derived from the VA CDW, a single national data repository, which is where structured data and clinical notes within the VA are stored.[31,79] Data extracted from the VA CDW include structured tables with antimicrobial orders and documentation of antimicrobial dispensing/administration and also unstructured data, which include electronic clinical notes entered into the VA EHR. Of note, clinical notes that are not electronic (e.g., scanned documents in the EHR) are not available as searchable text data in CDW.

Four potential sources of antimicrobial prophylaxis documentation were identified and tested in previous work. The first was the computerized order entry system in the form of antimicrobial orders. These orders could be entered either in the outpatient setting prior to a planned procedure, or in the inpatient settings prior to an urgent or emergent procedure. The second was antimicrobial administration/dispensing, as documented in the VA bar coding system for inpatient orders, and outpatient prescriptions dispensed in the outpatient setting. The third was in clinical notes entered as text notes into the VA EHR and stored in the CDW; these could be any typed note signed within the system within the 7-day window prior to the procedure and including the procedure date. The fourth was scanned-in paper records, which include hand-written anesthesia records. The first three data sources will be considered for potential inclusion in the clinical informatics tool; no attempt will made to access the scanned-in paper records.

We will develop each algorithm to detect SCIP INF-1 and 3 antimicrobial prophylaxis iteratively over several stages, by varying 1) types of variables included in the tool (e.g., text note extraction only, orders only, administration only, or combinations of the three), 2) timing of the searches (e.g., including or excluding the procedure date), and 3) types of antimicrobials included in the tool, including an evaluation of only intravenous antimicrobials versus inclusion of oral medications. The clinicians on the study team will review the list of appropriate antimicrobials for surgical care as specified in the original SCIP guidelines and in current multi-society SSI prevention guidelines. [12,80,81] The list developed for the algorithms will include generic and brand antimicrobial names and variations to account for spelling errors. The list will be used to search clinical notes for documentation of administration pre- and/or postoperatively. The list will also be mapped to structured data in the VA EHR and orders and administration of relevant medications will be extracted. Informed by the PI's prior work, for structured variables, a range of a 0-7-day window prior to the procedure date will be used to measure INF-1. Antimicrobial orders lasting for >24 hours post-operatively (>48 hours for cardiac surgery) will be used to

measure INF-3.

The two algorithms will be applied to half of all EPRP reviewed surgeries targeted by SCIP from 2005-2015. Based on published work,[22] we estimate approximately 128,518 underwent manual review during this time period, leaving 64,259 gold-standard cases for algorithm development. Algorithm performance, i.e., criterion validity, will be assessed as sensitivity (how many true positive cases were identified as positive by the algorithm) and specificity (how many true negative cases were identified as negative by the algorithm). Manually reviewed EPRP data will be used as the gold standard for algorithm development. To finalize the algorithm, all discordant cases (e.g., algorithm flagged positive/EPRP manual review was negative, or algorithm flagged negative/EPRP manual review was positive) will undergo a second round of manual review by our research assistant with oversight by M-PI Dr. Branch-Elliman to identify the reasons for the discordant flag and to qualitatively classify the reason for discordance. These findings will be used to adjust and adapt the algorithms for each SCIP metric and each specialty procedure and VA facility to improve accuracy. We will conduct an analysis stratified by VA facility, to determine if facility-level effects, such as limited coding, impact algorithm performance and accuracy. In the PIs' published prior work, qualitative saturation was achieved after approximately 25 cases.[20]

Algorithm Validation: We will validate the final SCIP INF-1 and 3 algorithms by applying the structured and unstructured data extracts to SCIP-eligible procedures in the validation half of the 2005-2015 EPRP data. We will measure criterion validity: sensitivity, specificity and positive predictive validity for each algorithm per specialty (cardiac, orthopedics, general, gynecology and vascular surgeries).

Timeline: Based on prior work, we estimate Sub-Aim 1.1 will involve reviewing up to 125-250 charts for each SCIP measure to ensure accuracy and validity, for a total of 250-500 cases for each specialty. Analysis burden to program the algorithms for surgical care in the proposed study is expected to take 1 month per specialty-procedure for each SCIP metric. The structured data and antimicrobial list are established but will require modification. In prior work, chart review to confirm whether or not appropriate antimicrobial prophylaxis was performed ranged from 5-10 minutes, depending on procedure complexity and the number of clinical notes.[20] With a 50% programmer iteratively coding algorithms and full-time experienced research assistant doing chart review, we estimate adapting/developing the algorithms, reviewing discordant cases, applying final algorithms to validation data and assessing criterion validity with chart review for the five specialties will take ≈ 1 year.

Sub-Aim 1.2: Apply the final informatics tools to 2011-2020 data and analyze compliance rates over time using interrupted time series analyses.

We will separately assess SCIP INF-1 and 3 compliance by specialty using the final algorithms validated in Sub-Aim 1.1 and CDW data for eligible SCIP procedures. Changes in compliance rates will be measured with an interrupted time-series analysis with Dec. 31, 2015 as the final date for SCIP reporting in EPRP, using data five years before and five years after SCIP retirement (i.e., 2011-2020). This aim will test hypotheses related to SCIP voltage drop for each of the five specialties (see Figure 4):

H_1: Compliance with SCIP INF-1 Metrics, "Prophylactic Antibiotic Given Within 60 Minutes Before Incision" Has Dropped Since SCIP Ended

H_2: Compliance with SCIP INF-3 Metric, "Prompt Discontinuation of Prophylactic Antibiotic After Surgery" Has Dropped Since SCIP Ended

Fig. 4. Hypothetical Change in SCIP Compliance [15,16]

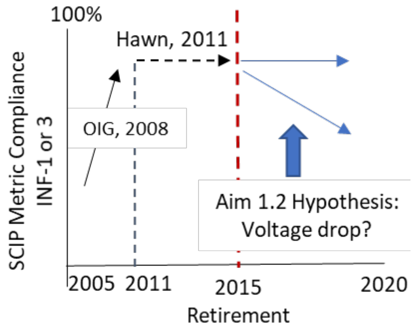

Cohort: Following the same process in Sub-Aim 1.1, we will identify SCIP-eligible procedures in complex surgical facilities from the CDW performed from 2016-2020 and merge the structured/unstructured data relevant to our SCIP INF-1 and 3 algorithms. The post-SCIP retirement cohort will then be merged with the SCIP-reviewed cohort from Sub-Aim 1.1; [however, because we want to evaluate the voltage drop, we will limit the SCIP-reviewed cohort to only those cases from 2011-2015 when SCIP compliance had already stabilized at a high threshold.]

Analysis: We will test the hypotheses that SCIP INF-1 and 3 compliance experienced voltage drop after SCIP retirement in 2015 and an overall change in rate over time using an interrupted time series model. Observations will be at the procedure level with facility repeated measures; thus, we will control for facility-level random effects. [The outcome measure will be a binary indicator of compliance for each procedure, and the models will include a binary indicator of pre- or post-SCIP retirement, a continuous variable for calendar time, and an interaction between these two variables. The beta coefficient for the interaction term will represent the difference in slopes between pre and post SCIP retirement, the coefficient for pre and post will indicate the "voltage drop", or immediate drop after the SCIP retirement, and the coefficient for time will indicate the slope for the pre-period. For each outcome, SCIP INF-1 and 3, and for each surgical specialty (Table 2), we will fit separate

models. To adjust for multiple comparisons, we will perform false discovery rate (FDR) correction on the betas for the interaction terms for each of the 10 comparisons. All analyses will be performed in SAS 9.4 or R software in the VINCI environment.

Table 2. Sample Estimates for Time Series Analysis

SCIP Eligible Specialties	SCIP Sample Estimate and Compliance 2011-2015			Post-SCIP Sample Estimate 2016-2020 Total Cases
	Total Cases	SCIP INF-1	SCIP INF-3	
Cardiac	16,489	100%	95%	16,489
Orthopedic	28,207	94.5%	83.1%	28,207
General	6,114	89.3%	83.4%	6,114
Gynecology	1,437	94.4%	91.3%	1,437
Vascular	6,171	91.7%	82.4%	6,171

Power Calculations: Table 2 shows the estimated number of SCIP-eligible surgeries performed during and after SCIP was retired in 2015. For each of the 10 comparisons, setting alpha at 0.01 to be conservative (given that we will adjust for multiple comparisons), assuming there will be correlation among patients from the same facilities (0.02), we can detect at least a 5% difference in slopes between pre and post SCIP retirement for each surgical specialty with greater than 90% power. This assumes approximately 95% SCIP compliance between 2011-2015 (Table 2). For 85% compliance, we need at least 3,807 cases pre- and post- to detect a 5% difference, which will still be achievable based on the estimated size of the cohort.]

Timeline: We anticipate the programming and analytic burden to build the cohort from 2016-2020, apply the two algorithms for each specialty, and merge with the 2011-2015 cohort will take 3 months beginning in year 2 of the study. Analyses will begin on a rolling basis when each specialty-level cohort with SCIP INF-1 and 3 data for 2011-2020 is complete. Therefore, we anticipate completing Sub-Aim 1.2 by the end of year 2.

Expected Outcomes: We hypothesize that providers have maintained high levels of compliance with SCIP INF-1 (pre-incision administration of antimicrobials) due to strong facility-level policies and procedures that were implemented during SCIP (e.g., surgical checklists, surgical stops, local protocols) to ensure compliance, but that rates of compliance with SCIP INF-3 have decreased over time, due to lack of surveillance with reporting and limited resources dedicated to maintaining the practice change, resulting in a "voltage drop."

[Sub-Aim 1.3 Measure the spread of pre- and post-operative antimicrobial prophylaxis guideline compliance within surgical specialties to procedures that were not targeted by SCIP.]

As described in the Diffusion of Innovation Theory, provider-level increases in adoption depend on awareness, persuasion, decision, implementation, and continuation.[52] SCIP active reporting demonstrably increased appropriate antimicrobial prophylaxis compliance in SCIP-targeted procedures, largely due to changes in provider behavior that may have been due to local policies designed to facilitate adoption. With respect to diffusion, the provider behavior change for one type of surgery may have led to changes for all procedures. For example, orthopedic surgeons may apply evidence-based practices measured by SCIP to all their orthopedic surgeries, including lower complexity outpatient procedures (see Figure 5). At the system level, the policies and practices may have changed for all surgical care and antimicrobial stewardship/infection control. For example, the Surgical Office or Infection Control may set policies or standards to effect change and we may see consistently high compliance across all specialties in the inpatient facility. These considerations lead to the following hypothesis:

H_3: Compliance with SCIP INF-1 and 3 Metrics Is Lower in SCIP-Excluded Procedures Compared to those Targeted by SCIP within Specialties

Fig. 5. Hypothetical Spread in Compliance with SCIP metrics

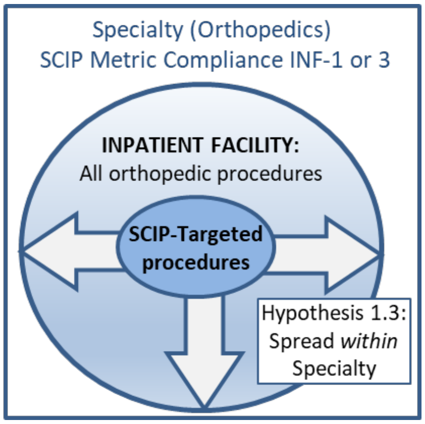

Cohort: We will identify all surgical procedures within the five specialties performed from 2016-2020 [in the 70 SCIP-targeted complex VA facilities] and merge the structured/unstructured data relevant to our SCIP INF-1 and 3 algorithms to build our dataset. [The cohort will differ from Sub-Aim 1.1 in that we will expand it to include all procedures, including outpatient procedures, performed within each of the five specialties.] Our analysis will be limited to clean or clean/contaminated surgeries and where pre-operative antibiotics are recommended.

Analysis: We will use binomial generalized linear mixed models to estimate the association between SCIP-targeted versus excluded procedures and the two SCIP metrics, adjusting for correlation among observations within the same facility using facility random effects. [The variance covariance matrix will allow us to estimate the correlation coefficients for nesting so that we can make inferences on the strength of these sources of correlation. Separate models will be fit for each outcome (SCIP INF-1 and 3) and surgical specialty; a total of 10 models. We will again apply FDR

correction on the p-values for the primary comparisons related to our hypothesis.

Power Calculations: Table 3 shows the proportion of VA surgeries within each of the SCIP targeted specialties in FY18 with the proportion of care done on an outpatient basis. There is variation in which specialties have a higher proportion of outpatient care – cardiac and vascular are primarily inpatient whereas general and gynecology are mostly outpatient-- consistent with non-VA findings. For each of the 10 models, we will have greater than 90% power to detect at least a 10% difference in the two SCIP compliance outcome measures between the comparison groups (see characteristics of interest in analysis section above) for each hypothesis. This assumes correlation of 0.02 at the facility level and alpha set at 0.01. For example, if compliance is 85% for females and 75% for males, we can detect this difference with an *effective* sample size of 938 individuals.]

Table 3. FY18 Surgical Volume by Setting

Specialty	Total	Inpatient	Outpatient
Cardiac	5,688	4,659 (82%)	1,029 (18%)
General	92,370	22,722 (27%)	69,648 (75%)
Gynecology	5,614	1,027 (18%)	4,587 (82%)
Orthopedic	56,115	21,722 (39%)	34,393 (61%)
Vascular	25,199	16,093 (64%)	9,106 (36%)

Timeline: We will build the cohort of all surgeries, inpatient and outpatient, within the five specialties in the first half of year 3. We will use the rest of year 3 to program models to test our hypothesis related to SCIP antimicrobial compliance spread within specialties.

Expected Outcomes: We hypothesize that we will find spread of SCIP practices within specialties to ineligible procedures, potentially due to changes at the provider level or local policy level (e.g., surgical checklists). We also expect system-level changes impact SCIP uptake within facilities.

<u>AIM II</u>: Qualitatively assess SCIP sustainability in a sample of facilities through analysis of key stakeholder interview data. [Identify facilitators and barriers to implementation sustainability according to DSF constructs, and map these to Expert Recommendations for Implementing Change (ERIC) implementation strategies.]

In Aim II, we will conduct interviews with key stakeholders to obtain qualitative information about factors that contributed to sustainability or lack thereof of SCIP antimicrobial use measures (SCIP INF-1 and 3) in SCIP-targeted procedures at a purposeful sample of complex VA facilities. We will analyze interview data to identify facilitators and barriers to implementation sustainability and map findings to DSF constructs.

Key Stakeholders: Key stakeholders for peri-operative antimicrobial use include members of the surgical staff (surgeons, anesthesiologists, nurses), infectious diseases staff (physicians and infection control/antimicrobial stewardship team members) and inpatient pharmacists. These are the providers who will be targeted at each of the participating VA facilities.

Selection of Sites and Recruitment: [We will purposefully recruit two facilities for each specialty (N=10 facilities) from the 70 high complexity VA facilities, targeting a range of sites, including regional diversity and urban, suburban, and rural variation.] We will use provider/staff data from the CDW to identify key stakeholders at each site. Among these, we will select two providers from each job category and link provider name from the CDW Staff table with the VA Global Address Book to obtain email addresses for key stakeholders. The identity of all employees in the cohort and in the recruitment sample will be kept private. We anticipate through this selection process that we will identify and recruit 60 stakeholders from 10 facilities (6 interview participants at each site). Our goal is to reach saturation within each of the five SCIP-targeted specialties across the sites; typical sample sizes for achieving this in implementation research range from 5 to 10 individuals in key roles.[82]

Recruitment: Operational partners will co-sign a letter outlining support for the project; the PIs will then send out this letter to a purposefully sampled selection of sites and inform them of the study prior to sending recruitment emails to providers. Thereafter, the project manager will send out recruitment emails to providers. We will use an opt-in approach. If we do not get enough stakeholders who agree to participate, then we will identify additional stakeholders to recruit using the approach described above.

Data Collection: Two co-investigators (Shin and Engle) will conduct 30-minute semi-structured telephone interviews with key stakeholders. All interviews will be digitally audio-recorded for transcription. Interviewers will follow a semi-structured interview guide, which consists of both structured and open-ended questions (Appendix 6). The interview guide will be revised and piloted prior to data collection. [Additionally, we will obtain input from our Operational Steering Committee (OSC) on the questions in the interview guide prior to data collection]. Our goal in refining and piloting the interview guide and in obtaining OSC input will be to ensure that we develop a set of questions that will allow us to collect rich information about facilitators and barriers of sustainability. Informed by our conceptual framework, the DSF, interviews will elicit information about the intervention (SCIP), the practice setting/context (surgical specialty), and the ecological system (VA facility/VISN). The overall purpose of the interviews will be to understand, from the perspectives of surgical staff, infectious diseases staff and inpatient pharmacists, what types of processes/practices have been implemented to help with peri-operative and

post-operative antimicrobial use and compliance; whether and how those processes/practices were adapted and sustained after SCIP was retired in 2015; whether and how those antimicrobial use and compliance processes/practices spread to other settings; and the facilitators that helped with and the barriers that hindered implementation, maintenance, and spread of those processes/practices. [In addition, we will obtain information about the contextual factors within the practice setting that affects implementation and sustainment of antimicrobial use and compliance by asking about the culture of the practice setting as well as trainings and resources that are available to help with antimicrobial use and compliance. Interviews will also ask questions to understand whether there are other metrics, policies, regulations or guidelines that are being used for peri-operative and post-operative antimicrobial compliance and how those have influenced processes/practices.]

Analysis: Coding of interview transcripts will be organized using NVivo, a qualitative analytic software. Transcripts will be initially coded using *a priori* constructs consistent with the DSF, our theoretical framework. We will use a directed content analysis approach with allowance for new themes to emerge.[83] New coding categories may be added or existing categories split or combined as more examples accumulate and similarities become apparent and code definitions are refined. In this way, the matrix of codes will eventually evolve to a point where the existing categories are sufficient to cover all new interview material being processed. As coding proceeds, new emergent themes will iteratively be identified, elaborated on and expanded based on team discussions, a process known as the constant comparative method. When no new concepts are discovered in the interview transcripts, saturation is said to have been achieved. Inter-rater reliability will be established using the "check-coding" process. Coders will independently code the same interview transcripts, and initial reliability estimates between all pairs of coders will be computed. Coders will then meet to compare their coding, discuss areas of difficulty, and reach agreement. A new interview will then be independently coded by all, and the process repeated until a stable level of agreement (>80%) is achieved across all coders. After coding is complete, we will summarize the data by producing site-specific descriptive summaries, which will include key information (quotes and themes) about our findings for each of the DSF constructs - the intervention (SCIP), the practice setting/context, and the ecological system. Within the site-specific summaries, we will note any differences in perspectives between key stakeholders at the facility as well as differences by specialty. The site summaries will result in a rich description of each DSF constructs and the factors (e.g., facilitators and barriers) that affect implementation sustainability of SCIP.

[Triangulation of Qualitative and Quantitative Data: When site-specific summaries are complete, we will triangulate our quantitative findings from Aim I and qualitative findings from Aim II. Utilizing Miles and Huberman's analytical approaches,[84] we will triangulate the data elements from the facilities and specialties and the compliance rates identified in Aim I with the qualitative findings from Aim II into a cross-site matrix. We will compare and contrast evidence to determine the key factors that may affect implementation sustainability of SCIP for sites with high or low SCIP compliance as well as for different specialties. We will then develop descriptive cross-site summaries based on our analysis of the integrated data from Aims I and II.

Mapping of Findings to ERIC Implementation Strategies: The data matrices will be used to map DSF-defined barriers and facilitators to the list of ERIC implementation strategies. Next, as shown in Figure 6, we will identify and specify implementation strategies to address relevant challenges from the evidence-based list developed by the Expert Recommendations for Implementation Change (ERIC) group.[23,55] Dr. A. Rani Elwy is an expert in mapping interview findings to theoretical constructs, including the DSF, and subsequently to implementation strategies and will lead this work.[4]]

[Figure 6. Process of Mapping Results to Implementation Strategies]

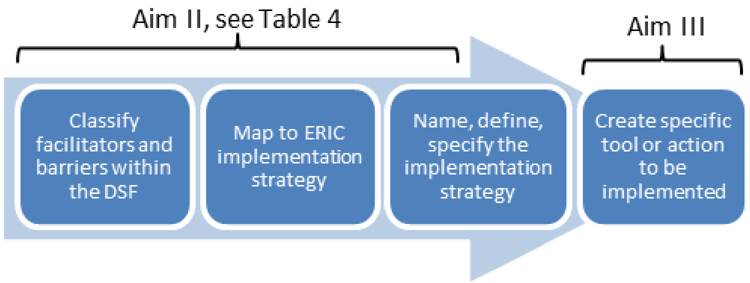

Timeline: Site identification and recruitment will begin in the last quarter of year 2 and we expect interview analysis to be completed by the end of year 3.

Expected Outcomes: We hypothesize that sustainment of practices is variable by facility and specialty, and that factors leading to ongoing high rates of compliance will include strong institutional support and local protocols and strong input from opinion leaders. We also anticipate finding that provider factors (practice location, training) contribute substantially to "buy-in" about the importance of antimicrobial stewardship practices and therefore ongoing compliance with SCIP metrics. [We also will identify barriers and facilitators to use that can be organized into an implementation playbook (Aim III) for future testing in a Hybrid Type III study. Table 4 provides examples of implementation barriers we may find, related to specific DSF constructs, and the selection and specification

of ERIC implementation strategies that may be identified as a result of our mapping process. While it is not possible currently to identify the exact barriers/facilitators or strategies that will emerge from Aims I and II, this table provides concrete examples of potential strategies that will be developed for our playbook (Aim III).]

Table 4: Process for Mapping Aim II Barriers to ERIC Implementation Strategies for Inclusion in Playbook

E.g. Aim II Formative Evaluation Barrier/ Facilitator	DSF construct	Example ERIC Implementation Strategy	Definition of Implementation Strategy	Example Tool or Action to be Included in Playbook
Lack of knowledge about pre-incision prophylaxis, and harms associated with prolonged prophylaxis	Providers involved in intervention (intervention)	Conduct educational meeting	Hold meetings targeted toward different stakeholder groups to teach them about the clinical innovation	Educational sessions to focus on developing these skills
Concern about "standard of care" and lack of resources to comply with established practices	Staffing, training (practice setting/context)	Conduct ongoing training;<br>Change clinic system	Plan for and conduct training in the innovation in an ongoing way;<br>Change clinic systems to allow better assessment of implementation or clinical outcomes	Development of webinar and in-person trainings, technology platform to provide new method of collaborating with surgical and infectious disease colleagues across sites
Concern about lack of national-level policy and surveillance program to detect infections	Population characteristics (ecological system)	Develop and distribute educational material	Develop and format manuals, toolkits, and other supporting materials in ways that make it easier for stakeholders to learn about the innovation	FAQ sheets for patients, family members, staff, clinicians prior to Hybrid Type III testing of implementation strategies for sustaining SCIP
Availability of Surgical Checklists, Protocols, and Order sets (facilitator)	Components (intervention)	Capture and share local knowledge	Capture local knowledge from sites on how clinicians made something work in their setting and share it with other sites	Example protocols that can be locally adapted and disseminated; Sharepoint site with resources for facilities

AIM III: Develop an implementation playbook that will be tested in a future HSR&D Hybrid Type III trial, using input from an Operational Steering Committee comprised of national and local VA surgical stakeholders.

The research in Aim III will yield an implementation playbook that will be tested in a future HSR&D Hybrid III study. Guided by the OSC, comprised of a team of key VA stakeholders, including our strong operational partners, surgical leaders from the NSO's Surgical Advisory Board, and Veteran representatives from the VERG, we will use results and our mixed methods dataset to identify the best strategies for intervention, whether at the facility or specialty-level, based on compliance rates with SCIP INF-1 and 3, and develop an implementation playbook to improve antimicrobial practices in VA surgical care.

Following analyses of Aims I and II, our team, guided by implementation expert Dr. Elwy,[3,85] will create an implementation playbook: a document that comprehensively describes how to sustain SCIP best practices in sites with low compliance on either of the SCIP antimicrobial use metrics; different strategies may be required depending upon whether sites have low sustainability with SCIP INF 1, 3, or both. The playbook will reflect the DSF in describing to sites the need to adapt SCIP antimicrobial use metrics, and the implementation strategies to fit local practice settings and the local environment and Veteran populations (ecological system). The implementation playbook will include the following sections : 1) evidence base for the SCIP metric; 2) advice on communicating with facility leadership and staff about SCIP to generate interest; 3) assessment tools for sites to gauge their readiness; 4) planning tools, such as timelines to ensure appropriate rollout of relevant interventions, 5) operational tools, such as example policies, checklists, and VA EMR order sets that can be locally adapted; 6) training curricula and educational materials for SCIP site champions to train other staff; 7) a plan for supporting ongoing measurement through local adaptation and calibration of the informatics tools developed in Aim I, and measurement of clinical outcomes, including SSIs, and other peri-operative adverse events. All of these elements are necessary to support future sustainment, scaling-up and spreading of SCIP.

We will seek feedback on the draft implementation playbook from our OSC and from site stakeholders using a member checking process[86] to ensure that the playbook has been developed as intended to increase the uptake and sustainability of SCIP antimicrobial use metrics at sites with low compliance with one or both of the INF metrics. Member checking, also known as participant or respondent validation, is a technique for exploring the credibility of research results. We will interview a subset of our respondents from Aim II (n=20) to assess the perceived acceptability and feasibility of elements of the draft implementation playbook, selected implementation

strategies, and locations for targeting the sustainability, spread and scale-up of SCIP. Using data collection and analysis methods described in Aim II, we will identify areas of the playbook that require further changes and updates. Our team will meet weekly to create these changes and will seek our OSC input on proposed changes, and their perspectives on the final implementation playbook.

Timeline: In the final year of the study, we will complete the research requirement to develop the implementation playbook. This will include interviewing 20 stakeholders and communicating with the OSC. In addition, we will collect and refine local policies and protocols to guide care that can be disseminated during the future Hybrid III study, and will develop general order sets that can be locally adapted by facilities with low-compliance, if this is found to be a potentially effective implementation strategy. We will also ensure that electronic algorithms developed in Aim I are appropriately calibrated for relevant VA sites.

Expected Outcomes: Our implementation playbook will be tested in a future HSR&D Hybrid Type III trial. This deliverable will establish the evidence base for SCIP, data elements, communication plans, planning, evaluation and measurement tools (e.g., SCIP INF 1 and 3 informatics tools developed in Aim I), protocols and order sets that can be locally adapted, and explicit intervention implementation strategies.

6.4 Limitations

We anticipate potential limitations of our study related to the Cerner EHR transition, missing EHR data, the COVID pandemic, and high levels of compliance in surgical specialties. A recent VA presentation at the American Medical Informatics Association meeting in November 2019 stated that Cerner will not impact EHR data collection until after 2020, which is after our data extraction has ended.[87] Thus, the Cerner transition will primarily impact the dissemination of our validated algorithm after study completion [the EHR transition has been further delayed due to the COVID pandemic; thus additionally mitigating this concern]. Surveillance algorithms will require updating after Cerner is fully implemented; however, importantly, much of our proposed work relies on structured data fields that will be populated in the Cerner EHR, and text searches are a strength of the Cerner system.[88] [EHR data missingness is resolved at all facilities, but if issues reappear, it may impact our strategy to develop and adapt the algorithms.] If this occurs, we will first conduct manual chart review to assess what data required in our algorithms are missing in the EHR (e.g., differential documentation of antimicrobial names - generic versus brand-names, documentation in paper-only records). If possible, coding will be adapted and adjusted to fully capture EHR data. If optimization is not possible due to insurmountable barriers (i.e., only documentation in hand-written notes), then these facilities will be excluded from the interrupted time series analysis; given the large number of procedures, this would not substantially impact study power. [The COVID pandemic may result in lower rates of surgical care in FY20. This is unlikely to affect study power, as we have a large sample size. We also anticipate that the COVID-19 outbreak will not impact the qualitative interviews, as these are conducted by telephone/Skype and thus will not be impacted by social distancing].

[Another potential limitation is that we may not find substantial variation and that all sites will have maintained outstanding compliance with SCIP metrics; based on our preliminary results, this is highly unlikely. If it does occur, we will proceed with the qualitative data collection in Aim II as planned and focus on factors that contributed to sustainability of the practice change; these data will be used to create an implementation playbook focused on the factors that facilitated sustainment of the practice change. Information about why providers did not revert back to outdated standards of care are important for advancing the field of implementation science about how to achieve and sustainability and promote similar improvements in other settings of care. Thus, we expect to still be able to collect rich and useful qualitative interview data, even if voltage drop has been minimal and/or if variation between sites is less than anticipated.]

7.0 Dissemination

[Dr. Elwy is leading the development and use of several dissemination support systems in other federally funded work and will provide input on the dissemination aspects of the proposal. Dissemination will proceed within and outside of the VA. Following the principles of Design for Dissemination,[5] we will engage our OSC throughout all study components with the goals of: 1) developing actionable deliverables that can have immediate impact on care delivery within the VA; and 2) building essential stakeholder engagement to enhance dissemination. To that end, the project team will update the OSC with our findings to inform VA policy and check in with them at regular bi-annual intervals (see Gantt chart), along with brief reports of study updates emailed on a quarterly basis. Our OSC will become part of our dissemination support system:[89] With their help, we will identify the systems needed to create a process for communicating with low compliance facilities about our implementation efforts; we will create processes for regular communication with VA and non-VA groups to share findings; and we will develop products for each targeted audience (clinical and operational leaders, front line providers, Veteran advocates) that will ensure that our messages about our activities to improve patient care are appropriate for each group with a stake in our efforts. Importantly, our stakeholders, through our regular

meetings, will serve as a "user review panel." This will ensure that our implementation playbook and the strategies we select for future testing in a Hybrid Type III trial are meaningful to the users who we intend to target in those efforts. We will also present at VA national meetings. Outside of the VA, dissemination will include formal presentations to AcademyHealth, the Annual Conference on the Science of Dissemination and Implementation in Health, and surgical or infection control specialty meetings. We will publish articles in the peer-reviewed literature (e.g., Implementation Science, Journal of American Medical Association), and we will make algorithm coding available through a resource sharing site, so that local facilities can use the algorithms to locally measure SCIP compliance].

8.0 Project Management Plan

8.1 Resources and Research Team

This project relies on the expertise of a distinguished group of researchers affiliated with the Center for Healthcare Organization and Implementation Research (CHOIR), an HSR&D Center of Innovation. CHOIR provides sufficient space, utilities and computer support to accomplish the project (see Facility and Other Resources). We are a team working in three sites with experience communicating through regular phone meetings, ad hoc phone calls and emails. The project team has diverse and complementary expertise that is ideally suited to complete the proposed project. The PIs will share leadership on the study as described in the Multiple PI Plan. Details of team roles are presented in the Budget Justification and in Biosketches. **Dr. Westyn Branch-Elliman**, MD, MMSc (PI) is an infectious diseases physician with expertise in epidemiology, surveillance, and the detection and management of infections, and implementation science.[22,32] **Dr. Hillary Mull**, PhD, MPP (PI) is an expert in clinical informatics, surgical adverse event detection, and surveillance.[70,71] She and Dr. Branch-Elliman developed methodology for reliably measuring antimicrobial prophylaxis directly relevant to this proposal.[19,20] **Dr. A. Rani Elwy**, PhD, (Co-I) is an expert in implementation science, dissemination, and qualitative methods.[4,6] She will oversee these aspects of the proposal by working with the qualitative team. **Marlena Shin**, JD, MPH, (Co-I) and **Ryann Engle**, MPH (Co-I) are experts in the application of qualitative methods for evaluating implementation studies.[90-92] They will lead the qualitative interviews and analysis in Aim II; they will also work closely with Dr. Elwy on Aim III study activities. Beyond CHOIR, **Dr. Mary Hawn,** MD, MPH, (Co-I) is a general surgeon at the Palo Alto VA and the Chief of Surgery at Stanford University. She has significant experience in health services research, including several studies of SCIP in VA based on EPRP data, and will advise on surgical processes of care and SCIP.[16,93] **Dr. Kathryn Colborn,** PhD, is an expert statistician.[94,95] She will oversee the statistical elements of the project and will provide guidance on hypothesis testing. [**Mr. Daniel Sturgeon**, our CHOIR project analyst, has significant experience in informatics and statistical modeling.[7]]

8.2 Schedule

The proposed project is scheduled over 4 years (see Table 5). Details for the timeline are in Section 6.

8.3 Patient Confidentiality and Data Security

This project will use individually identifiable patient data and interviews with protected subjects, VA employees. Standard patient and employee confidentiality and data security policies will be followed. See the Human Subjects Section for a detailed description of these policies and procedures.

Table 5. GANTT Chart

Project Aims	Specific Tasks (by fiscal year and quarter)	21			22				23				24				25
		2	3	4	1	2	3	4	1	2	3	4	1	2	3	4	1
Administrative	In JIT, obtain approvals from IRB, VINCI, and RAPID (EPRP data)																
Aim I	Merge 2005-2015 CDW and EPRP data to build specialty cohorts	x	x														
	Adapt SCIP INF-1 and 3 algorithms for specialties	x															
	Iteratively test/refine algorithms and measure criterion validity		x	x	x	x											
	Apply final algorithms to 2010-2020 data to measure compliance and test sustainability hypotheses by specialty					x	x	x	x								
	Sample all procedures within each specialty from 2016-2020 and apply algorithms. Conduct analyses to measure SCIP spread									x	x	x	x				
Aim II	Sample, recruit and interview key stakeholders								x	x	x	x					
	Map facilitators/barriers to ERIC implementation strategies												x				
Aim III	Develop implementation playbook and validate													x	x	x	x
Dissemination	Feedback to partners; conference presentations and manuscripts			x		x		x		x		x		x		x	
	Meet with Operational Steering Committee		x		x		x		x		x		x			x	

Human Subjects Research.

Risk to Subjects

I. Patient subjects:

The quantitative human subjects research in this IIR includes the use of medical record data of patients undergoing major surgical procedures covered by the Surgical Care Improvement Project (SCIP). Data that will be included in the study are available in existing VA electronic databases, and will include information about surgery type, timing, location, and provider, and also information about compliance with evidence-based surgical prophylaxis guidelines.

A. Patient Human Subjects Involvement and Characteristics:

a. *Inclusion Criteria*: This study is open to any VA patient who underwent cardiac, orthopedic, vascular, colorectal, or gynecologic surgery during the period from FY 2005-2020. Only surgeries that underwent review as part of the EPRP program will be included for the surgeries performed from FY 2005-2015; for the period from 2015-2020, any surgical procedure performed in any of these specialties may be included. Based on our preliminary results, we anticipate that this will be approximately 400,000 surgical procedures.

b. *Characteristics of Patients to Be Included*: Patients included in the study will be representative of local VA patient populations. No patients will be excluded on the basis of race, gender, or ethnicity. In line with VA research protocols, only patients 18 years or older will be included.

c. Patients Will Be Involved in the Following Ways: Electronic health records (EHR) of all adults who received a underwent a relevant surgical procedure within the national VA healthcare system during the study period (FY2005-2020) may be accessed. Data to be collected will include information about compliance with SCIP metrics from the External Peer Review Program (EPRP) and information collected as part of usual clinical care and documented in the national VA EHR. Specific data that will be excluded includes: demographic data, procedural data, antimicrobial use data, microbiology data, radiology data, ICD-10 codes/CPT codes, laboratory results, unstructured data from clinical notes and consult results. Data will be stored on a secure server with limited access and data will be encrypted. Data will be provided to facilities via a SharePoint site with encryption and limited access. No sensitive data (HIV results, drug use history, psychiatric history, etc.) will be collected or reported.

B. Sources of Materials:

No tissue samples or other biological specimens will be collected or stored.

C. Quantitative, Identifiable Data:

The only individually identifiable data that will be collected will be in the form of data or electronic health records. No specimens will be collected as part of this research. Identifiable information will be collected retrospectively and will be stored on a secure VA server with limited access and in compliance with VA privacy and security recommendations.

D. Potential Risks to Patient Subjects:

The research in this proposal will use the EHR to measure the sustainability and diffusion of peri-operative antimicrobial use after the discontinuation of the SCIP program. The major risk to patient subjects is breach of confidentiality, although surveillance activities are routine in healthcare settings and the risk in this study is no greater that the risk associated with standard surveillance practices. These practices include measurement of central line associated bloodstream infections and measurement of post-cardiac surgery infections, which occur as part of usual hospital operations and are reported internally and externally using secure data transfer strategies. To reduce the risk of breach of confidentiality, data will be stored on a secure server with limited access. No identifiable data will be stored on desktops, laptops, or removable storage devices.

E. Adequacy of Protection from Risk:

a. *Recruitment and Informed Consent*: Data on patients will be collected only from the EHR. A waiver of HIPAA authorization and waiver of informed consent will be requested from the IRB.

b. *Protection against Risk*: All research will be designed and conducted according to IRB and HIPAA regulations. Access to records will be limited to research staff. All paper records will be maintained in locked file cabinets within locked offices. Electronic data files will be encrypted/password protected on computers

maintained in a secure environment and will be transferred using secure, encrypted software with limited access, per VA security regulations.

F. Potential Benefits of Research to the Subjects and to Others

The research in this proposal has the potential to improve care in several ways although it will not impact subjects directly. First, the research will assess the sustainability of practice change after discontinuation of the active SCIP program. This will provide important information about how practice change occurs, and also about ongoing surgical quality within the VA healthcare system. Another benefit of the proposed projects is the development of a novel data mining system that will be locally adapted and can be applied for real-time quality monitoring. These novel tools can be used, if necessary, to re-in state and expand some of the reporting that was part of the SCIP program.

II. Provider subjects:

In addition to patient subjects, provider subjects at VA medical centers will also participate in the study through participation in qualitative interviews (Aims II and III).

A. The primary employee human subjects in this proposal are:

a) Surgical staff

b) Infectious diseases and infection control staff

c) Clinical infectious diseases pharmacists

B. Qualitative data collection (Semi-structured Interviews):

Qualitative data collection will in Aim II in the form of 30-minute telephone interviews with 60 participants from VA site. Stakeholders will be purposefully recruited from VA specialties from facilities that participated in SCIP with the goal of diversifying facility characteristics. Information collected in these interviews will be framed by the Dynamic Sustainability Framework and will focus on reasons for maintenance of practice change and reasons why providers may have chosen to revert to older standards of practice. In Aim III, 20 participants among the 60 that participated in the first round of interviews will be recruited to provide feedback on elements of an implementation playbook to increase and sustain compliance with guideline concordant antimicrobial use in surgical care. Interviews will be conducted over telephone and are expected to last 30 minutes. Participation in the interviews will be entirely voluntary, and providers will be informed they are welcome to withdraw their participation at any time. Other special subject classes, such as prisoners and institutionalized individuals will be excluded from this research. Female VA employee subjects may be pregnant during the course of the study, but their involvement in this research project will not affect a pregnancy.

C. Risk to provider subjects:

The major risk to provider subjects is breach of confidentiality and participation in the study. There is also the risk that providers may feel threatened by monitoring of clinical practice, however, quality measurement and monitoring is a standard feature in most procedural specialties.

D. Adequacy of Protection from Risk

The only individually identifiable data that will be collected will be in the form of data or records. These will include recordings of interviews and transcripts of interviews, procedural details, compliance with infection prevention interventions, and antimicrobial use. No specimens will be collected as part of this research. Identifiable information will be collected both retrospectively and prospectively and will be stored on a secure VA server with limited access.

The research in this proposal will be funded by external research funds but interviews will also take place in the context of clinical operations. As a result, if providers chose to participate, their identify and participation may be known by the Medical Director of Infection Prevention and/or the section chiefs of relevant divisions (Infectious Diseases, Pharmacy, Surgery), as well as possibly other supervisors and co-workers. Breach of confidentiality is a risk for provider subjects, who will be identified by name during approach and data collection, as there is a risk of disclosure of information about work place relationships or environment that could be perceived as sensitive, such as colleague’s attitudes about peri-operative infection prevention and antimicrobial stewardship, or perceptions of clinical leaders’ engagement or support. Stringent measures will be taken to protect provider subjects from these risks, and to inform provider that quality monitoring and

benchmarking reports are not punitive and will not be used to impact clinical privileges and compensation but are rather in place to ensure all Veterans receive the highest quality care.

Waiver of documentation of informed consent and a waiver of HIPAA authorization from the VA Central IRB will be requested for this subject population. Participating sites (VA employee subjects) will be recruited based on study site selection and referral. The recruiting statement will emphasize that the site and/or an individual provider may withdraw from the study at any time.

All research will be designed and conducted according to IRB and HIPAA regulations. Access to records will be limited to research staff. All paper records will be maintained in locked file cabinets within locked offices. Electronic data files will be encrypted/password protected on computers maintained in a secure environment, per VA security regulations. Every effort to keep information confidential will be made, however, no system for protecting confidentiality is completely secure in this operational context. An individual may choose to share with other the fact of his or her participation, or opinions about the in-service educational interventions. However, investigators will not disclose participation in the study or responses to any questions to anyone outside of the research team. Interviews will be conducted in-person or via the nation-wide VA teleconferencing system, at a time convenient to the participant. Audio recordings and interview notes will be saved directly to an access-controlled data folder on a secure server at the VA Boston Healthcare system.

The investigators will take stringent precautions to protect the confidentiality of subjects' personal information.

III. Importance of Knowledge to Be Gained.

The research in this proposal addresses an essential gap in implementation science assessing the sustainability and diffusion of evidence-based practices over time and after discontinuation of an active program. Lessons learned about factors that impact sustainability and diffusion will advance the field of implementation science and can be applied to improve care in other areas. Further, novel informatics tools developed during the course of investigations will be made widely available so that they can be used by local facilities to improve care in real-time. In addition, these tools are based on data mining tools that are readily adaptable to other electronic medical record systems (e.g. Cerner). Thus, coding developed and applied during this investigation is modifiable for implementation after implementation of the VA EHR modernization efforts. The implementation playbook will include strategies to improve and sustain compliance with evidence-based guidelines that will directly improve patient care. The study investigators and operational partners strongly believe that the benefits of this significant study far outweigh the risks involved, as the intervention has the potential to improve the quality of surgical care and will yield important insights into the sustainability of practice change.

IV. Data Safety & Monitoring Plan.

As described above, investigators have stringent data safety procedures in place to ensure subject protection. Breaches in confidentiality will be monitored and immediately reported to the IRB and the VA privacy officer.

V. Inclusion of Women & Minorities.

No subjects will be excluded from data collection or study participation on the basis of gender, race, or ethnicity. Women and minority subjects will be represented in study data to the extent that they are represented in the VA population and staff.

VI. Inclusion of Children.

In line with VA policies, children younger than 18 years of age will not be included.

Multiple Principal Investigator Plan

Drs. Mull and Branch-Elliman have complimentary expertise and a well-established research collaboration that make them ideally suited to lead this study as Multiple-PIs. They have several co-authored publications including work directly related to the proposed investigations and have previously collaborated on two HSR&D funded projects. Together, they will oversee all aspects of the proposed research plans.

Dr. Mull is a health services researcher and certified health informaticist with a strong background in clinical informatics, adverse event detection, quality measurement, and surgical research. Dr. Mull will serve as the corresponding PI and will be responsible for all communication with the VA, including submission of progress reports to the VA HSR&D service, Central IRB and data acquisition. Dr. Mull will be responsible for the informatics and analysis aspects of the proposal, including development and validation of the data mining algorithms and statistical analyses for hypothesis testing. Dr. Mull will also use her expertise in informatics and VA databases to direct the programmers on the project.

Dr. Branch-Elliman is a clinician with expertise in infectious diseases, infection prevention, and antimicrobial stewardship. Her research methodological expertise is complimentary to Dr. Mull's and includes clinical epidemiology, cost effectiveness and decision analysis, and implementation science. She has a long track record of publications in antimicrobial stewardship in surgical and procedural settings and will bring this expertise to the proposed study. Dr. Branch-Elliman will be responsible for with oversight of the diffusion and sustainability aspects of the proposal and will provide expert clinical input. Her specific role as PI will be to provide oversight into guideline-recommended practices, to correlate clinical guideline-based recommendations with data extracted by the mining tool, and to train research assistants in manual review of charts to ensure accuracy of data as well as to provide a gold-standard clinical review.

Drs. Mull and Branch-Elliman will co-lead weekly team meetings and also have one on one meetings once weekly to ensure the project progresses on schedule. They will collaborate on all dissemination efforts including publications and presentations.

Both Dr. Branch-Elliman and Mull are investigators at the same VA Center of Innovation (VA Boston CHOIR), somewhat mitigating the risk of conflict. However, if disagreement does occur the Multiple PIs have a systemic plan in place for conflict resolution. The first step will be to discuss the source of the disagreement with project Co-investigators (Drs. Hawn, Elwy, Shin, Engle and Colborn) to resolve the dispute with consensus from the study team. If this is not effective, then Drs. Branch-Elliman and Mull will arrange a meeting with Dr. Allen Gifford, MD, the Director of VA Boston CHOIR. During this meeting, the Multiple PIs will explain the source of the disagreement, and will agree that Dr. Gifford's third-party opinion/oversight will be respected and accepted.

Department of Veterans Affairs

Memorandum

Date: December 1st, 2019

From: Director, VA Boston, Healthcare System (523/151B)

Subj: Assessing the Sustainability of Compliance with Surgical Site Infection Prophylaxis After Discontinuation of Mandatory Active Reporting

To: David Atkins, MD, MPH, Director, HSR&D Service (124), VHA, Washington, D.C., 20420

1. I am pleased to endorse the submission of the proposal titled "Assessing the Sustainability of Compliance with Surgical Site Infection Prophylaxis After Discontinuation of Mandatory Active Reporting," by Westyn Branch-Elliman, MD and Hillary Mull, PhD, co-Principal Investigators.

2. Dr. Branch-Elliman and Dr. Mull's proposed Investigator Initiated Research (IIR) project presents an innovative approach to improving surgical care and antimicrobial stewardship. They propose to combine existing electronic data and health informatics tools (i.e., data mining through text queries and algorithm development/validation) to measure guideline compliance with surgical antimicrobial prophylaxis. Lessons learned about the sustainability and dissemination of evidence-based practices will have a direct impact on VA surgical care. Healthcare informatics tools developed during the course of the investigations will lead to expansion of antimicrobial stewardship efforts to low-complexity settings. Notably, prior work underpinning the proposed study was funded by an HSR&D Pilot Award (Dr. Branch-Elliman) and a Career Development Award (Dr. Mull).

3. Drs. Branch-Elliman and Mull are presently Research Health Scientists at VA Boston Healthcare System's Center for Healthcare Organization and Implementation Research (CHOIR), a VA HSR&D Center of Innovation (COIN). Both are 8/8ths VA employees. Dr. Branch-Elliman is also an infectious diseases consultant within the Department of Medicine at VA Boston Healthcare System. The research team on the proposed project includes Mary Hawn, MD, MPH from Palo Alto VAMC, A. Rani Elwy, PhD from VA Boston/Bedford CHOIR, Marlena Shin, JD, MPH from VA Boston CHOIR, Ryann Shin, MPH from VA Boston CHOIR and Kathryn Colborn, PhD from the University of Colorado; all provide important expertise that enchance the proposed project. The administrative and programming support, space, and resources necessary to complete the proposed research are in place at CHOIR to support this endeavor.

4. The Department of Veterans Affairs (VA) is undergoing significant policy and practice transformations including ensuring appropriate antibiotic stewardship and adherence to clinical guidelines. This study will close an important gap in dissemination and implementation science by assessing the sustainability of practice change after discontinuation of a mandated reporting program and measuring how practice changes may spread beyond the programs' goals. Tools developed during the investigations can be used by local VA facilities to promote uptake of evidence-based best-practice. Drs. Branch-Elliman and Mull and their associates on this project have my fullest support for this submission. We hope this application will be given favorable consideration.

Ng, Vincent

Digitally signed by Ng, Vincent
Date: 2019.11.15 14:27:35
-05'00'

VINCENT NG
Director, VA Boston Healthcare System

DEPARTMENT OF VETERANS AFFAIRS
Veterans Health Administration
Washington DC 20420

December 2, 2019

In Reply Refer To:

David Atkins, MD MPH
Director, HSR&D Service (10P9H)
Department of Veterans Affairs
810 Vermont Avenue, NW
Washington DC 20420

Dear Dr. Atkins:

I am writing to express my strong support and enthusiasm for the VA HSR&D IIR, *"Assessing the Sustainability of Compliance with Surgical Site Infection Prophylaxis After Discontinuation of Mandatory Active Reporting*" (Mull and Branch-Elliman, Co-PIs). The research in this proposal is highly innovative and will advance while answering important questions about the sustainability and spread of best practices after discontinuing mandated performance measurement.

The Surgical Care Improvement Project (SCIP) included several metrics of peri-operative prophylaxis, including appropriate administration and discontinuation of antimicrobials. The SCIP program was retired by CMS and VHA in 2015 after rates of compliance became "topped out" (VA compliance exceeded 97%). Since then, no studies, in VA or elsewhere, have addressed sustainment of these evidence-based practices. Further, SCIP was limited in scope and no data are available to determine whether practice changes adopted among surgeries covered under the program spread to surgeries that were not covered under SCIP.
Drs. Branch-Elliman and Mull have outstanding preliminary findings to support the premise of their project, which is to use novel data mining tools to measure compliance with surgical site infection prevention guidelines during the peri-operative period. They will adapt a data extraction tool that they developed for cardiac device procedures and to measure appropriateness of antimicrobials post-procedure. Their already-published findings suggest that there may be strong facility factors and specialty factors that impact adoption and sustainment of guideline-concordant care. The proposed research plan will more fully evaluate these questions and provide key insights into how we can improve antimicrobial use during the peri-operative period.

Sustainability of an intervention based on measurement and public reporting is of special interest to my office, which maintains the External Peer Review Program (EPRP) and played a critical role in collecting and interpreting peri-operative antimicrobial use data to drive performance improvement. I strongly support the proposed work as it will provide key insights into sustainability of performance measurement interventions in the VA and will promote our High Reliability Organization vision. Drs. Branch-Elliman and Mull and their team of expert scientists and clinicians are highly qualified to complete this research. My office will advise the study team on EPRP data and public reporting of SCIP metrics while the program was active in 2005-2015. We also look forward to helping direct analyses that answer critical questions about the sustainability and spread of best practices after retirement of performance metrics – this is

particularly salient given clamor from providers inside and outside VA about measure burden. Finally, given our interest in data-driven approaches to performance measurement, my office would be keenly interested in adopting any successful tools developed as a consequence of this partnered collaboration.

In summary, measurement and reporting of hospital performance to drive high-value care is a major target of VA operations. More research is needed to identify how to sustain performance measurement interventions like SCIP and this proposal addresses this important gap. It has my strongest recommendation and support. If you have any questions or would like more information about my role as an operational partner on this study, please feel free to reach out to me.

Sincerely,

Joseph Francis MD MPH
Chief Improvement and Analytics Officer
Veterans Health Administration

December 2, 2019

David Atkins, MD MPH
Director, HSR&D Service (10P9H)
Department of Veterans Affairs
810 Vermont Avenue, NW
Washington DC 20420

Dear Dr. Atkins:

I am writing to express my strong support and enthusiasm for the VA HSR&D IIR, *"Assessing the Sustainability of Compliance with Surgical Site Infection Prophylaxis After Discontinuation of Mandatory Active Reporting"* (Mull and Branch-Elliman, Co-PIs). The research in this proposal is highly innovative and will advance the field of antimicrobial stewardship while simultaneously answering important questions about the sustainability and dissemination of best antimicrobial use practices in procedural settings.

As you know, the Surgical Care Improvement Project (SCIP) included several important metrics related to peri-operative prophylaxis, including appropriate administration of antimicrobials pre-incision and appropriate discontinuation post-operatively. The SCIP program was retired in 2015 after rates of compliance within the VA exceeded 97%, and, since then, there have been no studies that address whether practice changes adopted during SCIP have been sustained. Further, SCIP was limited in scope and there are no data to answer the question of whether practice changes adopted among surgeries covered under the program spread to surgeries that were not covered under the program. Drs. Branch-Elliman and Mull have outstanding preliminary findings to support the premise of their project, which is to use novel data mining tools to measure compliance with surgical site infection prevention guidelines during the peri-operative period. They will adapt a data extraction tool that they developed for cardiac device procedures and to measure appropriateness of antimicrobials post-procedure. Their already-published findings suggest that there may be strong facility factors and specialty factors that impact adoption and sustainment of guideline-concordant care. The proposed research plan will more fully evaluate these questions and provide key insights into how we can improve antimicrobial use during the peri-operative period.

Antimicrobial resistance is a public health crisis. Antimicrobial overuse and misuse contribute to the development and spread of resistance; thus, there is a crucial need for research that sheds light on how to optimize antimicrobial use across the spectrum of care. The VHA Antimicrobial Stewardship Task Force (ASTF) is a multidisciplinary task force co-led by the VHA National Infectious Diseases Service and VHA Pharmacy Benefits Management, charged with developing, deploying and maintaining a national

level strategic plan for improvements in antimicrobial therapy management. We are happy to support this research in any way we can. The ASTF is committed to working with Drs. Branch-Elliman and Mull and their team of expert scientists and clinicians throughout the proposed project and any future QUERI partnered evaluations. Specifically, the ASTF will advise Dr. Mull and co-investigators on how to access relevant VA pharmacy data and medication guidelines, including for antimicrobial prophylaxis. Furthermore, I look forward to helping to direct analyses that answer critical questions about surgical care relevant to my position and policy goals. The ASTF will use Dr. Branch-Elliman and Mull's findings to tailor antimicrobial stewardship programs for surgical procedures, and use any insights generated to improve antimicrobial use practices in the VA and reduce unnecessary adverse events. Further, given our interest in informatics-based approaches to improve stewardship, the national office would be thrilled to use the tools you develop to track and improve peri-operative antimicrobial use throughout the national VA healthcare system.

In summary, antimicrobial resistance is a critical threat to the health of our population and thus is a major target of VA operations. More research is needed to identify how to improve antimicrobial use practices, and this proposal addresses this important gap. It has my strongest recommendation and support. If you have any questions or would like more information about my role as an operational partner on this study, please feel free to reach out to me.

Sincerely,

Kelly Echevarria, Pharm.D., BCPS, AQ-ID, BCIDP

National Clinical Pharmacy Program Manager, Infectious Diseases

VHA Pharmacy Benefits Management, Formulary Division

PBM designated Co-lead, VHA Antimicrobial Stewardship Task Force

SHEA
The Society for Healthcare Epidemiology of America

Board of Trustees 2019

President
Hilary Babcock, MD, MPH, FIDSA, FSHEA
Washington University School of Medicine

President-Elect
David Henderson, MD, FIDSA, FSHEA
Bethesda, Maryland

Vice President
Mary Hayden, MD, FIDSA, FSHEA
Rush University Medical Center

Secretary
Grace Lee, MD, MPH
Stanford University School of Medicine

Treasurer
A. Rekha Murthy, MD, FIDSA, FSHEA
Cedars-Sinai Health System

Past President
Keith Kaye, MD, MPH, FIDSA, FSHEA
University of Michigan Medical School

Councilors
Gonzalo Bearman, MD, MPH, FIDSA, FSHEA
Virginia Commonwealth University

Judith Guzman-Cottrill, DO, FSHEA
Oregon Health & Science University

Aaron Milstone, MD, MHS, FIDSA, FSHEA
Johns Hopkins University School of Medicine

Trevor Van Schooneveld, MD
University of Nebraska Medical Center

International Councilor
Dr. Mirian DalBen, MD, MSc
Hospital Sírio Libanês

Pediatric Infectious Diseases Society
Kristina Bryant, MD
University of Louisville

Community-Based Healthcare Epidemiologist Liaison
Scott Stienecker, MD, FIDSA, FSHEA
Parkview Health

December 4, 2019

David Atkins, MD, MPH
Director, HSR&D Service (10P9H)
Department of Veterans Affairs
810 Vermont Avenue, NW
Washington DC 20420

Re: Letter of Support, VA HSR&D IIR, "Assessing the Sustainability of Compliance with Surgical Site Infection Prophylaxis after Discontinuation of Mandatory Active Reporting"

Dear Dr. Atkins:

I am writing to express my great enthusiasm for Drs. Branch-Elliman and Mull and their VA HSR&D IIR, "Assessing the Sustainability of Compliance with Surgical Site Infection Prophylaxis after Discontinuation of Mandatory Active Reporting." The proposal is timely, innovative, and addresses a critical gap in research.

Surgical site infections (SSIs) are one of the most common types of healthcare-associated infections, accounting for significant morbidity and mortality in Veteran and non-Veteran populations. Evidence-based practices for reducing SSIs include appropriate pre-incision antimicrobial prophylaxis; however, post-operative antimicrobials do not reduce SSI and can lead to increases in other post-operative adverse events, such as acute kidney injuries and *C. difficile* infections. Further, excess antimicrobial use contributes to the burden of antimicrobial resistance, which is a CDC "critical threat."

In the setting of high rates of guideline non-compliance, the Surgical Care Improvement Project (SCIP) was implemented in 2005. A major aspect of SCIP was the inclusion of publicly reported measures to promote antimicrobial use best practices. The program was developed by the Joint Commission and applied to both VA and non-VA settings. The metrics required resource-intense manual review by a trained chart reviewer as part of the VA's External Peer Review Program (EPRP). Following implementation and active reporting, compliance with guideline-concordant pre-operative antimicrobial use and prompt discontinuation of antimicrobials postoperatively exceeded 95%. After this level of compliance was achieved, SCIP was retired in 2015. Since the program was discontinued, there have been no studies assessing important implementation outcomes related to the program: Were practice changes sustained? How did adoption of guideline-based practices disseminate within and between surgical specialties? The research in this application is designed to answer these questions and will provide important insights into methods to improve implementation of antimicrobial prophylaxis guidelines in surgical and procedural settings.

SHEA is a professional society founded in 1980. SHEA improves public health by establishing infection prevention measures and supporting antibiotic stewardship among healthcare providers. SHEA educates the next generation of practitioners, leads research studies, translates research into clinical practice, develops smart policy, promotes antibiotic stewardship, and advances the field of healthcare epidemiology. SHEA's Research Network (SRN) is a collaborative infrastructure of over 100 unique facilities worldwide working on research projects to advance the knowledge base in healthcare epidemiology and infection prevention. We are happy to work with our members to support research like yours, which aligns with our mission.

In summary, there is a clear need for research investigating the sustainability and dissemination of evidence-based infection prevention practices, such as guideline-concordant surgical prophylaxis. As the President of SHEA, I enthusiastically support this work and am happy to promote and facilitate participation in the research within the healthcare epidemiology, antimicrobial stewardship, and infection control communities. Please feel free to reach out to me with any additional questions.

Sincerely,

Hilary M. Babcock, MD, MPH, FSHEA, FIDSA
President, Society for Healthcare Epidemiology of America
Medical Director, BJC Infection Prevention and Epidemiology Consortium
Medical Director of Occupational Health (Infectious Diseases)
Barnes-Jewish and St. Louis Children's Hospitals
Professor of Medicine, Infectious Disease Division
Washington University School of Medicine

SHEA Headquarters 1300 Wilson Boulevard, Suite 300 • Arlington, VA 22209 • Phone: 703-684-1006 • Fax: 703-684-1009 • Email: info@shea-online.org

Department of Veterans Affairs
Office of Research & Development
Data Management and Access Plan (DMAP) Template
(Version: 7/29/16)

Please complete and include in your application for funding according to the Request for Applications instructions.

1. Name of Principal Investigator: Westyn Branch-Elliman, MD & Hillary Mull, PhD

2. Title of Proposal: Assessing the Sustainability of Compliance with Surgical Site Infection Prophylaxis After Discontinuation of Mandatory Active Reporting

3. Publication requirement (check box to acknowledge):

☑ **Publications** from this research will be made available to the public through the National Library of Medicine PubMed Central website within one year after the date of publication (guidance is provided on the ORD website).

4. Public access to **final data sets** resulting from the proposed research (check A or B below):

☐ A. Final data sets underlying all publications resulting from the proposed research will not be shared outside VA, except as required under the Freedom of Information Act (FOIA), for the following reasons:

500 character limit

If your response to Question 4 is A, STOP HERE.

☑ B. Final data sets underlying all publications resulting from the proposed research will be shared outside VA.

Mechanisms for public access to final data sets underlying publications from this research

5. Research **involving human subjects**:

☐ A. The data sets do not include research involving human subjects.

If your response to Question 5 is A, proceed to Question 6

☑ B. The data sets include research involving human subjects (check all boxes below that apply).

☐ Individually Identifiable Data will be shared pursuant to valid HIPAA Authorization, Informed Consent, and an appropriate written agreement limiting use of the data to the conditions as described in the authorization and consent.

☑ Individually Identifiable Data, excluding Veterans' names and 38 USC §7332-protected information, will be shared pursuant to a written request and IRB approved waiver of HIPAA authorization, with the approval of the Under Secretary for Health, in accordance with VHA Handbook 1605.1 §13.b(1)(b) or §13.b(1)(c) or superseding versions of that Handbook.

Note: Subject to all other listed requirements, Veterans' names may be shared with other Federal agencies (38 USC §5701), and with non-Federal investigators who provide the names and addresses of the individual subjects.

☐ Individually Identifiable Data, including 38 USC 7332-protected information, will be shared pursuant to the above requirements and a written assurance from the recipient that the information will be maintained in accordance with the security requirements of 38 CFR Part 1.466, or more stringent requirements, the information will not be re-disclosed except back to VA, and the information will not identify any individual patient in any report of the research or otherwise disclose patient identities.

☐ A Limited Dataset (LDS) will be created and shared pursuant to a Data Use Agreement (DUA) appropriately limiting use of the dataset and prohibiting the recipient from identifying or re-identifying (or taking steps to identify or re-identify) any individual whose data are included in the dataset.

☐ A de-identified, anonymized dataset will be created and shared.

NOTE: Where practicable, sharing should take place under a written agreement prohibiting the recipient from identifying or re-identifying (or taking steps to identify or re-identify) any individual whose data are included in the dataset. However, it is permissible for final datasets in machine-readable format to be submitted to and accessed from PubMed Central (and similar sites) provided that care is taken to ensure that the individuals cannot be re-identified using other publicly available information.

6. Research involving **animals and/or basic science**:

☑ A. The data sets do not include research involving animals and/or basic science.

If your response to Question 6 is A, proceed to question 7

☐ B. The data sets include research involving animals or basic science:

Final data sets underlying publications resulting from this research will be shared using the following mechanisms (*e.g., upon written request, through a website, databank, or repository):*

500 character limit

7. Description of how and where final data sets will be made available to the public:

Datasets meeting VA standards for disclosure to the public will be made available within 1 year of publication. Prior to distribution, a local privacy officer will certify that all datasets contains no PHI. Final data sets will be maintained locally until enterprise-level resources become available for long-term storage and access. Guidance on request and distribution processes will be provided by ORD. Those requesting data will be asked to sign a Letter of Agreement.

500 character limit

8. Explanation of how data sharing and preservation will enable validation of results by recipients:

The quantitative analytical datasets and statistical code used in the publication will be retained for 6 years, in accordance with VA record retention policy. The PI will maintain a crosswalk between the analytical dataset(s) and the public release datasets so that a VA-approved auditor or the PI could conduct or facilitate validation if needed.

500 character limit

9. Description of the mechanisms to ensure the protection of personal privacy of research subjects, the confidentiality of individually identifiable private information, and the secure maintenance of proprietary data and information (as relevant):

PI will create de-identified, study-specific datasets that include all variables in a publication. Some loss of information might occur given the need to remove PHI. The PI will replace social security and medical station numbers with study-specific numbers. The PI will drop date of birth and replace age with age categories, in accordance with PHI requirements for people 85 years of age and older.

500 character limit

Attachment 10 – Financial Disclosure Statement

No Disclosures.

Appendix Summary

Appendix 2: Abbreviations

Appendix 3: Summary of Procedure Codes Listed in SCIP Metrics

Appendix 4: Summary of Existing Studies

Appendix 5: Electronic Algorithms for Measurement of Antimicrobial Use in Cardiac Device Procedures

Appendix 6: Draft of Semi-Structured Telephone Interview Guide

Appendix 2: List of Abbreviations

AKI	Acute Kidney Injury
ASC	Ambulatory Surgical Center
CAPRI	Compensation and Pension Record Interchange
CART-EP	Clinical Assessment Reporting and Tracking- Electrophysiology
CDA	Career Development Award
CDC	U.S. Centers for Disease Control and Prevention
CDW	Corporate Data Warehouse
CHOIR	Center for Healthcare Organization and Implementation Research
CMS	Centers for Medicare and Medicaid Services
CPT	Current Procedural Terminology
DSF	Dynamic Sustainability Framework
EHR	Electronic Health Record
EPRP	External Peer Review Program
ERIC	Expert Recommendations for Implementing Change
FDR	False Discovery Rate
FY	Fiscal Year
HSR	Health Services Research
HSR&D	Health Services Research & Development
IIR	Investigator Initiated Research
INF	Infection
IRB	Institutional Review Board
JD	Doctor of Jurisprudence
MD	Medical Doctor
MISSION	Maintaining Internal Systems and Strengthening Integrated Outside Networks
MMSc	Master of Medical Sciences
MPH	Master of Public Health
MPP	Master of Public Policy
NSO	National Surgery Office
ORD	Office of Research and Development
OSC	Operational Steering Committee
PhD	Doctor of Philosophy
PI	Principal Investigator
PSI	Patient Safety Indicators
QUERI	Quality Enhancement Research Initiative
RAPID	Reporting, Analytics, Improvement and Deployment
SCIP	Surgical Care Improvement Project
SHEA	Society for Healthcare Epidemiology in America
SERVE	Strengthening Excellence in Research through Veteran Engagement
SIP	Surgical Infection Prevention
SQL	Sequel
SSI	Surgical Site Infection
SSN	Social Security Number
TIDIRH	Training Institute for Dissemination and Implementation Research in Health
VA	Veterans Affairs
VASQIP	VA Surgical Quality Improvement Program
VERG	Veterans Engagement in Research Group
VHA	Veterans Health Administration
VINCI	Veterans Informatics and Computing Infrastructure
VISN	Veterans Integrated Service Network

Appendix 3: Summary of Procedure Codes Listed in SCIP Metrics

ICD-9 CM CODE	SHORTENED DESCRIPTION
**CARDIAC**	
36.10	Aortocoronary bypass nos
36.11	Aortocor bypas-1 cor art
36.12	Aortocor bypas-2 cor art
36.13	Aortocor bypas-3 cor art
36.14	Aortcor bypas-4+ cor art
36.15	1 int mam-cor art bypass
36.16	2 int mam-cor art bypass
36.17	Abd-coron artery bypass
36.19	Hrt revas byps anas nec
35.10	Open valvuloplasty nos
35.11	Opn aortic valvuloplasty
35.12	Opn mitral valvuloplasty
35.13	Opn pulmon valvuloplasty
35.14	Opn tricus valvuloplasty
35.20	Opn/oth rep hrt vlv nos
35.21	Opn/oth rep aort vlv-tis
35.22	Opn/oth rep aortic valve
35.23	Opn/oth rep mtrl vlv-tis
35.24	Opn/oth rep mitral valve
35.25	Opn/oth rep pulm vlv-tis
35.26	Opn/oth repl pul valve
35.27	Opn/oth rep tcspd vlv-ts
35.28	Opn/oth repl tcspd valve
35.31	Papillary muscle ops
35.32	Chordae tendineae ops
35.33	Annuloplasty
35.34	Infundibulectomy
35.35	Trabecul carneae cord op
35.39	Tiss adj to valv ops nec
35.42	Create septal defect
35.50	Prosth rep hrt septa nos
35.51	Pros rep atrial def-opn
35.53	Pros rep ventric def-opn
35.54	Pros rep endocar cushion
35.60	Grft repair hrt sept nos
35.61	Graft repair atrial def
35.62	Graft repair ventric def
35.63	Grft rep endocar cushion
35.70	Heart septa repair nos
35.72	Ventr septa def rep nec
35.73	Endocar cushion rep nec
35.81	Tot repair tetral fallot
35.82	Total repair of tapvc
35.83	Tot rep truncus arterios
35.84	Tot cor transpos grt ves
35.91	Interat ven retrn transp
35.92	Conduit rt vent-pul art
35.93	Conduit left ventr-aorta
35.94	Conduit artium-pulm art

ICD-9 CM CODE	SHORTENED DESCRIPTION
35.98	Other heart septa ops
35.99	Other heart valve ops
**GENERAL/COLORECTAL**	
17.31	Lap mul seg res lg intes
17.32	Laparoscopic cecectomy
17.33	Lap right hemicolectomy
17.34	Lap res transverse colon
17.35	Lap left hemicolectomy
17.36	Lap sigmoidectomy
17.39	Lap pt ex lrg intest nec
45.00	Intestinal incision nos
45.03	Large bowel incision
45.49	Destruc lg bowel les nec
45.50	Intest seg isolat nos
45.71	Opn mul seg lg intes nec
45.72	Open cecectomy nec
45.73	Opn rt hemicolectomy nec
45.74	Opn transv colon res nec
45.75	Opn lft hemicolectmy nec
45.76	Open sigmoidectomy nec
45.79	Prt lg intes exc nec/nos
45.82	Op tot intr-abd colectmy
45.90	Intestinal anastom nos
45.92	Sm bowel-rect stump anas
45.93	Small-to-large bowel nec
45.94	Lg-to-lg bowel anastom
45.95	Anal anastomosis
46.04	Resect ext seg lg bowel
46.75	Suture lg bowel lacerat
46.76	Close lg bowel fistula
46.91	Myotomy of sigmoid colon
46.92	Myotomy of colon nec
46.94	Revise lg bowel anastom
48.50	Abdperneal res rectm nos
48.61	Transsac rectosigmoidect
48.62	Ant rect resect w colost
48.63	Anterior rect resect nec
48.64	Posterior rect resection
48.65	Duhamel rectal resection
48.69	Rectal resection nec
**ORTHOPEDICS**	
81.51	Total hip replacement
81.52	Partial hip replacement
81.54	Total knee replacement
**GYNECOLOGY**	
68.49	Total abd hyst nec/nos
68.69	Radical abd hyst nec/nos
68.51	Lap ast vag hysterectomy
68.59	Vag hysterectomy nec/nos
68.79	Radical vag hyst nec/nos
**VASCULAR**	
38.14	Endarterectomy of aorta
38.16	Abdominal endarterectomy

ICD-9 CM CODE	SHORTENED DESCRIPTION
38.34	Aorta resection & anast
38.36	Abd vessel resect/anast
38.37	Abd vein resect & anast
38.44	Resect abdm aorta w repl
38.48	Leg artery resec w repla
38.49	Leg vein resect w replac
38.64	Excision of aorta
39.25	Aorta-iliac-femor bypass
39.26	Intra-abdomin shunt nec
39.29	Vasc shunt & bypass nec

Appendix 4. Summary of Existing Studies

After a thorough review of the following data sources, HSR&D, QUERI, Clinical Trials, National Library of Medicine HSR Projects in Progress and NIH, we can safely conclude that ours is a unique study. No other studies explicitly address the sustainability of the Surgical Care Improvement Project or the scale and spread of peri-operative antimicrobial guideline compliance in relationship to an intervention targeted at select high-risk procedures.

Appendix 5: Electronic Algorithms for Measurement of Antimicrobial Use in Cardiac Device Procedures

We pulled data from many CDW tables (listed below) for our preliminary analyses. Included were tables from Inpatient, Outpatient, Laboratory, and Pharmacy.

1. Process Measures
    a. PRE-OP Antimicrobial utilization: Extract from TIU notes, Inpatient and Outpatient Pharmacy Dispensing, CPRS Orders
    b. POST OP Antimicrobial utilization: Extract from Inpatient and Outpatient Pharmacy Dispensing, CPRS Orders

2. Outcome measures:
    a. Cardiac device infection flags – Extract from: Lab Data (microbiology orders and results), Inpatient, Outpatient, and Inpatient Fee tables (for ICD10 Groups: SSI, General Infection codes, Infections associated with prosthetic material), Inpatient and Outpatient Pharmacy Orders (for antimicrobial orders and administration)
    b. Acute kidney injury measurement – Lab Data (pre-/post-procedural creatinine measurements)
    c. *C. difficile* infection measurement – Lab Data (PCR, EIA)

CDW Tables

BCMA_BCMADispensedDrug
Chem_PatientLabChem
CPRSOrder_OrderedItem
Dim.Date
Dim.DrugClass
Dim.LocalDrug
Dim.OrderableItem
ICD10
ICD10Diagnosis Version
Inpat_InpatientDiagnosis
Inpat_InpatientFeeDiagnosis
Micro_AntibioticSensitivity
Micro_BacteriologyReports
Micro_Microbiology
Outpat_VDiagnosis
Rxout_RxOutpatFill
SPatient_SPatient

Drug List

ANCEF
CEFACIDAL
CEFAMEZIN
CEFAZOLIN
CEFEPIME
CEFRINA
CEFTRIAXONE
CEFUROXIME
CILASTATIN/IMIPENEM
CIPROFLOXACIN
CLEOCIN
CLINDAMYCIN
DAPTOMYCIN
DICLOXICILLIN
DOXYCYCLINE
ELZOGRAM
ERTAPENEM
FAXILEN
GRAMAXIN
IMIPENEM
KEFOL
KEFZOL
KEFZOLAN
KEZOLIN
LEVOFLOXACIN
LINEZOLID
MEROPENEM
NAFCILLIN
NOVAPORIN
PIP/TAZO
PIPERACILLIN/TAZOBAC
TAM
PIPERICILLIN
PIPTAZO
REFLIN
TAZOBACTAM
VANCO
VANCOCIN
VANCOMYCIN
ZINOL
ZOLICEF
ZOSYN

Appendix 6: Draft of Semi-Structured Telephone Interview Guide

Note: In response to reviewer's comment, we have revised the interview guide to include structured questions. This draft interview guide now provides examples of the types of structured (with draft answer choices) and open-ended questions that we may ask during the interviews. For structured questions, we will ask interviewees to elaborate on and explain why they chose a specific answer; in addition, we will follow-up with questions while asking for examples and clarification as needed. For open-ended questions, we will probe responses and will ask for examples as well as ask for clarification as needed. Questions may be slightly adapted based on the position of the participant. We will refine the interview guide and will pilot it prior to data collection. Additionally, we will obtain input from our Operational Steering Committee on the questions in the interview guide prior to data collection. Our goal in refining and piloting the interview guide as well as obtaining input from our Operational Steering Committee will be to ensure that we develop a set of questions that will allow us to collect rich information about facilitators and barriers.

<table>
<tr><th colspan="2">Dynamic Sustainability Framework</th><th>Examples of Interview Questions</th></tr>
<tr><td rowspan="2">INTERVENTION: SCIP</td><td rowspan="2">• Components
• Providers
• Delivery platform
• Outcomes</td><td>Structured questions
• What is the process by which you track and report per-operative antibiotics administration (SCIP INF1) and post-operative antibiotics administration (SCIP INF 3)? [Answer choices: A. National reporting; B. Local reporting; C. Infection control; D. Don't know; F. Other]
o Who is involved in this process? (Listen for/probe: infection control/infectious diseases, surgery, nursing, other, don't know)
o To what extent has the process you used for tracking and reporting SCIP INF1 and INF3 evolved over the last few years since SCIP was discontinued? (Listen for/probe: no change, increased/decreased since SCIP discontinuation, other)
• What types of practices has your specialty implemented to help with antibiotics administration (SCIP INF 1 and INF 3) compliance? [Answer choices: A. Protocols and guidelines; B. Checklists; C. Time outs; D. Order sets; E. Integrated into medication reconciliation process; F. NP/PA/resident training; G. Other; H. Not applicable]
o Who is involved in these practices? (Listen for/probe: Infectious diseases/infection control, anesthesia, surgery, pharmacy, combination, other)
o How have those practices evolved, if at all, over the last few years since SCIP was discontinued?
• Who makes decisions about peri-operative antibiotics administration in your facility? What about post-operative antibiotics administration? [Answer choices: A. Attending surgeon; B. Surgical staff; C. Anesthesia; D. Pharmacy; E. Infectious Diseases; F. Other]</td></tr>
<tr><td>Open-ended questions
• What do you think are the best ways to prevent surgical site infections?
o Are you familiar with SCIP Program? If yes:
▪ When SCIP INF metrics were initially implemented, did they change your own antimicrobial practice? If so, how?
▪ Since SCIP was discontinued, how has your practice evolved, if at all?
▪ Have you continued to maintain SCIP INF1 and INF3 equally? Why or why not?
• What do you think has helped to facilitate antibiotics administration compliance (SCIP INF 1 and INF 3) in your specialty? What has hindered antibiotics administration compliance in your specialty at your facility?
• How do you think about the SCIP infection metrics (INF1 and INF 3) differently in terms of antimicrobial administration practice? (Listen for/probe: Benefits? Risk/Harms? Challenges?)</td></tr>
</table>

**PRACTICE SETTING (CONTEXT): Surgical Specialty**	• Resources<br>• Org. culture<br>• Training<br>• Supervision	**Structured questions**<br>• What resources enable you to maintain reporting and tracking processes? What about antimicrobial administration practices? [Answer choices: A. Local surveillance; B. Training/Educational programs; C. Other]<br>o How have these resources helped your specialty with antimicrobial use and compliance?<br>o Are there any resources that you think your specialty needs to help with antimicrobial use and compliance?<br>o What types of resources were provided, if at all, over the last few years since SCIP was discontinued?<br>• Who is the champion of the processes related to guideline concordant antimicrobial use in your specialty? [Answer choices: A. Surgery; B. Anesthesia; C. Infectious Diseases; D. Infection Control; E. Nursing; F. Hospital leadership; G. Other]<br>o How has that evolved over the last few years since SCIP was discontinued?
		**Open-ended questions**<br>• Are there any other factors that impact the ability of your specialty to maintain antibiotics administration compliance? (Listen for/probe: leadership support, facility infrastructure, time, availability of personnel, budget)<br>• How do you think the culture of your specialty at your facility has affected the processes/practices related to antibiotics administration compliance (SCIP INF 1 and INF 3)? (listen for/probe: changes after SCIP was discontinued)
**ECOLOGICAL SYSTEM: VA Facility/VISN**	• Policy<br>• Regulation<br>• Population characteristics	**Structured questions**<br>• What kind of local, VISN, or national performance measures, policies, regulations, or guidelines are being used to track and report antimicrobial use? [Answer choices: A. Maintained previous process; B. Locally developed process; C. Not maintained; D. Other]<br>o Which of these have been most helpful? Least helpful?<br>o How have these policies/regulations/guidelines evolved, if at all, over the last few years since SCIP was discontinued?<br>• To what extent has the discontinuation of SCIP affected your facility’s ability to meet these measures, policies, regulations, or guidelines? [Answer choices: A. A lot; B. Some; C. A little; D. Not at all; E. Other]
		**Open-ended questions**<br>• Has the patient population for your facility had an effect on the antimicrobial use and compliance?<br>o What type of impact?<br>o Were there adaptations that you applied to SCIP because of your facility’s patient population?<br>• Did SCIP change your practice for surgeries that were not included under the umbrella of the program? If so, how?

OMB Number: 4040-0010
Expiration Date: 12/31/2022

RESEARCH & RELATED Senior/Key Person Profile (Expanded)

PROFILE - Project Director/Principal Investigator

Prefix: First Name*: Hillary Middle Name Jane Last Name*: Mull Suffix:

Position/Title*: Investigator
Organization Name*: VA Boston Healthcare System
Department:
Division:
Street1*: 150 South Huntington Ave
Street2:
City*: Boston
County:
State*: MA: Massachusetts
Province:
Country*: USA: UNITED STATES
Zip / Postal Code*: 02130-4817

Phone Number*: 857-364-2766 Fax Number:

E-Mail*: hillary.mull@va.gov

Credential, e.g., agency login: HJMULL@BU.EDU

Project Role*: PD/PI Other Project Role Category:

Degree Type: SCD,MPP Degree Year: 2010,2003

Attach Biographical Sketch*: File Name: VA_Mull_Biosketch.pdf

Attach Current & Pending Support: File Name: VA_Mull_Support.pdf

PROFILE - Senior/Key Person			
Prefix:	First Name*: Westyn	Middle Name	Last Name*: Branch-Elliman	Suffix:

Position/Title*: Infectious diseases consultant & investigator
Organization Name*: Veterans Affairs
Department:
Division:
Street1*: 1400 VFW Parkway
Street2:
City*: West Robury
County:
State*: MA: Massachusetts
Province:
Country*: USA: UNITED STATES
Zip / Postal Code*: 021320000

Phone Number*: 857-203-5116 Fax Number:

E-Mail*: westyn.branch-elliman@va.gov

Credential, e.g., agency login: WBRANCHELLIMAN

Project Role*: PD/PI Other Project Role Category:

Degree Type: MD,MMSC Degree Year: 2006,2012

Attach Biographical Sketch*: File Name: VA_BranchElliman_Biosketch.pdf

Attach Current & Pending Support: File Name: VA_BranchElliman_Support.pdf

PROFILE - Senior/Key Person				
Prefix:	First Name*: Marlena	Middle Name	Last Name*: Shin	Suffix:

Position/Title*: Research Health Scientist
Organization Name*: VA Boston Healthcare Center
Department:
Division:
Street1*: 150 South Huntington Ave (152M)
Street2:
City*: Boston
County:
State*: MA: Massachusetts
Province:
Country*: USA: UNITED STATES
Zip / Postal Code*: 021304817

Phone Number*: 8573642767 Fax Number:

E-Mail*: marlena.shin@va.gov

Credential, e.g., agency login: mhshin

Project Role*: Co-Investigator Other Project Role Category:

Degree Type: JD. MPH Degree Year: 2007, 2008

Attach Biographical Sketch*: File Name: VA_Shin_Biosketch.pdf

Attach Current & Pending Support: File Name: VA_Shin_Support.pdf

PROFILE - Senior/Key Person

Prefix: First Name*: Ryann Middle Name Last Name*: Engle Suffix:

Position/Title*: Research Health Scientist
Organization Name*: VA Boston Healthcare System
Department:
Division:
Street1*: 150 South Huntington Ave
Street2:
City*: Boston
County:
State*: MA: Massachusetts
Province:
Country*: USA: UNITED STATES
Zip / Postal Code*: 02130-4817

Phone Number*: 857-364-2621 Fax Number:

E-Mail*: ryann.engle@va.gov

Credential, e.g., agency login:

Project Role*: Co-Investigator Other Project Role Category:

Degree Type: MPH Degree Year: 2003

Attach Biographical Sketch*: File Name: VA_Engle_Biosketch.pdf

Attach Current & Pending Support: File Name: VA_Engle_Support.pdf

PROFILE - Senior/Key Person

Prefix: First Name*: Anashua Middle Name RANI Last Name*: Elwy Suffix:

Position/Title*: Research Health Scientist
Organization Name*: Edith Nourse Rogers Memorial Veterans Hospital
Department:
Division:
Street1*: Center for Health Quality
Street2: 200 Springs Road (152)
City*: Bedford
County:
State*: MA: Massachusetts
Province:
Country*: USA: UNITED STATES
Zip / Postal Code*: 017300000

Phone Number*: 781-687-2000 x 6707 Fax Number: 781-687-3106

E-Mail*: relwy@bu.edu

Credential, e.g., agency login: ARELWY

Project Role*: Co-Investigator Other Project Role Category:

Degree Type: PHD,MS Degree Year: 2001,1995

Attach Biographical Sketch*: File Name: VA_Elwy_Biosketch.pdf

Attach Current & Pending Support: File Name: VA_Elwy_Support.pdf

PROFILE - Senior/Key Person

Prefix: First Name*: Mary Middle Name Last Name*: Hawn Suffix:

Position/Title*: Staff Surgeon
Organization Name*: Palo Alto VA Health Care System
Department:
Division:
Street1*: 3801 Miranda Drive
Street2:
City*: Palo Alto
County:
State*: CA: California
Province:
Country*: USA: UNITED STATES
Zip / Postal Code*: 94304-1207

Phone Number*: 650-493-5000 Fax Number:

E-Mail*: mhawn@stanford.edu

Credential, e.g., agency login: maryhawn

Project Role*: Co-Investigator Other Project Role Category:

Degree Type: MD,MPH Degree Year: 1987

Attach Biographical Sketch*: File Name: VA_Hawn_Biosketch.pdf

Attach Current & Pending Support: File Name: VA_Hawn_Support.pdf

PROFILE - Senior/Key Person

Prefix: First Name*: Rebecca Middle Name Last Name*: Lamkin Suffix:

Position/Title*: Research Health Scientist
Organization Name*: VA Boston Healthcare System
Department:
Division:
Street1*: 150 South Huntington Ave
Street2:
City*: Boston
County:
State*: MA: Massachusetts
Province:
Country*: USA: UNITED STATES
Zip / Postal Code*: 021304817

Phone Number*: 857-364-6058 Fax Number:

E-Mail*: rebecca.lamkin@va.gov

Credential, e.g., agency login: rlamkin

Project Role*: Other (Specify) Other Project Role Category: Project Manager

Degree Type: MA Degree Year: 1993

Attach Biographical Sketch*: File Name: VA_Lamkin_Biosketch_NotRequired.pdf

Attach Current & Pending Support: File Name: VA_Lamkin_Support_NotRequired.pdf

PROFILE - Senior/Key Person

Prefix: First Name*: Daniel Middle Name Last Name*: Sturgeon Suffix:

Position/Title*: Analyst
Organization Name*: VA Boston Healthcare System
Department:
Division:
Street1*: 150 South Untington Ave
Street2:
City*: Boston
County:
State*: MA: Massachusetts
Province:
Country*: USA: UNITED STATES
Zip / Postal Code*: 02130-4817

Phone Number*: (857) 364-2304 Fax Number:

E-Mail*: daniel.sturgeon@va.gov

Credential, e.g., agency login:

Project Role*: Other (Specify) Other Project Role Category: Analyst

Degree Type: Degree Year:

Attach Biographical Sketch*: File Name: VA_Sturgeon_Biosketch_NotRequired.pdf

Attach Current & Pending Support: File Name: VA_Sturgeon_Support_NotRequired.pdf

PROFILE - Senior/Key Person

Prefix: First Name*: Kathryn Middle Name Last Name*: Colborn Suffix:

Position/Title*: Assistant Professor
Organization Name*: University of Colorado Anschutz Medical Campus
Department:
Division:
Street1*: 13199 E Montview Blvd
Street2: Suite 300, Room 345
City*: Aurora
County:
State*: CO: Colorado
Province:
Country*: USA: UNITED STATES
Zip / Postal Code*: 800450000

Phone Number*: 3039463578 Fax Number:

E-Mail*: kathryn.colborn@ucdenver.edu

Credential, e.g., agency login: k.colborn

Project Role*: Other (Specify) Other Project Role Category: Statistican

Degree Type: PHD,MPH Degree Year: 2013,2005

Attach Biographical Sketch*: File Name: VA_Colborn_Biosketch.pdf

Attach Current & Pending Support: File Name: VA_Colborn_Support.pdf

PROFILE - Senior/Key Person

Prefix:	First Name*: Joseph	Middle Name	Last Name*: Francis	Suffix:

Position/Title*: Chief Improvement and Analytics Officer
Organization Name*: Veterans Health Administration
Department:
Division:
Street1*: 810 Vermont, NW
Street2:
City*: Washington
County:
State*: DC: District of Columbia
Province:
Country*: USA: UNITED STATES
Zip / Postal Code*: 204200000

Phone Number*: (202) 302-3310 Fax Number:

E-Mail*: joseph.francis@va.gov

Credential, e.g., agency login:

Project Role*: Other (Specify) Other Project Role Category: Operational Support

Degree Type: MD Degree Year: 1984

Attach Biographical Sketch*: File Name: VA_Francis_Biosketch_NotRequired.pdf

Attach Current & Pending Support: File Name: VA_Francis_Support_NotRequired.pdf

PROFILE - Senior/Key Person

Prefix:	First Name*: Kelly	Middle Name	Last Name*: Echevarria	Suffix:

Position/Title*: National Clinical Pharmacy Manager, ID Div.
Organization Name*: Department of Veterans Affairs
Department:
Division:
Street1*: 5000 S 5th Ave
Street2:
City*: Hines
County:
State*: IL: Illinois
Province:
Country*: USA: UNITED STATES
Zip / Postal Code*: 601410000

Phone Number*: 708-786-7861 Fax Number:

E-Mail*: Kelly.Echevarria@va.gov

Credential, e.g., agency login:

Project Role*: Other (Specify) Other Project Role Category: Operational Support

Degree Type: PharmD Degree Year: 1997

Attach Biographical Sketch*: File Name: VA_Echevarria_Biosketch_NotRequired.pdf

Attach Current & Pending Support: File Name: VA_Echevarria_Support_NotRequired.pdf

PROFILE - Senior/Key Person

Prefix: First Name*: Hillary Middle Name Last Name*: Babcock Suffix:

Position/Title*: President, SHEA
Organization Name*: Society for Healthcare Epidemiology of America (SHEA)
Department:
Division:
Street1*: 1300 Wilson Boulevard
Street2: Suite 300
City*: Arlington
County:
State*: VA: Virginia
Province:
Country*: USA: UNITED STATES
Zip / Postal Code*: 222090000

Phone Number*: 703-684-1006 Fax Number:

E-Mail*: info@shea-online.org

Credential, e.g., agency login:

Project Role*: Other (Specify) Other Project Role Category: Operational Support

Degree Type: MD, MPH Degree Year: 1994,2006

Attach Biographical Sketch*: File Name: VA_Babcock_Biosketch_NotRequired.pdf

Attach Current & Pending Support: File Name: VA_Babcock_Support_NotRequired.pdf

OMB No. 0925-0001 and 0925-0002 (Rev. 03/2020 Approved Through 02/28/2023)

BIOGRAPHICAL SKETCH

Provide the following information for the Senior/key personnel and other significant contributors.
Follow this format for each person. **DO NOT EXCEED FIVE PAGES.**

NAME: Hillary Jane Mull

eRA COMMONS USER NAME (credential, e.g., agency login): hjmull@bu.edu

POSITION TITLE: Investigator

EDUCATION/TRAINING *(Begin with baccalaureate or other initial professional education, such as nursing, include postdoctoral training and residency training if applicable. Add/delete rows as necessary.)*

INSTITUTION AND LOCATION	DEGREE *(if applicable)*	Completion Date MM/YYYY	FIELD OF STUDY
University of Michigan, Ann Arbor, MI	B.A.	05/2000	Japanese language
University of Michigan, Ann Arbor, MI	M.P.P.	05/2003	Public policy
Boston University, Boston, MA	Ph.D.	09/2011	Health services research

A. Personal Statement

I enthusiastically embrace my role as Multiple Principal Investigator and will dedicate 25% of my allotted time towards completing the scientific aims of this project. I am currently a Research Investigator with the Center for Healthcare Organization and Implementation Research (CHOIR) and a Research Assistant Professor in the Department of Surgery at Boston University School of Medicine. I completed the AMIA 10x10 course in 2014 received a certification in health informatics. In 2011, I completed my PhD in health services research and I am pursuing a research career in surgical quality measurement and patient safety. I was awarded a VISN 1 CDA to examine outpatient surgery in VISN 1 followed by an HSR&D CDA to develop and validate an adverse event surveillance model for outpatient surgery – this is the foundation of the proposed IIR work. In my decade long tenure at the VA, I have developed an expertise in mining VA medical record data, merging VA and CMS records, developing complex statistical models in SAS, Stata and R programming languages, and directing chart review to detect adverse events. I have a long history of collaborating with clinician researchers to design and execute studies that benefit care provided to VA patients. I have completed several research studies directly related to health services research on surgical care and in close collaboration with my Multiple Principal Investigator, Dr. Branch-Elliman, all of which will benefit this IIR:.

1. **Mull, H.J.**, Stolzmann, K., Kalver, E., Schweizer, M.L., Asundi, A., Mehta, P., Stanislawski, M., Branch-Elliman, W. *Novel Methodology to Measure Pre-Procedure Antimicrobial Prophylaxis: Integrating Text Searches with Structured Data from the Veterans Health Administration's Electronic Medical Record*. 2020. BMC Medical Informatics Decision Making, 20(1):15.
2. Linsenmeyer, K., Branch-Elliman, W., Kalver, E., **Mull**, **H.J.** *Surgical Site Infections in Outpatient Surgeries: Less Invasive Procedures Contribute Substantially to the Overall Burden*. 2019. Infection Control & Hospital Epidemiology, 40(10):1191-1193.
3. **Mull, H.J.**, Gellad, Z.F., Gupta, R.T., Valle, J.A., Makarov, D.V., Silverman, T., Branch-Elliman, W. *Factors Associated with Emergency Department Visits and Hospital Admissions After Invasive Outpatient Procedures in the Veterans Health Administration.* 2018. JAMA Surgery, 153(8):774-776.
4. **Mull, H.J.,** Itani, K.M.F., Charns, M.P., Pizer, S.D., Hawn, M.T., Rosen, A.K. *The Nature and Severity of Adverse Events in Select Outpatient Surgeries in the Veterans Health Administration*. 2018. Quality Management in Health Care, 27(3):136-144.

B. Positions and Honors

Positions and Employment

2001-2002 Research Assistant, Michigan Retirement Research Center (MRRC), Ann Arbor, MI
2002 Intern, Health Insurance Association of America (HIAA), Washington, DC
2003-2006 Health Policy Analyst, Altarum Institute, Ann Arbor, MI
2006-2009 Pre-Doctoral Research Fellow, VA Center for Health Quality Outcomes and Economic Research, Bedford, MA
2009-2011 Research Health Scientist, Center for Organization, Leadership, and Management Research (COLMR), VA Boston Healthcare System, Boston, MA
2011- Investigator, CHOIR, VA Boston Healthcare System, Boston, MA
2011- Research Assistant Professor of Surgery, Boston University School of Medicine, Boston, MA

Other Experience and Professional Memberships

2002-2003 Founder and Co-President, Health Policy Students of Ford School of Public Policy
2005- Member, AcademyHealth
2007-2009 President, Boston University AcademyHealth Student Chapter (Vice President in 2007)
2014 Certified in Healthcare Informatics, VA AMIA 10x10
2015 Abstract Reviewer, AcademyHealth Annual Research Meeting (Quality Improvement Theme)
2015 Certificate of Completion, "Maximum Likelihood Estimation" statistics course at the Interuniversity Consortium for Political and Social Research (ICPSR)
2016 Certificate of Completion, "Applied Multilevel Models" statistics course at the ICPSR

C. Contributions to Science

1. Developing electronic trigger algorithms to detect adverse events in outpatient surgery.
In continued work to measure patient safety events in order to improve quality of care, I shifted my focus to address safety in outpatient surgery. My VA HSR&D CDA focused on adverse event surveillance using electronic trigger tools and a complex multilevel logistic regression model to predict surgeries likely to have a 30-day adverse event. I have compared different surgical quality measures in the VA to determine how well they correlate in identifying high and low performing hospitals, and I have looked at the validity of trigger tools in identifying outpatient surgical adverse events missed by VASQIP.

a. **Mull**, **H.J.**, Rosen, A.K., Charns, M.P., O'Brien, W.J., Hawn, M.T., Itani, K.M.F., Pizer, S.D. *Emergency Department Use after Outpatient Surgery Among Dually-Enrolled VA and Medicare Patients?* 2019. Quality Management in Healthcare, 28(4):191-199.

b. **Mull, H.J.**, Rosen, A.K., O'Brien, W.J., McIntosh, N., Hawn, M.T., Itani, K.M.F., Pizer, S.D. *Factors Associated with Hospital Admission after Outpatient Surgery in the Veterans Health Administration.* 2018. Health Services Research, 53(5):3855-3880.

c. Rosen, A.K., **Mull**, **H.J.**, Kaafarani, H., Nebeker, J.R., Shimada, S.L., Helwig, A., Nordberg, B., Long, B., Savitz, L.A., Shanahan, C.W., Itani, K.M.F. *Applying Trigger Tools to Detect Adverse Events Associated with Outpatient Surgery*. 2011. Journal of Patient Safety, 7(1):45-59.

2. Identifying adverse events with manual and electronic "trigger" algorithms.
Patient safety surveillance and the identification of specific safety events remains a challenge in VA healthcare. I have worked with colleagues to test both a manual detection process using the Institute for Healthcare Improvement (IHI)'s Global Trigger Tool (GTT) and electronic triggers based on extract clinical data from the electronic medical record. The first trigger study was funded by the Agency for Healthcare Research and Quality (AHRQ) to develop and test several triggers to flag potential drug and surgical safety events using outpatient electronic medical data. I completed training in healthcare informatics and programmed electronic triggers to detect adverse drug and surgical events. This work involved validating the trigger tools using independent chart review. The second was a pilot test of the IHI GTT in one VA hospital. This work required training staff, collecting chart reviewed data and merging adverse event results with existing VA patient safety datasets.

a. **Mull, H.J.**, Brennan, C., Folkes, T., Hermos, J., Chan, J., Rosen, A.K., Simon, S.R. *Identifying Previously Undetected Harm: Piloting the Institute for Healthcare Improvement's Global Trigger Tool in the Veterans Health Administration.* 2015 Quality Management in Health Care. 24(3):140-6.

b. **Mull, H.J.**, Rosen, A.K., Shimada, S.L., Rivard, P., Nordberg, B., Long, B., Hoffman, J.M., Leecaster, M., Savitz, L.A., Shanahan, C.W., Helwig, A., Nebeker, J.R. *Assessing the Potential Adoption and Usefulness of Concurrent, Action-Oriented, Electronic Adverse Drug Event Triggers Designed for the Outpatient Setting*. 2015 eGEMS (Generating Evidence & Methods to improve patient outcomes), 3 (1).

c. **Mull, H.J.** & Nebeker, J.R. *Informatics Tools for the Development of Action-Oriented Adverse Drug Event Triggers*, 2008. AMIA Annual Symposium Proceedings. Nov 6:505-9.

3. Use administrative data-based tools to identify patient safety problems in inpatient surgery.
Patient safety is of significant concern in the healthcare VA system. My colleagues and I have used administrative data-based tools to identify 30-day postoperative adverse events and measure inpatient surgical safety. We tested and validated the Agency for Healthcare Research and Quality (AHRQ) Patient Safety Indicators with independent nurse chart review and using a chart-reviewed dataset from the VA Surgical Quality Improvement Program (VASQIP). Therefore, I have substantial experience using both VASQIP and PSI surgical safety and quality measures and I am also expert at merging VA administrative data with VASQIP data to cross-compare patient outcomes. This work yielded the following publications:

a. Dasinger, E., Graham, L.A., Wahl, T.S., Richman, J.S., Baker, S.J., Hawn, M.T., Hernandez-Boussard, T., Rosen, A.K., **Mull, H.J.**, Copeland, L.A., Whittle, J.C., Burns, E.A., Morris, M.S. *Preoperative Opioid Use and Postoperative Pain Associated with Surgical Readmissions*. 2019 American Journal of Surgery, e-pub ahead of print.

b. **Mull**, **H.J.**, Chen, Q., Shwartz, M., Itani, K.M.F., Rosen, A.K. *Measuring Surgical Quality: Which Measure Should We Trust?* 2014. JAMA Surgery, 149(11):1210.

c. Chen, Q., Tsai, T.C., **Mull**, **H.J.**, Rosen, A.K., Itani, K.M.F. *Using A Composite Readmission Measure to Assess Surgical Quality in the Veterans Health Administration: How Well Does It Correlate with Established Surgical Measures?* 2014. JAMA Surgery, 149(11):1206.

d. **Mull**, **H.J.**, Borzecki, A.M., Chen, Q., Shin, M.H., Rosen, A.K. *Using the AHRQ PSIs to Detect Post-Discharge Adverse Events in the Veterans Health Administration*. 2013. American Journal of Medical Quality, 29(3):213-9.

4. Measuring medical and surgical hospital quality using 30-day hospital readmissions.
Hospital readmissions emerged as an important marker of the quality of medical care received during a patient's inpatient stay. I contributed to two VA study on hospital readmissions, one examining heart failure, pneumonia and myocardial infarction patients and the other looking at 30-day postoperative readmissions. These medical and surgical studies increased my understanding of administrative data-based research, outcomes measurement, and matching VA datasets to model complex patient outcomes.

a. Graham, L., **Mull**, **H.J.**, Wagner, T.H., Morris, M.S., Rosen, A.K., Richman, J.S., Whittle, J., Burns, E., Copeland, L.A., Itani, K.M.F., Hawn, M.T. *Comparison of a Potential Hospital Quality Metric with Existing Metrics for Surgical Quality-Related Readmissions*. 2019. JAMA Open. 2(4):e191313.

b. Chen, Q., **Mull**, **H.J.**, Rosen, A.K., Borzecki, A.M., Pilver, C., Itani, K.M.F. *Measuring Readmissions after Surgery: Do Different Methods Tell The Same Story?* 2016. American Journal of Surgery. 212(1):24-33.

c. O'Brien, W.J., Chen, Q., **Mull**, **H.J.**, Shwartz, M., Borzecki, A.M., Hanchate, A.D., Rosen, A.K. *What Is the Value of Adding Medicare Data in Estimating VA Hospital Readmission Rates?* 2014. Health Services Research. 50(1):40-57.

d. **Mull**, **H.J.**, Chen, Q., O'Brien, W.J., Shwartz, M., Borzecki, A.M., Hanchate, A.D., Rosen, A.K.. *Comparing 2 Methods of Assessing 30-Day Readmissions: What is the Impact on Hospital Profiling in the Veterans Health Administration?* 2013. Medical Care, 51(7): 589-96.

5. Employing qualitative research methods to understand provider perspectives on patient safety.
Engaging providers is critical to changing quality and safety in healthcare. These efforts can be aided with qualitative interviews and consensus processes. In the AHRQ trigger tool project I conducted a consensus process as part of the development of trigger tools for adverse drug events. I have also interviewed surgeons to better understand factors associated with adverse events in the outpatient setting and hosted

a Delphi consensus process to evaluate how to use administrative and electronic clinical data to identify outpatient surgeries.

a. **Mull, H.J.**, Graham, L.A., Morris, M.S., Rosen, A.K., Richman, J.S., Whittle, J., Burns, E., Wagner, T.H., Copeland, L.A., Wahl, T.S., Jones, C., Hollis, R.H., Itani, K.M.F., Hawn, M.T. *Association of Postoperative Readmissions with Surgical Quality Using a Delphi Consensus Process to Identify Relevant Diagnosis Codes.* 2018. JAMA Surgery, 153(8):728-737.

b. **Mull, H.J.**, Rosen, A.K., Charns, M.P., Itani, K.M.F., Rivard, P.E. *Identifying Risks and Opportunities in Outpatient Surgical Patient Safety: A Qualitative Analysis of Veterans Health Administration Staff Perceptions.* 2017. Journal of Patient Safety, ePub ahead of print.

c. **Mull, H.J.**, Rosen, A.K., Rivard, P.E., Itani, K.M.F. *Defining Outpatient Surgery: Perspectives of Surgical Staff in the Veteran's Health Administration (VA).* 2016. American Surgeon. 82(11): 1142-5.

Complete List of Published Work in MyBibliography:
https://www.ncbi.nlm.nih.gov/myncbi/18wd2o-w7c65w/bibliography/public/

D. Additional Information: Research Support and/or Scholastic Performance

<u>Ongoing Research Support</u>

IIR 18-034 Mull (PI) 04/2020 – 03/2024 Role: Principal Investigator
VA HSR&D
How Can We Make Invasive Non-Surgical Procedures Safer? Using Big Data to Identify Adverse Events and Opportunities to Mitigate Harm
This IIR addresses the VA's urgent need for targeted surveillance tools to detect adverse events and inform quality improvement initiatives in invasive non-surgical interventional cardiology, interventional radiology and gastrointestinal endoscopy procedures. The proposed work will test two hypothesized opportunities to update care guidelines or design quality initiatives to mitigate patient harm using surveillance data: whether the patient received inappropriate antibiotics and whether an anesthesiologist was involved in the procedure.

20-004 Mull (PI) 2/2020 – 7/2020 Role: Principal Investigator
VA CHOIR LIP
Association Between Diabetic Foot Ulcers and Invasive Systemic Infections in the Veterans Health Administration: Can Increased Treatment of Localized Infection Prevent Harm?
Role: Principal Investigator
This CHOIR project explores the clinical pathways associated with diabetic foot ulcer treatment and the relationship between localized infection and downstream hospitalizations and mortality from systemic infection.

SDR 19-327 Garrido (PI) 02/2019 – 09/2023 Role: Co-Investigator
VA HSR&D
Community Care/MISSION Act Virtual Research Network
The purpose of this funding is to establish and supervise a Community Care and MISSION Act Virtual Research Network, providing leadership over the range of project activities associated with the Network and in support of individual projects supported by MISSION Act research funds.

<u>Completed Research Support</u>

PPO 18-121 Bovin (PI) 06/2019 – 05/2020 Role: Co-Investigator
VA HSR&D
From Screening to Treatment: Mapping Access to Care Pathways for Veterans who Screen Positive for PTSD
The purpose of this pilot study of treatment pathways for patients with Post-Traumatic Stress Disorder is to (1) develop a feasible and efficient method for identifying the initial access step for Veterans with a new positive PTSD screen in primary care; and (2) develop a dataset of system-, provider-, and patient-level variables and begin to describe each initial access step identified.

IIR 15-139 Mull (PI) 10/2016 – 12/2019 Role: Principal Investigator

VA HSR&D
Cost-Effectiveness of Dabigatran and Warfarin for Veterans with Afib
This study will infer causal relationships between treatments for atrial fibrillation (AF), outcomes and cost. The retrospective observational study uses instrumental variable and propensity score statistical models of outcomes as a function of being prescribed warfarin compared to a target specific oral anticoagulant. Long-term medication costs and treatment costs for adverse outcomes will also be compared by medication.

I21 Branch-Elliman (PI) 10/2018 – 09/2019 Role: Co-Investigator
VA HSR&D Pilot Award
Validation of a Semi-Automated Infection Surveillance Algorithm for Electrophysiology Procedures
This project focused on improving CIED surveillance in the EP laboratory and provided lessons about the utility of using structured EMR diagnosis and treatment data fields for surveillance beyond inpatient care. Evolution of infection prevention programs to include ambulatory and procedural areas is crucial as healthcare delivery is increasingly provided outside of traditional settings.

CDA 13-270 Mull (PI) 10/2014 – 09/2018 Role: Principal Investigator
VA HSR&D
Development of an Adverse Event Surveillance System for Outpatient Surgery
The CDA goal was to improve quality and safety in outpatient surgery through the development and implementation of an adverse event surveillance system that used trigger tools to screen outpatient surgical data in the VA Corporate Data Warehouse. The funding included training programs in healthcare informatics and advanced statistical methods. Mentors included Drs. Kamal Itani, Mary Hawn, Amy Rosen, Steven Pizer and Marty Charns.

IIR 12-358-3 Hawn (PI) 10/2014 - 03/2018 Role: Co-Investigator
VA HSR&D
Improving Surgical Quality: Risks and Impact of Readmission
Hospital readmissions are associated with increased costs, resource utilization, and poor patient outcomes. To understand determinants of surgical readmission, we performed a retrospective cohort study of patients undergoing inpatient surgery within VA from 2007 to 2012. These data were used to develop a readmission risk prediction tool. We developed a classification system for readmission reasons that can be incorporated into the VA Surgical Quality Improvement Program and examined processes of care in the index admission associated with readmission. We prospectively explored predisposing and enabling factors associated with surgical readmission and convene a Delphi panel to determine predictability of readmission categories. The development of a risk prediction tool allowing prospective identification of high-risk patients for readmission in combination with identification of additional patient, procedure, social, and caregiver factors predisposing patients to readmission will lead to improved quality, safety, and efficiency for VA surgical care.

0152A1 Rosen (PI) 10/2012 – 09/2018 Role: Scientific Coordinator
VA NCPS
Patient Safety Center of Inquiry (PSCI) on Measurement to Advance Patient Safety (MAPS)
The Center's objectives were to advance the field of patient safety measurement, develop a comprehensive assessment of patient safety issues at selected VA facilities, and use this information to improve the quality and safety of health care through targeted quality improvement efforts and translation of findings into clinical practice. The PSCI was centered around three main projects focusing on three domains of patient safety measurement currently used in the VA: 1) data-based surveillance (including administrative and clinical databases); 2) chart review methods; and 3) provider-based incident reporting methods.

V1CDA2013-21 Mull (PI) 3/2014 – 8/2014 Role: Principal Investigator
VISN 1 Career Development Award (CDA)
Characteristics of Outpatient Surgery in VISN 1 Medical Facilities
The overall study goal was to enhance knowledge about outpatient surgery in VA, particularly in VISN 1. Through a comprehensive research and training program with a team of expert mentors, I developed the skills and experience necessary to establish a VA career in healthcare informatics and patient safety, specifically in the development of adverse event detection tools relevant to outpatient care.

OMB No. 0925-0001 and 0925-0002 (Rev. 03/2020 Approved Through 02/28/2023)

BIOGRAPHICAL SKETCH

Provide the following information for the Senior/key personnel and other significant contributors.
Follow this format for each person. **DO NOT EXCEED FIVE PAGES.**

NAME: Westyn Branch-Elliman, MD, MMSc

eRA COMMONS USER NAME (credential, e.g., agency login): WBRANCHELLIMAN

POSITION TITLE: Assistant Professor of Medicine, Harvard Medical School

EDUCATION/TRAINING *(Begin with baccalaureate or other initial professional education, such as nursing, include postdoctoral training and residency training if applicable. Add/delete rows as necessary.)*

INSTITUTION AND LOCATION	DEGREE *(if applicable)*	Completion Date MM/YYYY	FIELD OF STUDY
University of California, Berkeley. Berkeley, CA	B.A.	05/2002	Molecular & Cell Biology
Northwestern University Feinberg School of Medicine. Chicago, IL	M.D.	05/2006	Medicine
Harvard Medical School. Boston, MA	M.M.Sc.	05/2012	Clinical Research
Mount Sinai School of Medicine. New York, NY	Internship & Residency	06/2009	Internal Medicine
Beth Israel Deaconess Medical Center. Boston, MA	Fellowship	11/2013	Infectious Diseases & Infection Control
NIH Training in the Dissemination and Implementation of Research in Health (TIDIRH)	Certificate	1/2020	Implementation Science

A. Personal Statement

Dr. Branch-Elliman is an accomplished early-stage investigator who has made highly impactful contributions to infection prevention, antimicrobial stewardship, and epidemiology. She is an Assistant Professor of Medicine at Harvard Medical School, an investigator at the VA Boston Center for Healthcare Organization and Implementation Research (CHOIR) and a staff infectious diseases physician at the West Roxbury VA Medical Center and Beth Israel Deaconess Medical Center (BIDMC). Formerly, she was the Medical Director of Infection Prevention at the Eastern Colorado VA HCS and an Assistant Professor at the University of Colorado. Dr. Branch-Elliman has an outstanding track record of peer-reviewed publications, including multiple studies co-authored with the study co-PI, Dr. Hillary Mull. In 2015, she received the Infectious Diseases Society of America Investigator Award for delineating the comparative effectiveness of different antimicrobial surgical prophylaxis regimens. Dr. Branch-Elliman is the recipient of an NHLBI K12 award, VA I21 pilot funding which directly resulted in the research in this IIR, and formerly an American Heart Association Precision Medicine Grant. Dr. Branch-Elliman also successfully competed for NIH funding through Harvard Catalyst/Clinical and Translational Sciences Institute and received VA T21 Research Funds through the Geriatric Research and Education Center.

Dr. Branch-Elliman's research background makes her ideally suited to be the Multiple PI of the proposed VA HSR&D IIR. The goal of this project is to use advanced data mining strategies to evaluate the sustainability and diffusion of antimicrobial use best practices since the discontinuation of the Surgical Care Improvement Project (SCIP). The proposed research builds upon Dr. Branch-Elliman's expertise in peri-procedural antimicrobial stewardship, including measurement of adverse events associated with guideline-discordant practices, and upon her VA HSR&D Pilot grant. Dr. Branch-Elliman's prior research includes high-impact work establishing a clear link between each additional day of guideline-discordant antimicrobial use and post-operative harm (Branch-Elliman et al, *JAMA Surgery*). Another investigation published in *PLoS Medicine* established a limited role for combination prophylaxis for reducing surgical site infections and used the same data sources as in these

proposed investigations, enhancing project feasibility. In collaboration with Multiple PI Dr. Hillary Mull, Dr. Branch-Elliman also demonstrated that facility and procedure factors play a large role in driving post-operative antimicrobial use. As part of her participation in the NHLBI K12 program and the NIH Training Institute for Dissemination and Implementation Research in Health (TIDIRH), she gained training and hands on experience with implementation science methodology and will apply this knowledge in the proposed research.

Publications Most Relevant to Proposed Project:

1. **Branch-Elliman W**, Obrien W, Strymish J, Itani KM, Wyatt C, and Gupta K. Association of Duration and Type of Surgical Prophylaxis with Antimicrobial-Associated Adverse Events. *JAMA Surgery*. Published online April 29,2019.
2. Linsenmeyer, K., **Branch-Elliman, W**., Kalver, E., Mull, H.J**.** *Surgical Site Infections in Outpatient Surgeries: Less Invasive Procedures Contribute Substantially to the Overall Burden*. 2019. Infection Control & Hospital Epidemiology, 40(10):1191-1193.
3. **Branch-Elliman W**, Ripollone JE, O'Brien WJ, Itani KMF, Schweizer ML, Perencevich E, Strymish J, and Gupta K. Risk of surgical site infection, acute kidney injury, and *Clostridium difficle* infection following antibiotic prophylaxis with vancomycin plus a beta-lactam versus either drug alone: A national propensity-score adjusted retrospective cohort study. *Plos Med*. 2017 Jul 10; 14(7):e1002340.
4. **Branch-Elliman W**, Pizer S., Dasinger E, Gold HS, Abdulkerim H, Rosen AK, Charns M, Hawn, M, Itani KM, Mull, HJ. Facility Type and Surgical Specialty Are Associated with Suboptimal Surgical Antimicrobial Prophylaxis Practice Patterns: A Multi-Center, Retrospective Cohort Study. *Antimicrob Resist Infect Control*. 2019. Mar 6; 8-49.

B. Positions and Honors

Honors, Awards and Scholarships:

1999	Mark of Excellence for Best General News Reporting	Society for Professional Journalists
2010-12	Harvard Scholars in Clinical Sciences Program	Harvard Catalyst
2011	SHEA Travel Award	Society for Healthcare Epidemiology in America (SHEA)
2012	Finalist, Rice Business Plan Competition	Rice University Alliance for Technology and Entrepreneurship
2012	First Place, Residents and Fellows Research Competition	Beth Israel Deaconess Medical Center
2013	Jonathan Freeman Scholarship	SHEA
2013	ID Week Travel Award	Infectious Diseases Society of America
2015	IDSA Investigator Award	Infectious Diseases Society of America
2017	Top Reviewer	*Infection Control and Hospital Epidemiology*
2019	Top Poster	SHEA

Positions and Employment:

2001	Research Assistant to CDC Officer Michael Phillips, MD	New York City Department of Health
2005-6	Research Assistant to Steven Flamm, MD	Northwestern Memorial Hospital
2006-9	Intern and Resident (Internal Medicine)	Mount Sinai School of Medicine
2009-13	Fellow (Infectious Diseases and Infection Control)	Beth Israel Deaconess Medical Center
2014-16	Assistant Professor of Medicine	Univ. of Colorado School of Medicine
2016-17	Instructor in Medicine	Harvard Medical School
2017-	Assistant Professor of Medicine	Harvard Medical School

Journal Reviewer:

2014-	Infection Control/Hospital Epidemiology	Ad-Hoc Reviewer
2014-	Clinical Infectious Diseases	Ad-Hoc Reviewer
2014-	British Medical Journal	Ad-Hoc Reviewer
2016-	American Journal of Infection Control	Ad-Hoc Reviewer
2016-	World Journal of Gastroenterology	Ad-Hoc Reviewer

Licensure and Certifications:

2007	Diplomat	National Board of Medical Examiners
2009	Diplomat, Internal Medicine	American Board of Internal Medicine
2010-	Massachusetts Medical License	Commonwealth of Massachusetts
2011	Diplomat, Infectious Diseases	American Board of Internal Medicine

Clinical Appointments:

2011-12	Attending, Urgent Care	Harvard Vanguard Medical Associates
2012-13	Attending, Infectious Diseases	Harvard Vanguard Medical Associates
2014-6	Attending, Infectious Diseases	Eastern Colorado VA HCS
2014-15	Associate Medical Director of Infection Prevention	Eastern Colorado VA HCS
2015-6	Medical Director of Infection Prevention	Eastern Colorado VA HCS
2016-	Staff Infectious Diseases Physician	VA Boston HCS
2016-	Infectious Diseases Consultant	Beth Israel Deaconess Medical Center Boston, MA

C. Contributions to Science

The central aim of Dr. Branch-Elliman's research is to identify and implement strategies to prevent procedure-related adverse events and to improve the delivery of infection prevention to optimize patient outcomes in a clinically and cost-effective manner. A major focus is on identifying adverse events that result from actions providers apply with the intention of reducing adverse events, and on measuring how these harms are weighed against potential benefits. Improving the efficiency and reliability of infection detection and reporting, particularly in outpatient and procedural settings of care with limited infection prevention resources, is another major research effort. One of the most important insights arising from Dr. Branch-Elliman's work is the observation that even beneficial infection prevention strategies may negatively impact clinical outcomes if these programs divert resources away from other essential clinical care tasks. A second important contribution is that evidence-based infection prevention programs, which can be highly effective for improving healthcare while containing medical costs, are rarely applied. Her research is enhanced by her hands-on experience with outbreak investigation at the New York City Department of Health and Mental Hygiene and through prior positions as Associate Medical Director of Infection Prevention and Control and then Medical Director of Infection Prevention and Control at the Eastern Colorado VA.

Key experimental findings are highlighted below.

Infection Prevention and Resource Utilization:
Dr. Branch-Elliman developed a comparative effectiveness model to determine the optimal set of infection prevention strategies to reduce ventilator-associated pneumonia in a clinically and cost-effective manner. The evaluation revealed that all preferred infection prevention strategies include the use of strategies that are not commonly implemented in clinical practice (probiotics and endotracheal tubes). Furthermore, Dr. Branch-Elliman found that strategies commonly used (such as the Institute for Healthcare Improvement Ventilator Bundle and oral care) require substantial nursing resources, and that the implementation of some strategies may worsen care, by preventing critical care nurses from completing other essential clinical care tasks, such as timely administration of medications.

1. **Branch-Elliman W,** Wright SB, Howell MD. Determining the Ideal Strategy for Ventilator-Associated Pneumonia Prevention: Cost-Benefit Analysis. *Am J Respiratory and Critical Care Medicine*. July 2015.
2. **Branch-Elliman W**, Wright SB, Gillis J, and Howell MD. Estimated Nursing Workload for the Implementation of Ventilator Bundles. *BMJ Quality and Safety*. February 2013; 22 (4): 357-67.

Comparative Effectiveness of Surgical Prophylaxis Regimens and Risks and Benefits of Different Strategies for Preventing Surgical Site infections:
Dr. Branch-Elliman's research established a clear link between every additional day of antimicrobial exposure and increasing rates of adverse events. Additional high-impact work compared the effectiveness of single versus double agent antimicrobial prophylaxis and found that combination regimens led to decreases in surgical site infections after cardiac surgeries but not other types of procedures. Additional research demonstrates limited

dissemination of evidence-based guidelines, particularly to outpatient and procedural areas and a limited role for screening and treatment of urinary tract infections in the peri-operative period.

1. **Branch-Elliman W**, O'Brien W, Strymish J, Itani KM, Wyatt C, and Gupta K. Association of Duration and Type of Surgical Prophylaxis with Antimicrobial-Associated Adverse Events. *JAMA Surgery*. 2019; April 24. Epub ahead of print.
2. **Branch-Elliman W**, Ripollone JE, O'Brien WJ, Itani KMF, Schweizer ML, Perencevich E, Strymish J, and Gupta K. Risk of Surgical Site Infection, acute kidney injury, and Clostridium difficile infection following antibiotic prophylaxis with vancomycin plus a beta-lactam versus either drug alone: A national propensity-score adjusted retrospective cohort study. PLoS Med. 2017. July 10; 14(7): e1002340.

Infection Prevention and Antimicrobial Stewardship in the Cardiac Electrophysiology Laboratory:
Dr. Branch-Elliman demonstrated that infection prevention strategies—including simple interventions that can be completed at the point of care, such as procedural delay if the patient has an active infection—are rarely implemented in clinical practice. Furthermore, practices that are known to increase adverse events but that do not reduce infection are commonly applied.

1. Mehrotra P, Gupta K, Strymish J, Kramer DB, Lambert-Kerzner A, Ho PM, and **Branch-Elliman W.** Implementation of Infection Prevention and Antimicrobial Stewardship in Cardiac Electrophysiology Laboratories: Results from the Society for Healthcare Epidemiology in America (SHEA) Research Network. *Infection Control & Hospital Epidemiology*. January 2017; 20: 1-2. [Epub ahead of print].
2. **Branch-Elliman W**, Stanislawski M, Strymish J, Baron A, Gupta KG, Varosy PD, Gold HS, Ho PM. Cardiac Electrophysiology Laboratories: A Potential Target for Antimicrobial Stewardship and Quality Improvement? *Infection Control & Hospital Epidemiology*. September 2016; 37(9): 1005-11.
3. Asundi A, Stanislawski M, Mehta P, Baron AE, Gold H, Mull H, Ho PM, Gupta K, and **Branch-Elliman W**. Prolonged antimicrobial prophylaxis following cardiac device procedures increases preventable harm: Insights form the VA CART program. *Infection Control & Hospital Epidemiology*. September 2018; 39(8):947-954.
4. Asundi A, Stanislawski M, Mehta P, Baron AE, Mull H, Ho PM, Zimetbaum P, Gupta K, and **Branch-Elliman W**. Real-World Effectiveness of Infection Prevention Interventions for Reducing Procedure-Related Cardiac Device Infections: Insights from the VA CART program. *Infection Control & Hospital Epidemiology*. 2019. *In press*.

Electronic Detection Systems for Identifying Healthcare-Associated Infections:
In order to improve inpatient infection prevention practice, Dr. Branch-Elliman developed novel electronic detection algorithms for identifying clinical MRSA infections, surgical site infections, and catheter-associated urinary tract infections. The algorithm for surgical site infection has been operationalized for day-to-day surveillance activities within the VA healthcare system. Dr. Branch-Elliman also evaluated the utility of using a clinical history of MRSA infection for predicting pre-operative MRSA colonization status in a cohort of patients at the Boston VA Medical Center, and found that additional screening is necessary to optimize peri-operative care and reduce surgical site infections.

1. **Branch-Elliman W**, Strymish J, Gupta K. Development and Validation of a Simple and Easy-to-Employ Algorithm for Identifying Clinical Methicillin-Resistant *Staphylococcus aureus* Infection. *Infection Control Hospital Epidemiology*. June 2014; 35(6):692-8.
2. **Branch-Elliman W**, Strymish J, Itani KM, Gupta K. Using Clinical Variables to Guide Surgical Site Infection Detection: A Novel Surveillance Strategy. *Am J of Infection Control*. Dec 2014; 42(12):1291-5.
3. **Branch-Elliman W**, Strymish J, Kudesia V, Rosen AK, Gupta K. Natural Language Processing for Real-Time Catheter-Associated Urinary Tract Infection Surveillance: Results of a Pilot Implementation Trial. *Infection Control & Hospital Epidemiology*. September 2015; 36(9):1004-10.
4. Asundi A, Stanislawski M, Mehta P, Mull H, Schweizer M, Ho PM, Gupta K, and **Branch-Elliman W.** Development of an Electronic Flagging Tool for Identifying Cardiac Device Infections: Insights from the VA CART Program. Open Forum Infectious Diseases. November 2018; 5(S1): S15.

Complete List of Published Work in MyBibliography:
http://www.ncbi.nlm.nih.gov/sites/myncbi/1pMeiNIvzLrko/bibliograpahy/40368776/public/?sort=date&direction=ascending

D. Additional Information: Research Support and/or Scholastic Performance

Ongoing Research Support

1K12HL138049-01 Branch-Elliman (PI) 2018-20 Role: PI
NIH NHLBI
Implementation of an Infection Prevention Program for Reducing Cardiac Device Infections Following Invasive Electrophysiology Procedures
The goals of this implementation science training project are to collect formative evaluations for implementation of a comprehensive prevention program in the electrophysiology laboratory and to design an implementation trial.

IIR 18-034 Mull (PI) 2020-24 Role: Co-Investigator
VA HSR&D
How Can We Make Invasive Non-Surgical Procedures Safer? Using Big Data to Identify Adverse Events and Opportunities to Mitigate Harm
This IIR addresses the VA's urgent need for targeted surveillance tools to detect adverse events and inform quality improvement initiatives in invasive non-surgical specialties.

Completed Support

T21 G541-3 Jump (PI) 2014 Role: Site PI
VA
A Multi-Site Education Intervention to Improve the Treatment of Infections Among Older Veterans
The goal of the project was to evaluate how an educational outreach program could improve antimicrobial prescribing practices throughout the VA healthcare system.

Operations Project Branch-Elliman (PI) 2014-17 Role: PI
Denver VA Operations Support
Operationalizing an Automated Surveillance Algorithm for Surgical Site Infection Detection
The study characterized the utility of the electronic detection system and measured implementation barriers.

CDA Branch-Elliman (PI) 2016-18 Role: PI
VISN 1 Career Development Award.
Improving Veterans' Care by Expanding Infection Prevention to Outpatients
The career development award provides salary support to the PI to measure the impact of infection prevention interventions in the cardiac electrophysiology laboratory and to identify potential areas for improving patient safety. The award provides salary support for the PI but no additional research funds.

17IG33630052 Branch-Elliman (PI) 2017-18 Role: PI
American Heart Association Institute for Precision Cardiovascular Medicine Award
Advanced Medical Informatics to Improve the Care of Arrhythmia Patients by Reducing Procedure-Related Cardiac Device Infections
The goal of this project is to develop a database of patients undergoing cardiac device procedures in the electrophysiology laboratory and to 1) to identify modifiable risk factors associated with the development of cardiac device infections and 2) to measure the impact of infection prevention interventions on cardiac device infection outcomes.

I21 HX002302-01A1 Branch-Elliman (PI) 2018-19 Role: PI
VA HSR&D
Validation of a Semi-Automated Infection Surveillance Algorithm for Electrophysiology Procedures
This project aims to refine and validate an electronic surveillance tool for measuring cardiac device infections and to identity novel methods for automating infection measurement in outpatient and procedural areas.

OMB No. 0925-0001 and 0925-0002 (Rev. 03/2020 Approved Through 02/28/2023)

BIOGRAPHICAL SKETCH

Provide the following information for the Senior/key personnel and other significant contributors.
Follow this format for each person. **DO NOT EXCEED FIVE PAGES.**

NAME: Shin, Marlena H.

eRA COMMONS USER NAME (credential, e.g., agency login): mhshin

POSITION TITLE: Research Health Scientist/Investigator

EDUCATION/TRAINING *(Begin with baccalaureate or other initial professional education, such as nursing, include postdoctoral training and residency training if applicable. Add/delete rows as necessary.)*

INSTITUTION AND LOCATION	DEGREE *(if applicable)*	Completion Date MM/YYYY	FIELD OF STUDY
Tufts University	BA	05/2000	International Relations; English
Northeastern University School of Law	JD	05/2007	Health Law; Labor Law
Tufts University School of Medicine	MPH	02/2008	Public Health

A. Personal Statement

I enthusiastically agree with my role as co-investigator on Drs. Mull and Branch-Elliman's VA HSR&D proposal "Assessing the Sustainability of Compliance with Surgical Site infection Prophylaxis After Discontinuation of Mandatory Active Reporting." I will dedicate 20% of my allotted time towards completing the project study aims. As an investigator at the Center for Healthcare Organization and Implementation Research (CHOIR), VA Boston Healthcare System, I devote 100% of my time to research. With 10 years of experience as a VA health services researcher, I have expertise in qualitative methods, implementation science, and organizational science. I have extensive experience in conducting qualitative data collection and analyses and working with various stakeholder groups to implement programs. I was previously the PI on a VA QUERI-funded RRP (How to Interpret and Use Patient Safety Indicator Reports) that developed, implemented, and evaluated an educational program about the AHRQ Patient Safety Indicators. I also have experience serving as the qualitative lead or co-lead on several VA HSR&D and partner-sponsored projects, such as: PSCI: Measurement to Advance Patient Safety (XVA 68-023), Leveraging Front-line Expertise in Quality and Safety (SDP 12-251), Military Sexual Trauma Screening: Examining Patient Satisfaction and Preferences (PPO 14-113-2), and Validating the PSIs in the VA (SDR 07-002). My experience will be very applicable to the proposed study.

- **Shin MH**, Rivard PE, Shwartz M, Borzecki A, Yaksic E, Stolzmann K, Zubkoff L, Rosen AK. Tailoring an educational program on the AHRQ Patient Safety Indicators to meet stakeholder needs: lessons learned in the VA. BMC Health Serv Res. 2018 Feb 14;18(1):114.
- Chen Q, **Shin MH**, Chan JA, Sullivan JL, Borzecki AM, Shwartz M, Rivard P, Hatoun J, Rosen AK. Partnering with VA Stakeholders to Develop a Comprehensive Patient Safety Data Display: Lessons Learned from the Field. Am J Med Qual. 2016; 31(2):178-86.
- **Shin MH**, Sullivan JL, Rosen AK, Solomon JL, Dunn EJ, Shimada SL, Hayes J, Rivard PE. Examining the Validity of AHRQ's Patient Safety Indicators (PSIs): Is Variation in PSI Composite Score Related to Hospital Organizational Factors? Med Care Res Rev. 2014; 71(6):599-618.

B. Positions and Honors

2009-Present Research Health Scientist, Center for Healthcare Organization and Implementation Research (CHOIR) ((formerly Center for Organization Leadership and Management Research), VA Boston Healthcare System, Boston, MA

2009 Research Health Scientist, Center for Health Quality, Outcomes, and Economic Research,

Edith Nourse Rogers Memorial Veterans Hospital, Bedford
2008-2009 Research Coordinator, Boston University, School of Public Health, Boston, MA
2007-2008 Intern, Newton Department of Health and Human Services, Newton, MA
2006-2007 Intern, Massachusetts Department of Public Health, Boston, MA
2005 Intern, U.S. Department of Labor, Office of the Administrative Law Judges, The Honorable Daniel F. Sutton, Boston, MA
2005 Intern, Massachusetts Attorney General's Office, Fair Labor and Business Practices Division, Boston, MA
2004 Intern, U.S. District Court, District of Massachusetts, The Honorable Robert B. Collings, Boston, MA
2000-2003 Legal Assistant, Mintz, Levin, Cohn, Ferris, Glovsky, and Popeo, P.C., Boston, MA

C. Contributions to Science

Much of my research has focused on patient safety and quality measurement. In particular, I have developed an expertise on the Agency for Healthcare Research and Quality (AHRQ) Patient Safety Indicators (PSIs). I have worked on several projects to evaluate the PSIs and how to use the PSIs. In the project, Validating the PSIs in the VA, we used a multi-faceted approach to examine the PSIs. Numerous publications developed from this project; our results being comparable to those of an AHRQ-funded validation study.

- Rosen AK, Itani K, Cevasco M, Kaafarani H, Hanchate A, **Shin MH**, Shwartz M, Loveland S, Chen Qi, Borzecki A. Validating the Patient Safety Indicators (PSIs) in the Veterans Health Administration: Do They Accurately Identify True Safety Events? Med Care. 2012; 50(1):74-85.
- Rosen AK, Loveland S, **Shin MH**, Shwartz M, Hanchate A, Chen Qi, Kaafarani H, Borzecki A. Examining the impact of the AHRQ Patient Safety Indicators (PSIs) in the Veterans Health Administration: The case of readmissions. Med Care. 2013; 51(1):37-44.
- Rosen AK, Chen Qi, Borzecki AM, **Shin MH**, Itani K, Shwartz M. Using Estimated True Safety Event Rates vs. Flagged Safety Event Rates: Does it Change Hospital Profiling and Payment? Health Serv Res. 2014; 49(5):1426-45.

For the same project, we used qualitative methods as another approach to validating the PSIs in the VA. My expertise in qualitative methods further solidified when I co-led the qualitative work in this study. We conducted site visits to three VA hospitals with high and three VA hospitals with low PSI composite scores and interviewed a cross-section of hospital staff to collect data on 13 safety-related domains. Since this study, I have either served as the qualitative lead or team member on several studies that were funded by VA HSR&D, QUERI, Office of Geriatrics and Extended Care, Office of Rural Health, National Center for Patient Safety (NCPS); in these studies, I have gained a tremendous amount of experience in interviewing VA providers and Veterans as well as analyzing qualitative data through these studies.

- **Shin MH**, Sullivan JL, Rosen AK, Solomon JL, Dunn EJ, Shimada SL, Hayes J, Rivard PE. Examining the Validity of AHRQ's Patient Safety Indicators (PSIs): Is Variation in PSI Composite Score Related to Hospital Organizational Factors? Med Care Res Rev. 2014; 71(6):599-618.
- Street AE, **Shin MH**, Marchany KE, McCaughey VK, Bell ME, Hamilton AB. Veterans' perspectives on military sexual trauma-related communication with VHA providers. Psychol Serv. 2019 Oct 17.

I also have extensive experience in program implementation as well as stakeholder education. In Validating the PSIs in the VA, we co-developed and implemented a Virtual Breakthrough Series with the VA National Center for Patient Safety to educate VA stakeholders on one of the PSIs, Postoperative Respiratory Failure. Since then, I have worked on several projects to further build my expertise in implementing and educating VA stakeholders on patient safety and quality measurement. For example, through a VA NCPS Patient Safety Center of Inquiry project, we developed a Guiding Patient Safety tool to provide a more integrated picture of patient safety. We partnered with multiple VA stakeholders and engaged frontline users at 2 hospitals. I was also the PI of a study which developed through stakeholder feedback, implemented, and evaluated a program about the PSIs.

- Zubkoff L, Neily J, Mills P, Borzecki AM, **Shin M**, Lynn MM, Gunnar W, Rosen AK. Using a Virtual Breakthrough Series Collaborative to Reduce Postoperative Respiratory Failure in 16 Veterans Health Administration (VHA) Hospitals. Jt Comm J Qual Patient Saf. 2014; 40(1):11-20.
- Chen Q, **Shin MH**, Chan JA, Sullivan JL, Borzecki AM, Shwartz M, Rivard P, Hatoun J, Rosen AK. Partnering with VA Stakeholders to Develop a Comprehensive Patient Safety Data Display: Lessons Learned from the Field. Am J Med Qual. 2016; 31(2):178-86.
- **Shin MH**, Rivard PE, Shwartz M, Borzecki A, Yaksic E, Stolzmann K, Zubkoff L, Rosen AK. Tailoring an educational program on the AHRQ Patient Safety Indicators to meet stakeholder needs: lessons learned in the VA. BMC Health Serv Res. 2018 Feb 14;18(1):114.

I have developed an expertise in organizational research through several projects that have examined how organizational contextual factors influence process, outcomes, and program implementation for both staff and patients. I also have contributed my expertise in organizational research to various areas of research, such as patient safety and quality, care coordination and transitions of care, and Long-Term Services and Supports.

- Sullivan JL, **Shin MH**, Engle RL, Yaksic E, VanDeusen Lukas C, Paasche-Orlow MK, Starr LM, Restuccia JD, Holmes SK, Rosen AK. Evaluating the Implementation of Project Re-Engineered Discharge (RED) in Five Veterans Health Administration (VHA) Hospitals. Jt Comm J Qual Patient Saf. 2018 Nov;44(11):663-673.
- Sullivan JL, Rivard PE, **Shin MH**, Rosen AK. Applying the High Reliability Health Care Maturity Model to Assess Hospital Performance: A VA Case Study. Jt Comm J Qual Patient Saf. 2016 Sep;42(9):389-411.
- Sullivan JL, Adjognon OL, Engle RL, **Shin MH**, Afable MK, Rudin W, White B, Shay K, Lukas CV. Identifying and overcoming implementation challenges: Experience of 59 non-institutional long-term services and sports pilot programs in the Veterans Health Administration. Health Care Manage Rev. 2018 Jul/Sept; 43(3): 193-205.
- George J, Parker VA, Sullivan JL, Greenan MA, Chan J, **Shin MH**, Chen Q, Shwartz M, Rosen AK. How hospitals select their patient safety priorities: An exploratory study of four Veterans Health Administration hospitals. Health Care Manage Rev. 2019 Oct 25.

Complete List of Published Work in MyBibliography:
https://www.ncbi.nlm.nih.gov/sites/myncbi/10uKXnkVSp0Qx/bibliography/47974367/public/?sort=date&direction=descending

D. Additional Information: Research Support and/or Scholastic Performance

Ongoing Research Support
CIN-14-240 VA HSR&D 05/2019-9/2020
HRO Evaluation (Damschroder)
Our specific aims are to: 1) identify and explore the use of metrics used to monitor progress and impacts of HRO and identify measurement gap; 2) use rapid qualitative analytic approaches to develop insights about strategies and their effects related to HRO Rollout; and 3) set the stage for a randomized program evaluation project.
Role: Co-Investigator

SDR 17-306 (Taylor/ Zeliadt co-PI) VA HSR&D Merit Award 04/2018-3/2024
APPROACH: Assessing Pain, Patient Reported Outcomes and Complementary Health (A National VA Demonstration Project)
A two-year planning grant and four-year pragmatic trial comparing 3 arms of CIH services with 18,000 patients to examine these non-pharmacological approaches to pain management.
Role: Co-Investigator

PEC 18-204 (Rosen PI) VA HSR&D QUERI 11/2018-4/2021
Evaluating the Implementation of Patient Safety Practices to Ensure Timely, High-Quality Community Care for Veterans
The expansion of Community Care (CC), and the increase in the numbers of Veterans using CC, make it critical for VHA to balance the need to improve access to care, and at the same time, ensure that the services

that VHA purchases in the community are of high quality. As there is little known about the quality and safety of care that Veterans receive in the community, this start-up project will begin to close these knowledge gaps.
Role: Co-Investigator

Completed Research Support

VA Office of Rural Health (Miller/Kim co-PI) VA Operational 10/2018 – 9/2019
Veterans' Experiences and Perspectives on Care Coordination between VA and Community Clinics
The major goal of this project is to develop a better understanding of Veterans' experiences of care coordination between VA clinics and Federally Qualified Health Centers (FQHCs) that provide medical and mental health care in rural areas. To develop this understanding, we are conducting and analyzing semi-structured qualitative interviews with Veterans.
Role: Co-Investigator

I21 Branch-Elliman (PI) VA HSR&D Pilot Award 10/2018 – 9/2019
Validation of a Semi-Automated Infection Surveillance Algorithm for Electrophysiology Procedures
This project focuses on improving CIED surveillance in the EP laboratory and provides lessons about the utility of using structured EMR diagnosis and treatment data fields for surveillance beyond inpatient care. Evolution of infection prevention programs to include ambulatory and procedural areas is crucial as healthcare delivery is increasingly provided outside of traditional settings.
Role: Co-Investigator

XVA 68-023 (Rosen) VA NCPS 10/2012–9/2018
Patient Safety Center of Inquiry (PSCI) on Measurement to Advance Patient Safety (MAPS)
MAPS' objectives were to advance the field of patient safety measurement, develop a comprehensive assessment of patient safety issues at selected VA facilities, and use this information to improve the quality and safety of health care through targeted quality improvement and translation of findings into clinical practice.
Role: Co-Investigator

PCC 2016-121158 (Taylor/Elwy) VA HSR&D QUERI 10/2016-9/2018
Complementary and Integrative Health Evaluation Center (CIHEC)
Specific aims: 1) improve our understanding of the details of the provision of CIH across all VA healthcare facilities; 2) advance our understanding of Veterans' preference for and use of CIH; 3) develop procedures for collecting CIH-related patient reported outcomes in clinical care settings; 4) examine the effectiveness of CIH on Veterans' health; and 5) enrich implementation science by comparing facilitation strategies across care settings—and informing QUERI collaborative efforts to refine facilitation approaches.
Role: Co-Investigator

VXA-11-50 (Simons) VISN 2 Suicide Prevention COE/OMHSP 1/2018-9/2018
Examining Veterans Mental and Behavioral Care during care transitions from VA Community Living Centers
The goal of this study is to identify mental health needs of Veterans transitioning from 4 VA CLCs to the community. In particular, we will utilize chart review, a battery of quantitative measures, and qualitative interviews to understand mental health need before and after discharge.
Role: Co-Investigator

PEI 15-468 (Sullivan) VA HSR&D QUERI 1/2016-6/2018
Advancing Healthcare Innovation: A Partnered-Evaluation of Geriatric Patient-Aligned Care Team (GeriPACT) Implementation
Specific aims were to: 1) assess GeriPACT implementation adherence and service outcomes (robust team-based care dimensions); 2) examine the relationship of implementation adherence, service outcomes (robust team-based care dimensions), and patient utilization and GeriPACT costs; 3) compare utilization and cost outcomes for similar GeriPACT and PACT patients over time; and 4) identify important organizational contextual factors associated with GeriPACT performance by conducting an in-depth evaluation at eight VAMCs with high GeriPACT adherence, but vary on other service outcomes related to GeriPACT performance.
Role: Co-Investigator

PEC 15-238 (Charns) VA HSR&D QUERI 5/2015-9/2017
Lean Enterprise Transformation Evaluation Center
The Veterans Health Administration implemented a national program of Lean Enterprise Transformation (LET) to embed Lean principles, strategies, practices and behaviors in medical centers to improve healthcare quality and efficiency. The project evaluated LET implementation in the initial pilot sites and worked closely with its VERC partners and sites to determine strategies for measurement and to put findings into practice.
Role: Co-Investigator

SDP 12-251 (Shwartz) VA HSR&D QUERI 5/2014-9/2017
Leveraging Frontline Expertise in Quality and Safety (LFLE)
The primary aim of the study is to conduct a LFLE intervention and assess implementation processes and factors associated with success, with the secondary aim of assessing LFLE effectiveness in VA.
Role: Co-Investigator

PPO 14-113-2 (Street) VA HSR&D 6/2015-5/2016
Military Sexual Trauma Screening: Examining Patient Satisfaction and Preferences
The goal of this project is to conduct the first evaluation of VHA's Military Sexual Trauma (MST) Screening Program from a perspective that incorporates veterans' preferences.
Role: Co-Investigator

RRP 11-387 (Sullivan) VA HSR&D QUERI 3/2013–6/2014
Evaluating Implementation of Project Re-Engineered Discharge (RED) in VA
Specific aims were to: 1) to examine the implementation planning and the PRISM contextual factors that affect the likelihood of RED implementation; 2) to assess the extent to which the actual implementation of RED adheres to the 11 components and 12 Toolkit implementation steps recommended by the developers of RED and, if modified, the ways in which these components and/or implementation steps were tailored; and 3) to identify, in the eight VAMCs, the factors affecting RED implementation.
Role: Co-Investigator

RRP 11-022 (Shin) VA HSR&D QUERI 10/2011-3/2013
How to Interpret and Use Patient Safety Indicator Reports
The overall goal of this pilot study is to enhance knowledge and use of the PSIs for quality improvement by developing and implementing an intervention that will educate VA stakeholders on the PSIs. This intervention will include dissemination of facility-specific PSI reports and implementation of a series of cyber seminars to increase awareness of PSIs.
Role: Principal Investigator

XVA 68-010 (Sullivan) VA Office of GEC 10/2010-9/2013
Evaluation of the Patient-Centric Alternatives to Institutional Extended Care Pilot Grants
The major goal of this project is to evaluate the 59 funded projects under the Patient-Centric Alternatives to Institutional Extended Care grants which are intended to increase the options for transformational and innovative alternatives to institutional extended care available to Veterans by supporting innovative clinical demonstration pilots that hold promise for efficacy, sustained success, and subsequent dissemination and are integrated and coordinated with other patient-centered initiatives.
Role: Co-Investigator

SDR 07-002 (Rosen) VA HSR&D 10/2007-3/2012
Validating the Patient Safety Indicators in the VA: A Multifaceted Approach
The purpose of this study is to develop a valid and reliable set of VA specific patient safety measures. We will examine several aspects of AHRQ's Patient Safety Indicators' validity that have not yet been studied. These include: criterion validity, attribution validity and utility validity.
Role: Project Manager

OMB No. 0925-0001 and 0925-0002 (Rev. 03/2020 Approved Through 02/28/2023)

BIOGRAPHICAL SKETCH

Provide the following information for the Senior/key personnel and other significant contributors.
Follow this format for each person. **DO NOT EXCEED FIVE PAGES.**

NAME: Engle, Ryann L

eRA COMMONS USER NAME (credential, e.g., agency login):

POSITION TITLE: Research Health Scientist

EDUCATION/TRAINING *(Begin with baccalaureate or other initial professional education, such as nursing, include postdoctoral training and residency training if applicable. Add/delete rows as necessary.)*

INSTITUTION AND LOCATION	DEGREE *(if applicable)*	Completion Date MM/YYYY	FIELD OF STUDY
Miami University	BA	2001	Zoology
Boston University	MPH	2003	Health Services

A. Personal Statement

I am excited to serve as a Co-investigator on Drs. Mull and Branch-Elliman's VA HSR&D proposal "Assessing the Sustainability of Compliance with Surgical Site infection Prophylaxis After Discontinuation of Mandatory Active Reporting." I am a Research Health Scientist at VA Boston Healthcare Center, in the Center for Healthcare Organization and Implementation Research (CHOIR). At CHOIR I have 14 years of experience leading and contributing to the qualitative analysis of numerous projects, including those evaluating organizational processes affecting patient care. My main research foci include organizational behavior and transformation (largely focusing on middle managers), implementation research, and Long Term Support Services (LTSS), both institutional and non-institutional. Outside of my qualitative expertise I am trained in implementation facilitation via the Behavioral Health QUERI and lean process improvement (VA Green Belt). Additionally, I have been heavily involved with the Veterans Engagement in Research Group (VERG) at CHOIR, specifically as the original chair of the CHOIR VERG Stakeholder Council and currently as the co-chair of the VERG Dissemination subcommittee, which focuses on engaging Veterans in our research and ensuring Veteran stakeholders learn about CHOIR research activities and findings. My qualitative expertise, research foci, and additional training are relevant to the goals of the proposed project.

1. Jasuja GK, **Engle RL**, Skolnik A, Rose AJ, Male A, Reisman JI, Bokhour BG. *Understanding the Context of High-and Low-Testosterone Prescribing Facilities in the Veterans Health Administration (VHA): a Qualitative Study.* Journal of general internal medicine. 2019 Sep 11:1-8.
2. **Engle RL**, Mohr DC, Holmes SK, Seibert MN, Afable M, Leyson J, Meterko M. *Evidence-based practice and patient-centered care: Doing both well.* Health care management review. 2019 Jun 20.
3. **Engle RL**, Lopez ER, Gormley KE, Chan JA, Charns MP, Lukas CV. *What roles do middle managers play in implementation of innovative practices?* Health Care Manage Rev. 2017 Jan/Mar; 42(1):14-27.
4. Gören JL, Rose AJ, **Engle RL**, Smith EG, Christopher ML, Rickles NM, Semla TP, McCullough MB. *Organizational characteristics of Veterans Affairs clinics with high and low utilization of clozapine.* Psychiatric Services. 2016 Jun 15;67(11):1189-96.

B. Positions and Honors

1998-2000 Student Aide, Department of Zoology, Miami University, Oxford, OH
2000-2001 Research Assistant, Department of Zoology, Miami University, Oxford, OH

2002-2005 Research Coordinator, Health Services Department, Boston University, Boston, MA
2005-2006 Project Manager, Health Services Department, Boston University, Boston, MA
2006-2011 Health Science Specialist, Center for Healthcare Organization and Implementation Research (formerly Center for Organization Leadership and Management Research) Department of Veterans Affairs, VA Boston Healthcare System, Boston, MA
2011-2019 Research Health Scientist - Senior Project Manager Center for Healthcare Organization and Implementation Research Department of Veterans Affairs, VA Boston Healthcare System, Boston, MA
2019- Research Health Scientist - Co-Investigator Center for Healthcare Organization and Implementation Research Department of Veterans Affairs, VA Boston Healthcare System, Boston, MA

Other Experience and Professional Memberships
AcademyHealth
Academy of Management

Honors
2000 Howard Hughes Medical Institute Summer Research Internship
2003 Outstanding Field Practice Placement Award
2007 VA Superior Performance Award
2015 Best Abstract presented at AcademyHealth Annual Research Meeting; Minneapolis, MN.
2015 Organizational Behavior and Management, Best Paper presented at Academy of Management Annual Meeting; Vancouver, BC, Canada
2018 APA Division 18, Best Paper of the Year, Psychological Services
2019 Department of Veteran Affairs, Office of Geriatrics and Extended Care, Certificate of Appreciation

C. Contributions to Science

Organizational Research
Organizational level factors play a key role in both process and outcomes; therefore, it is imperative to understand those factors as they relate to a healthcare setting. Through my work in both VA Medical Centers and LTSS settings I have contributed to the understanding how organizational level factors influence process and outcomes for both staff and patients. In the following contributions I was involved with the collection and analysis of qualitative data.

1. Jasuja GK, **Engle RL**, Skolnik A, Rose AJ, Male A, Reisman JI, Bokhour BG. *Understanding the Context of High-and Low-Testosterone Prescribing Facilities in the Veterans Health Administration (VHA): a Qualitative Study.* Journal of general internal medicine. 2019 Sep 11:1-8.

2. **Engle RL**, Mohr DC, Holmes SK, Seibert MN, Afable M, Leyson J, Meterko M. *Evidence-based practice and patient-centered care: Doing both well.* Health care management review. 2019 Jun 20.

3. **Engle RL**, Lopez ER, Gormley KE, Chan JA, Charns MP, Lukas CV. *What roles do middle managers play in implementation of innovative practices?* Health care management review. 2017 Jan/Mar; 42(1):14-27.

4. Gören JL, Rose AJ, **Engle RL**, Smith EG, Christopher ML, Rickles NM, Semla TP, McCullough MB. *Organizational characteristics of Veterans Affairs clinics with high and low utilization of clozapine.* Psychiatric Services. 2016 Jun 15;67(11):1189-96.

Implementation Research
Implementation of innovation in an organizational setting is a complex endeavor. Through my work in both the VA Medical Centers and long-term care settings I have contributed to a better understanding of the resources,

structures and processes needed in order to implement new innovations. In the following contributions I was involved with the implementation of new innovations, as well as the collection and analysis of qualitative data related to that innovation.

1. Sullivan JL, Shin MH, **Engle RL**, Yaksic E, VanDeusen Lukas C, Paasche-Orlow MK, Starr LM, Restuccia JD, Holmes SK, Rosen AK. *Evaluating the Implementation of Project Re-Engineered Discharge (RED) in Five Veterans Health Administration (VHA) Hospitals*. Jt Comm J Qual Patient Saf. 2018 Nov;44(11):663-673.

2. **Engle RL**, Evans L, Radwin L, Factor A, Charns MP, Vashi A. *The Impact of Senior Leader Instability on Implementation of Lean Management Systems*. Poster presented at AcademyHealth Annual Research Meeting; 2017 Jun 25; New Orleans, LA.

3. **Engle RL**, Rivard P, Shin M, Frigand C, Holmes SK, VanDeusen Lukas C , Shwartz M, and Singer S. *The Roles, Actions, and Experiences of Nurse Managers in Quality and Patient Safety Improvement Work*. Poster presented at AcademyHealth Annual Research Meeting; 2016 Jun 26; Boston, MA.
4. Sullivan JL, **Engle RL**, Adjognon O, Shin M, Afable M, White RA, Lukas CV. *Factors Affecting Implementation of a Patient-Centered Non-Institutional Long-Term Care Grant Program in the Veterans Health Administration*. Paper presented at: VA HSR&D National Meeting; 2015 Jul 19; Philadelphia, PA.

Long Term Support Services

Long Term Support Services (LTSS) encompass care and services provided along a continuum of settings. Through my work in both institutional and community-based supports and services I have contributed to the understanding of how both quality of care and person-centered care are delivered in a variety of settings, including home based primary care programs and nursing homes. In the following contributions I was involved with the collection and analysis of qualitative data.

1. Sullivan JL, Adjognon OL, **Engle RL**, Shin MH, Afable MK, Rudin W, White B, Shay K, Lukas CV. *Identifying and overcoming implementation challenges: Experience of 59 non-institutional long-term services and sports pilot programs in the Veterans Health Administration.* Health Care Management Review. 2018 Jul/Sept; 43(3): 193-205.

2. Parker V, **Engle RL**, Afable MK, Tyler DA, Gormley K, Stolzmann K, Shwartz M, Sullivan JL. *Staff-perceived Conflict between Resident-centered Care and Quality in the Skilled Nursing Facility: Are Both Possible?* Clin Gerontol. 2018 Apr 23:1-10.

3. Sullivan JL, **Engle RL**, Tyler D, Afable MK, Gormley K, Shwartz M, Adjognon O, Parker VA. *Is Variation in Resident-Centered Care and Quality Performance Related to Health System Factors in Veterans Health Administration Nursing Homes?* Inquiry. 2018 Jan-Dec; 55:46958018787031.

4. **Engle RL**, Tyler DA, Gormley KE, Afable MK, Curyto K, Adjognon OL, Parker VA, Sullivan JL. *Identifying barriers to culture change: A qualitative analysis of the obstacles to delivering resident-centered care*. Psychol Serv. 2017 Aug;14(3):316-326.

Complete List of Published Work in MyBibliography:
https://www.ncbi.nlm.nih.gov/myncbi/12S-a8kYw_yAG/bibliography/public/

D. Additional Information: Research Support and/or Scholastic Performance
<u>Ongoing Research Support</u>

<u>Completed Research Support</u>

VXA-11-50 (Simons) 1/1/2018-9/30/2018
VISN 2 Suicide Prevention COE/OMHSP

Examining Veterans Mental and Behavioral Care during care transitions from VA Community Living Centers
The goal of this study is to identify mental health needs of Veterans transitioning from 4 VA CLCs to the community. In particular, we will utilize chart review, a battery of quantitative measures, and qualitative interviews to understand mental health need before and after discharge.
Role: Co-Investigator

IIR 13-343 (Hartmann) 03/2016-02/2019
VA HSR&D
Safety climate in CLCs: variation, predictors, and impact on resident outcomes
The CLC Employee Survey of Attitudes about Resident Safety (CESARS) was developed to assess and help improve CLC safety climate. The CESARS consists of two parts: (1) a multi-domain CLC safety climate instrument (i.e., "CESARS Survey") and (2) a CLC safety climate toolkit (i.e., "CESARS Toolkit"). The current study builds on this prior work. The study has 3 aims. (1) Identify employee and organizational characteristics associated with safety climate variation across VHA and over time. (2) Examine the relationship of safety climate to both care processes and clinical outcomes across VHA and over time. (3) Pilot the use of the CESARS Toolkit.
Role: Qualitative Analyst

CDA 13-265 (Jasuja) 03/2016-02/2019
VA HSR&D
Optimizing Testosterone Prescribing in the VA
This CDA will first identify quantitative and qualitative patient-, provider-, and site-level correlates of sub-optimal testosterone use, and then use this information to inform the design of an intervention to improve testosterone prescribing in the VA. Examine patient, provider, and key opinion leader perceptions and system-level factors that relate to testosterone prescribing, using qualitative methods.
Role: Qualitative Analyst

IIR-11-358 (Dosa) 08/01/13-01/31/17
VA HSR&D
Training and Coaching to Promote High Performance in VA Community Living Centers
This study proposes a multi-level approach to test training interventions for direct-care workers and their supervisors in VA Community Living Centers (CLCs). We posit that successful translation of learning into practice happens when clinical knowledge is combined with communication skills and managerial support that enable exercising new knowledge. We propose testing a set of complementary training interventions to address knowledge, skills, and managerial support and assess the effect of these interventions on CLC employee and resident outcomes.
Role: Co-investigator

IIR 11-356 (Sullivan) 03/01/13-02/28/16
VA HSRD
Examining the Relationship of Culture Change, Adverse Events and Costs in CLCs
The specific objectives are to: 1) examine whether the level of implementing Person Centered Care (PCC) is associated with higher quality over time; 2) examine whether higher facility-level quality is associated with lower patient-level costs; and 3) identify key structural and organizational characteristics, and PCC implementation and quality processes that distinguish CLCs providing high PCC and quality from other CLCs where PCC and quality performance is lower.
Role: Co-Investigator

SDP 12-251 (Shwartz) 10/13-09/16
VA HSR&D QUERI
Leveraging Front-line Expertise in Quality and Safety (LFLE)
The primary aim of the proposed study is to conduct a LFLE intervention and assess implementation processes and factors associated with success, with the secondary aim of assessing LFLE effectiveness in VA. Specifically, the study objectives are: 1) to implement LFLE with fidelity in order to engage senior *managers*, gain their buy-in, participation and follow through; 2) to describe the implementation strategies in each site, including both study team and local site activities; 3) to evaluate the effects of the LFLE intervention

on quality and safety-related practices and organizational climate in intervention work areas; 4) to analyze organizational and process factors that affect LFLE implementation and sustainability.
Role: Co-Investigator

OMB No. 0925-0001 and 0925-0002 (Rev. 03/2020 Approved Through 02/28/2023)

BIOGRAPHICAL SKETCH

Provide the following information for the Senior/key personnel and other significant contributors.
Follow this format for each person. **DO NOT EXCEED FIVE PAGES.**

NAME: Elwy, Anashua Rani

eRA COMMONS USER NAME (credential, e.g., agency login): ARELWY

POSITION TITLE: Research Health Scientist, Center for Healthcare Organization and Implementation Research; Associate Professor, and Director, Implementation Science Core, Department of Psychiatry and Human Behavior, Warren Alpert Medical School, Brown University

EDUCATION/TRAINING *(Begin with baccalaureate or other initial professional education, such as nursing, include postdoctoral training and residency training if applicable. Add/delete rows as necessary.)*

INSTITUTION AND LOCATION	DEGREE *(if applicable)*	Completion Date MM/YYYY	FIELD OF STUDY
University of Michigan, Ann Arbor, MI	A. B.	05/1991	Psychology/Sociology
London School of Economics and Political Science, London, England	M.Sc.	07/1995	Social Psychology
King's College London, London, England	Ph.D.	09/2001	Health Psychology
Department of Veterans Affairs, Health Services Research & Development Service	Postdoctoral Fellowship	09/2004	Health Services Research

A. Personal Statement

I am very excited to be working with Drs. Hillary Mull and Westyn Branch-Elliman on their HSR&D IIR proposal *"Assessing the Sustainability of Compliance with Surgical Site Infection Prophylaxis After Discontinuation of Mandatory Active Reporting".* In this study, I will be providing the PIs and the team with guidance on using the Dynamic Sustainability Framework (DSF) over time, and to assist with identifying scale-up, spread and diffusion constructs throughout the project. As Dr. Branch-Elliman's long-time mentor in implementation science, it gives me great satisfaction and pride to see the development of such an important proposal focused on sustainability, a concept that Dr. David Chambers of the National Cancer Institute calls "an area ripe for exploration and study". I currently serve as a faculty member on the National Cancer Institute's Training Institute in Dissemination and Implementation Research in Cancer (TIDIRC). Our VA QUERI program, of which I am MPI, "Bridging the Care Continuum for Vulnerable Veterans across VA and Community Care", is also using the DSF to guide our work, and thus, my leadership in this QUERI program will be directly relevant to the HSR&D work of Drs. Mull and Branch-Elliman. My own implementation science pursuits focus on building stakeholder and leadership buy-in for national policy implementation across large integrated healthcare systems; evaluating and disseminating evidence-based complementary and integrative health therapies to treat PTSD, depression and chronic pain; social network analysis; and implementation outcome assessment.

1. Branch-Elliman W, Gupta K, **Elwy AR**. Factors influencing uptake of evidence-based antimicrobial prophylaxis guidelines for electrophysiology procedures. *American Journal of Infection Control*. 2019 Dec 2. pii: S0196-6553(19)30949-6. doi: 10.1016/j.ajic.2019.10.020. [Epub ahed of print]. PMID: 31806236

2. Bauer MS, Weaver K, Kim B, Miller C, Lew R, Stolzmann K, Sullivan JL, Riendeau R, Connolly S, Pitcock J, Ludvigsen SM, **Elwy AR**. The Collaborative Chronic Care Model for mental health conditions: from evidence synthesis to policy impact to scale-up and spread. *Medical Care.* 2019; 57 (Suppl. 10, Suppl. 3) S221-S227. PMID: 31517791

3. **Elwy AR**, Kim B, Plumb DN, Wang S, Gifford AL, Asch SM, Bormann JE, Mittman BS, Valente TW, Palinkas L. The connectedness of mental health providers referring patients to a treatment study for post-traumatic stress: a social network study. *Administration and Policy in Mental Health and Mental Health Services.* 24 June 2019, doi: 10.1007/s10488-019-00945-y [Epub ahead of print]. PMID: 31236732
4. **Elwy AR**, Wasan AD, Gillman AG, Johnston KL, Dodds N, McFarland C, Greco CM. Using Formative Evaluation Methods to Improve Clinical Implementation Efforts: Description and an Example. *Psychiatry Research.* 26 August 2019, doi:10.1016/j.psychres.2019.112532 [Epub ahead of print]. PMID: 31477261

B. Positions and Honors

Positions and Employment

1999-2001	Research Associate, Medical Research Council, University of Glasgow, UK
2001-2002	Consultant, World Health Organization, Management of Noncommunicable Diseases
2002-2004	Postdoctoral Fellow in Health Services Research, VA HSR&D
2004-2015	Investigator, HSR&D Center of Excellence, Bedford, MA
2004-2006	Research Associate, Boston University School of Public Health, Health Services Department
2006-2014	Assistant Professor, Boston University School of Public Health, Department of Health Policy and Management
2007-2008	Lecturer, Boston University School of Public Health, Maternal and Child Health Department
2011-2015	Co-Implementation Research Coordinator, HIV/Hepatitis Quality Enhancement Research Initiative (QUERI), Veterans Health Administration
2015-2016	Director (PI), HSR&D Center for Information Dissemination and Education Resources (CIDER), VA Boston Healthcare System
2015-2017	Director, MPH Certificate in Program Management, Boston University School of Public Health
2015-	Investigator, Center for Healthcare Organization and Implementation Research, VA Boston
2015-2018	Associate Professor, Boston University School of Public Health, Department of Health Policy and Management (now Adjunct Associate Professor)
2018-	Associate Professor, and Director, Implementation Science Core, Department of Psychiatry and Human Behavior, Warren Alpert Medical School, Brown University
2018-	Implementation Scientist, Biobehavioral Sciences Core, Providence/Boston Center for AIDS Research (CFAR)
2018-	Senior Investigator, The Mindfulness Center, Brown University School of Public Health
2020-	Associate Professor (secondary appointment), Behavioral and Social Sciences, Brown University School of Public Health

Other Experience and Professional Memberships

1998-2005	Member, British Psychological Society
1999-2005	Member, European Health Psychology Society
2001-	Member, American Psychological Association
2002-	Member, Society of Behavioral Medicine
2003-	Member, AcademyHealth
2016-	Founding Member, Society for Implementation Research Collaboration
2005-2009	Member, Scientific Advisory Committee, Deborah Munroe Noonan Memorial Fund, Boston, MA
2013-2018	FACA appointment, VA Health Services Research and Development Scientific Merit Review
2014	Guest Editor, Medical Care, Special Issue, Complementary and Integrative Medicine Use
2018	NIH Study Section, Accelerating Colorectal Cancer Screening and Follow-up Through Implementation Science (ACCSIS): The Moonshot Initiative, ZRG1 AARR-N(52)
2018	PCORI Study Section, Implementation of Effective Shared Decision-Making Approaches in Practice Settings
2019-2020	Scientific Program Committee, 16th International Congress of Behavioral Medicine
2019-2022	Abstract Topic Chair, Society of Behavioral Medicine annual meeting

Honors

1990	Alpha Kappa Delta Sociology Honor Society
1995	PhD Studentship, Down Syndrome Association, UK
2008	Excellence in Teaching Award, Boston University School of Public Health (MC831,Spring)
2010	Excellence in Teaching Award, Boston University School of Public Health (PM755, Spring)
2011	Excellence in Teaching Award, Boston University School of Public Health (PM755, Fall)
2012-14	Implementation Research Institute Fellowship, NIMH-funded R25 MH080916-01A2
2014	Certificate of Appreciation from the VA Principal Deputy Under Secretary for Health
2014	Excellence in Teaching Award, Boston University School of Public Health (PM755, Spring)
2016	Selected for Mid-Career Faculty Leadership Program, Boston University Medical Campus
2017	Best Research Paper Award, VA Health Services Research & Development Service

C. Contributions to Science

1. We have explored the strong and persistent relationship between patients' perceptions of their illnesses and symptoms and their treatment-seeking behavior. This is the work that led to us exploring the role of complementary and integrative health treatments as a way of potentially modifying this treatment seeking behavior.

a. **Elwy AR**, Glickman ME, Bokhour BG, Dell NS, Mueller NM, Zhao S, Osei-Bonsu PE, Rodrigues S, Coldwell CM, Ngo T, Schlosser J, Vielhauer MJ, Pirraglia PA, Eisen SV. Using mixed methods to examine the role of Veterans' illness perceptions on depression treatment utilization and HEDIS concordance. *Medical Care*. 2016; 54 (6):e35-42. PMID: 24374425

b. Osei-Bonsu PE, Bokhour BG, Glickman ME, Rodrigues S, Mueller NM, Dell NS, Zhao S, Eisen SV, **Elwy AR**. The role of coping in depression treatment utilization for VA primary care patients. *Patient Education and Counseling.* 2014 94(3):396-402. PMID: 24315160

c. **Elwy AR**, Johnston JM, Bormann JE, Hull A, Taylor SL. A systematic scoping review of complementary and alternative medicine mind and body practices to improve the health of veterans and military personnel. *Medical Care.* 2014; 52 (Suppl 5):S70-82. PMID: 25397827

d. Bormann JE, Thorp SR, Smith EG, Glickman M, Beck D, Plumb DN, Zhao S, Osei-Bonsu PE, Hepner P, Rodgers C, Herz LR, **Elwy AR.** Individual treatment of posttraumatic stress disorder using mantram repetition: a randomized clinical trial. *American Journal of Psychiatry.* 2018; 175(10):979-988. PMID: 29921143

2. In addition to the contributions above, I have also explored, with a team of collaborators, the role that resilience can play in preventing or attaining treatment for a range of mental health disorders. Resilience, conceptualized by our team of consisting of increased self-efficacy, hardiness and social support, can act as a buffer to the development of mental health or substance abuse disorders following exposure to traumatic situations. I have also explored how physicians' self-efficacy about communicating with patients about substance use can impact patients' health outcomes.

a. Eisen SV, Osei-Bonsu PE, Glickman ME, Vogt D, Schultz MR, Martin JA, Drainoni ML, **Elwy AR**. Postdeployment resilience as a predictor of subsequent mental health in Veterans returning from Iraq and Afghanistan. *American Journal of Preventive Medicine*. 2014; 47(6):754-61. PMID: 25455117

b. **Elwy AR**, Horton N, Saitz R. Physicians' attitudes toward unhealthy alcohol and other drug use and self-efficacy for screening and counseling: association with counseling and patients' drinking outcomes. *Substance Abuse Treatment, Prevention and Policy* 2013 8:17. PMID: 23718191

c. Eisen SV, Schultz MR, Vogt D, Glickman ME, **Elwy AR**, Drainoni M, Osei-Bonsu P, Martin J. Mental and physical health status, alcohol and drug use following return from deployment to Iraq or Afghanistan. *American Journal of Public Health* 2012: 102 Suppl 1: S66-S73. PMID: 22390605

d. **Elwy AR**, Ranganathan G, Eisen SV. Race/Ethnicity and diagnosis as predictors of outpatient utilization among treatment initiators. *Psychiatric Services*. 2008; 59:1285-1291. PMID: 18971404

3. I have also explored the role that communication plays between doctors, patients, family members and other people within the healthcare system. Much of this work has focused on what it is like to deliver bad news to patients. But this work has also emphasized the need to focus on creating relationships with stakeholders throughout the healthcare system in order to best care for patients when things do go wrong with their care. Much of this work is aligned with the National Academy of Medicine's goals of becoming a Learning Health Care System.

a. **Elwy AR**, Itani K, Bokhour BG, Mueller NM, Glickman ME, Zhao S, Rosen AK, Brotschi EA, Sanchez V, Perkal M, Lynge D, Gallagher TH. Surgeons' disclosures of clinical adverse events. *JAMA Surgery.* 2016;151(11):1015-1021. PMID: 27438083

b. **Elwy AR**, Bokhour BG, Maguire EM, Wagner TH, Asch SM, Gifford AL, Gallagher TH, Durfee JM, Martinello RA, Schiffner S, Jesse RL. Improving healthcare systems' large scale adverse event disclosures: A Department of Veterans Affairs leadership, policymaker, research and stakeholder partnership**.** *Journal of General Internal Medicine.* 2014; 29 (Suppl 4):895-903. PMID: 25355090

c. Wagner TH, Taylor TT, Cowgill E, Asch SM, Su P, Bokhour BG, Durfee JM, Martinello RA, Maguire EM, **Elwy AR**. Intended and unintended effects of large-scale adverse event disclosure: a controlled before-after analysis of five large-scale notifications. *BMJ Quality and Safety.*2015; 24 (5):295-302. PMID: 25882785

d. **Elwy AR**, Yeh J, Worcester J, Eisen SV. An illness perception model of primary care patients' help seeking for depression. *Qualitative Health Research*. 2011; 21 (11):1495-1507. PMID: 21715607

Complete List of Published Works in MyBibliography:
https://www.ncbi.nlm.nih.gov/sites/myncbi/1lm424uxT9f5b/bibliography/47318773/public/?sort=date&direction=ascending

D. Additional Information: Research Support and/or Scholastic Performance

Ongoing Research Support

VA QUE 20-017 Elwy/McInnes/Midboe/Smelson (MPI) 6/1/20-5/31/25
Bridging the Care Continuum for Vulnerable Veterans across VA and Community Care (Bridge CC)
An implementation science program to improve health outcomes associated with MH and SUD by expanding access to and engagement in overdose prevention, treatment, and supportive services for Veterans most impacted by social determinants of health.
Role: MPI

NCCIH 1 U01 AT010462-01A1 Vranceanu (PI) 5/1/20-4/30/24
Toolkit for Optimal Recovery after Orthopedic Injury: a multi-site feasibility study to prevent persistent pain and disability
To determine whether the virtual TOR delivered at 1-2 months after injury is associated with more improvement in pain and physical function, as well as catastrophic thinking about pain, pain anxiety, depressive symptoms and post traumatic symptoms.
Role: Site PI

PCORI DI-2017C2-7558 Greco/Wasan (MPI) 7/1/18-6/30/20
Implementing Contextual Factors Assessment in Clinical and Research Settings
Leading the dissemination and implementation of the Healing Encounters and Attitudes List (HEAL) Context Factors assessment using the CHOIR and PROMIS systems in the University of Pittsburgh Medical Center health system.
Role: Site PI

VA QUERI PEC 16-354 Taylor/Zeliadt (MPI) 10/1/16-9/30/20
Complimentary and Integrative Health Evaluation Center (CIHEC)
Co-leading a center dedicated to implementing and evaluating CIH evidence based practices throughout VA.
Role: Co-I (formerly Co-PI)

VA HSR&D SDR 17-306 Taylor/Zeliadt (MPI) 1/1/18-12/31/24
The Assessing Pain, Patient Reported Outcomes and Complementary Health (APPROACH) Study" (CIH for Pain in VA: National Demonstration Study)
A two-year planning grant and four-year pragmatic trial comparing 3 arms of CIH services with 18,000 patients to examine these non-pharmacological approaches to pain management.
Role: Co-I

NIMH R34 MH113598-01A1 Wolff (PI) 7/1/18-6/30/21
Integrating Computer-Assisted Parent Training Therapy into Community Mental Health Clinical Practice.
An open trial and process evaluation of the Parenting Wisely intervention.
Role: Co-I

NIDA 1R01 DA045396-01A1 Spirito (PI) 7/1/18-6/30/22
Brief Individual and Parent Interventions for Marijuana Misuse in Truant Adolescents
A hybrid 1 effectiveness-implementation randomized controlled trial, examining barriers and facilitators among many stakeholders prior to future scale-up and spread of the GOALS intervention.
Role: Co-I

CDC 1 U18DP006429-01-00 Jelalian (PI) 4/1/19-3/31/24
Packaging and Disseminating the JOIN for ME Program in Low-Income Settings
An implementation-focused formative and process evaluation hybrid 1 study to develop fidelity-consistent adaptations to an existing evidence-based pediatric weight loss program for housing development and primary care settings
Role: Co-I

VA QUERI PEC 18-204 Rosen (PI) 4/1/19-3/31/21
Evaluating the Implementation of Patient Safety Practices to Ensure Timely, High-Quality Community Care for Veterans
Partnering with the National Center for Patient Safety to evaluate the Joint Patient Safety Reporting tool in VA for capturing adverse events which occur through non-VA care
Role: Co-I

Completed Research Support (of over 20 completed studies)
NIDA 5R01DA045396 Spirito (PI) 8/1/19-4/30/20
HEAL Supplement to Brief Individual and Parent Interventions for Marijuana Misuse in Truant Adolescents
Role: Co-I

VA HSR&D SDR 11-440 (and VA 10NC funded) Elwy (PI) 6/1/12-09/30/19
Veteran and Staff Perceptions of VHA Large Scale Adverse Event Communications
A series of four complementary studies examined past disclosures of potential risk of HIV/HCV infection to Veterans exposed during routine care. Developed a toolkit to improve communication of large scale events throughout the VA, which is implemented throughout VA through the creation of the Disclosure Support Program, funded by VA clinical operations (10NC).
Role: PI

VA QUERI 15-289 Kirchner/Bauer (PI) 10/1/15-9/30/19
Team Based Behavioral Health QUERI Program
A series of implementation and improvement studies to bring evidence to the formation of team based within general mental health and primary care services in VA.
Role: Co-I

NCCIH/NIH 1R01AT006466-01 Park (PI) 9/29/10-8/31/16
Development of a Translational Tool to Study Yoga Therapy
This study will systematically develop and validate a tool to assess the dimensions of yoga therapy that can be used in future research across patient populations and health problems.
Role: Site PI

OMB No. 0925-0001 and 0925-0002 (Rev. 03/2020 Approved Through 02/28/2023)

BIOGRAPHICAL SKETCH

Provide the following information for the Senior/key personnel and other significant contributors.
Follow this format for each person. **DO NOT EXCEED FIVE PAGES.**

NAME: **Mary T Hawn, MD, MPH**

eRA COMMONS USER NAME (credential, e.g., agency login): **MARYHAWN**

POSITION TITLE: **Professor and Chair of Surgery, Stanford University**

EDUCATION/TRAINING *(Begin with baccalaureate or other initial professional education, such as nursing, include postdoctoral training and residency training if applicable. Add/delete rows as necessary.)*

INSTITUTION AND LOCATION	DEGREE *(if applicable)*	Completion Date MM/YYYY	FIELD OF STUDY
University of Michigan, Ann Arbor, MI	B.S.	05/87	Biomedical Sciences
University of Michigan, Ann Arbor, MI	M.D.	05/91	Medicine
University of Michigan, Ann Arbor, MI	M.P.H.	05/96	Epidemiology

A. Personal Statement

I am a health services researcher focusing on surgical quality measurement and policy. My background and training in epidemiology coupled with my extensive research program in surgical quality measurement, particularly in the area of surgical infections uniquely position me to serve as a clinical collaborator for Drs. Mull and Branch-Elliman's proposal. My expertise and extensive experience in evaluation of surgical quality process and outcome linkage has impacted national policy and changed guidelines. Under my lead, we performed a comprehensive evaluation of the Surgical Care Improvement Project (SCIP) implementation using national VA data. We observed that the SCIP metrics had been rapidly adopted into practice, but did not find evidence that these process measures had a meaningful impact on patient outcomes or secular trends in surgical complications. We performed several secondary analyses illustrating the shortcomings of these process measures providing a comprehensive overview of the complexity of surgical care and factors that impact outcomes. The SCIP measures have since been retired. Dr. Mull's proposal is an important follow up to these studies to ensure that the advances we achieved with optimal perioperative antibiotic usage have been maintained despite the performance measures no longer being collected. The innovation of directly abstracting 'performance' data rather than relying on chart review will also provide important insight into the potential automation of surgical quality metrics. I also have experience with risk prediction modeling for surgical patients. Our Improving Surgical Quality: Reducing Readmissions study leveraged Big Data, in particular inpatient vital sign and laboratory data to develop prediction algorithms for readmission after surgery. I will provide my clinical expertise in developing the infection prediction tool described in aim 1 of her research proposal and provide contextual knowledge about the significance of potential missing data for aim 1. I have worked closely with Dr. Mull over the years and look forward to continued collaborations.

B. Positions and Honors

Positions and Employment

1998-2000 Assistant Professor, Dept of Surgery, Section of General Surgery, University of Michigan Medical School, Ann Arbor, Michigan
2001-2015 Staff Surgeon, Department of Veteran Affairs Medical Center, Birmingham, Alabama
2001-2006 Assistant Professor, Dept of Surgery, Section of Gastrointestinal Surgery, University of Alabama at Birmingham (UAB), Birmingham, AL
2006-2015 Chief, Section of Gastrointestinal Surgery, Dept of Surgery, UAB
2006-2011 Associate Professor, Dept of Surgery, Section of Gastrointestinal Surgery, UAB
2007-2012 Director, Center for Surgical, Medical and Acute care Research and Transitions (C-SMART),

	Birmingham VAMC HSR&D Research Enhancement Award Program (REAP)
2011-2015	Professor, Dept of Surgery, Section of Gastrointestinal Surgery, UAB
2013-2015	Vice Chair for Quality and Clinical Effectiveness, Department of Surgery, UAB
2013-2015	Chair, Surgical Quality Data Use Group, Veterans Health Administration National Surgery Office
2015-	Staff Surgeon, Palo Alto VAMC, Palo Alto, CA
2015-	Professor and Chair, Department of Surgery, Stanford University

Other Experience and Select Professional Memberships

American Board of Surgery, Director
American College of Surgeons, Chair of Scientific Forum Committee
American Surgical Association, Secretary
Association of VA Surgeons
Surgical Society of the Alimentary Tract, Board of Trustees, Treasurer

Honors

1986	American Gastroenterological Association Student Research Prize
1986	Phi Chi Freshman Anatomy Award
1990	Albert C. Furstenberg Otolaryngology Student of the Year
1991	C. Gardner Child Award for Excellence in General Surgery
1998	Administrative Chief Resident, Department of Surgery, University of Michigan
1997	Coller Society Clinical Tour, Frederick A. Coller Surgical Society
2008	Argus Award for excellence in teaching. University of Alabama SOM
2012	Alpha Omega Alpha Honor Society

C. Contributions to Science

1. **Understanding the Effectiveness of the Surgical Care Improvement Project.** The SCIP was implemented in 2006 with the goal of reducing surgical complications by 25% by 2010. Through our VA HSRD funded project, we linked VA SCIP performance data with VA surgical quality data. We found that despite rapid adoption of the measures, there was no association with adherence and surgical outcomes. Nor could we find evidence that complications decreased as a consequence of SCIP implementation. Based on ours and other's study, policy has changed and SCIP in no longer measured. We performed several additional analyses to identify other relevant factors associated with surgical outcomes.

a) Hawn, M.T., Itani, K.M., Gray, S.H., Vick, C.C., Henderson, W.G., Houston, T.K. (2008). Association of Timely Administration of Prophylactic Antibiotics for Major Surgical Procedures and Surgical Site Infection. Journal of the American College of Surgeons. 206(5):814-9. PMID: 18471703.
b) Ponce BA, Raines BT, Reed RD, Vick CC, Richman JS, Hawn MT. Surgical Site Infection After Arthroplasty: Comparative Effectiveness of Prophylactic Antibiotics: Do SCIP Guidelines Need to be Updated? J Bone Joint Surg Am. 2014 Jun 18;96(12):970-977.
c) Richman JS, Itani KM, Deierhoi RJ, Henderson WG, Hawn MT. Improved outcomes associated with a revised quality measure for continuing perioperative β-blockade. JAMA Surg. 2014 Oct;149(10):1031-7.
d) Hawn MT, Vick CC, Richman JR, Holman W, Deierhoi RJ, Graham LA, Henderson WG, Itani KMF. Surgical site infection prevention: Time to move beyond the Surgical Care Improvement Program. Ann Surg. 2011 Sep;254(3):494-501

2. **Risk prediction for readmission following major surgical procedures.** We combined VA surgical quality data with administrative, pharmacy, laboratory and vital sign data to develop readmission prediction models. We identified that the majority of the risk could be explained by pre-operative comorbidities, the type of operation and post-discharge events with little influence of events in the index hospitalization.

a) Morris MS, Graham LA, Richman JS, Hollis RH, Jones CE, Wahl T, Itani KM, Mull HJ, Rosen AK, Copeland L, Burns E, Telford G, Whittle J, Wilson M, Knight SJ, Hawn MT. Postoperative 30-day

Readmission: Time to Focus on What Happens Outside the Hospital. Ann Surg. 2016 Oct;264(4):621-31

b) Hollis R, Hawn MT. Hospital Readmissions after Surgery: How Important are Hospital and Specialty Factors? J Am Coll Surg. 2017 Apr;224(4):515-523
c) Copeland LA, Graham LA, Richman JS, Rosen AK, Mull HJ, Burns EA, Whittle J, Itani KM, Hawn MT. A study to reduce readmissions after surgery in the Veterans Health Administration: design and methodology. BMC Health Serv Res. 2017 Mar 14;17(1):198.
d) Wahl TS, Graham LA, Hawn MT, Richman J, Hollis RH, Jones CE, Copeland LA, Burns EA, Itani KM, Morris MS. Association of the Modified Frailty Index With 30-Day Surgical Readmission. JAMA Surg. 2017 May 3. doi: 10.1001
e) Hernandez-Boussard T, Graham LA, Desai K, Wahl TS, Aucoin E, Richman JS, Morris MS, Itani KM, Telford GL, Hawn MT. The Fifth Vital Sign: Postoperative Pain Predicts 30-day Readmissions and Subsequent Emergency Department Visits. Ann Surg. 2017 Jun 27.

3. **Risk prediction for major complications and mortality following elective surgery.** Using statewide Surgical Quality Improvement data, we develop models for predicting major postoperative complications and mortality in an elective surgical cohort. The models performed well for mortality and overall complications, but did not discriminate well for venous thromboembolism or wound complications. We prospectively validated the models and compared with expert surgeon estimates. These models highlight the complexity of predicting complications and in general, pre-operative commodities explained the majority of the risk.

a) Richman JS, Hosokawa PW, Min SJ, Tomeh MG, Neumayer L, Campbell DA Jr, Henderson WG, Hawn MT. Toward prospective identification of high-risk surgical patients. Am Surg. 2012 Jul;78(7):755-60.
b) Glasgow RE, Hawn MT, Hosokawa P, Henderson WG, Min SJ, Richman JS, Tomeh MG, Campbell D, Neumayer LA. A Comparison of Risk Estimates for Postoperative Complications by Statistical Prediction Models vs. Experienced Surgeons. J Am Coll Surg 2014 Feb;218(2):237-45.
c) Norton WE, Hosokawa PW, Henderson WG, Volckmann ET, Pell J, Tomeh MG, Glasgow RE, Min SJ, Neumayer LA, Hawn MT. Acceptability of the decision support for safer surgery tool. Am J Surg. Jun;209(6):977-84.

4. **Understanding the risk of perioperative adverse cardiac events in patients with coronary stents.** Following the rapid adoption of drug eluting stents for coronary revascularization, reports of stent thrombosis and death in the perioperative period led to the publication of guidelines recommending delaying surgery for 1 year following DES. We performed the largest cohort study of patients with coronary stents undergoing noncardiac surgery to define the incidence and timing of surgery following stenting as well as drivers of adverse peri-operative cardiac events. We also reported that stent type was not a big driver of risk and as such the ACC/AHA guidelines have been revised to recognize that surgery can be performed safely after 6 months following DES implantation.

a) Hawn MT, Graham LA, Itani KMF, Henderson WG, Maddox T. Risk of Major Adverse Cardiac Events Following Noncardiac Surgery in Patients With Coronary Stents. JAMA 2013 310(14):1462-72.
b) Graham LA, Singletary BA, Richman JS, Maddox TM, Itani KM, Hawn MT. Improved adverse postoperative outcomes with revised American College of Cardiology/American Heart Association guidelines for patients with cardiac stents. JAMA Surg. 2014 Nov;149(11):1113-20.
c) Holcomb CN, Graham LA, Richman JS, Rhyne RR, Itani KM, Maddox TM, Hawn MT. The incremental risk of noncardiac surgery on adverse cardiac events following coronary stenting. J Am Coll Cardiol. 2014 Dec 30;64(25):2730-9.

D. Additional Information: Research Support and/or Scholastic Performance

Ongoing Research Support

IIR 16-216 (VHA HSR&D) Sox-Harris (PI) 01/01/2018-12/31/2020

Choosing Wisely: Barriers to De-Implementation, Patterns, and Costs of Low Value Preoperative Testing for Veterans Undergoing Low Risk Procedures.
This study aims to develop and validate a tool to improve informed consent and help prevent complications from TJA: a risk, benefit, and satisfaction calculator that will allow for easy, accurate estimation of the personalized risks and benefits for each candidate. Role: Co-Investigator

Completed Research Support

PP0-10-296 (VHA HSR&D) Hawn (PI) 01/01/2011-09/30/2012
Measuring Up: Associations between SCIP measures and Surgical Outcomes

IAB 06-038-2 (VHA OQP) Houston (PI) 01/01/2007-06/30/2010
Detailing Smoking-Attributable Risks for Post-Operative Morbidity: Planning for Policy and Clinical Interventions

IIR 03-247-1 (VHA HSR&D) Hawn (PI) 07/01/2004-12/31/2009
Mesh Repair for Ventral Hernia: Which Rate is Right?

SBIR: 1R44NR010653-1 (AHRQ) Tomeh (PI) 01/01/2007-12/31/2008
Decision Support for Safe Surgery (DS3)

IIR 09-347-2 (VHA HSR&D) Hawn (PI) 10/01/2010-09/30/2014
Cardiac Risk and Stent Effect on Adverse Perioperative Outcomes

VA HSR&D CDA Richman (PI) 10/1/10-9/30/2015
Predicting Surgical Outcomes with NSQIP and Clinical Monitoring Data Career Development Award
Role: Primary Mentor

IIR 12-358 (VHA HSR&D) Hawn (PI) 10/1/2014-12/31/2017
Improving Surgical Quality: Risks and Impact of Readmission

VHA HSR&D CDA Mull (PI) 10/01/2014-09/30/2018
Development of an Adverse Event Surveillance System for Outpatient Surgery
Role: Secondary Mentor

Rebecca Lamkin - Biosketch Not Required

Daniel Sturgeon - Biosketch Not Required

OMB No. 0925-0001 and 0925-0002 (Rev. 03/2020 Approved Through 02/28/2023)

BIOGRAPHICAL SKETCH

Provide the following information for the Senior/key personnel and other significant contributors.
Follow this format for each person. **DO NOT EXCEED FIVE PAGES.**

NAME: Colborn, Kathryn (**née Benton**)

eRA COMMONS USER NAME (credential, e.g., agency login): k.colborn

POSITION TITLE: Assistant Professor

EDUCATION/TRAINING *(Begin with baccalaureate or other initial professional education, such as nursing, include postdoctoral training and residency training if applicable. Add/delete rows as necessary.)*

INSTITUTION AND LOCATION	DEGREE *(if applicable)*	Completion Date MM/YYYY	FIELD OF STUDY
Colorado State University, Fort Collins, CO	BS	08/2003	Health and Exercise Science
Tulane University, New Orleans, LA	MSPH	05/2005	Biostatistics
University of California, Berkeley, Berkeley, CA	PhD	12/2013	Biostatistics
Institute for Disease Modeling, Bellevue, WA	Postdoctoral Fellow	04/2014	Statistics/Mathematical modeling

A. Personal Statement

I am an Assistant Professor in the Department of Surgery and am Research Director of the Surgical Outcomes and Applied Research (SOAR) Program in the Department of Surgery at the University of Colorado Anschutz Medical Campus. I co-direct SOAR with my colleague Dr. Robert Meguid, a cardiothoracic surgeon. In my role as SOAR co-director, I collaborate with investigators conducting surgical outcomes and health services research and mentor surgical faculty, residents and other graduate students. I also oversee two MS and one PhD biostatisticians who collaborate with surgeon faculty, residents and fellows. In my own research, I currently focus on development and validation of statistical methodologies for clinical prediction models. These lines of inquiry typically involve machine learning and high dimensional model selection. Recently, Dr. Branch-Elliman and I have collaborated on projects to develop novel tools for measuring healthcare-associated infections, including application of natural language processing for identification of catheter-associated urinary tract infections, which was accepted for presentation at Academy Health. I also lead the Data Informatics and Statistics Core of the Palliative Care Research Cooperative Group, a U2C funded by the NIH's National Institute of Nursing Research. I have received extramural funding for my own research and have collaborated on numerous extramural research grants. I have mentored 15 graduate students, one T-32 fellow and one career development awardee. My current and prior research allows me to serve as a biostatistical collaborator for Drs. Mull and Branch-Elliman's proposal. I have worked closely with Dr. Branch-Elliman on several projects focused on prevention and identification of post-operative infections over the years and look forward to continued collaborations.

1. Scott HF, **Colborn KL**, Sevick CJ, Bajaj L, Kissoon N, Deakyne Davies SJ, Kempe A. Development and Validation of a Predictive Model of the Risk of Pediatric Septic Shock Using Data Known at the Time of Hospital Arrival. J Pediatr. 2020 Feb;217:145-151.e6. PubMed PMID: 31733815; PubMed Central PMCID: PMC6980682.
2. Bronsert M, Singh AB, Henderson WG, Hammermeister K, Meguid RA, **Colborn KL**. Identification of postoperative complications using electronic health record data and machine learning. Am J Surg. 2019 Oct 9;PubMed PMID: 31635792.

3. **Colborn KL**, Bronsert M, Hammermeister K, Henderson WG, Singh AB, Meguid RA. Identification of urinary tract infections using electronic health record data. Am J Infect Control. 2019 Apr;47(4):371-375. PubMed PMID: 30522837;
4. **Colborn KL**, Bronsert M, Amioka E, Hammermeister K, Henderson WG, Meguid R. Identification of surgical site infections using electronic health record data. Am J Infect Control. 2018 Nov;46(11):1230-1235. PubMed PMID: 29907448;

B. Positions and Honors

Positions and Employment

2005 - 2005	Quantitative Epidemiologist, Centers for Disease Control and Prevention, Atlanta, GA
2006 - 2009	Senior Professional Research Assistant, University of Colorado Denver, Aurora, CO
2009 - 2009	Graduate Student Instructor, University of California, Berkeley, Department of Biostatistics, Berkeley, CA
2010 - 2010	Graduate Student Researcher, University of California, Berkeley, Department of Biostatistics, Berkeley, CA
2011 - 2013	Graduate Student Researcher, University of California, Berkeley, Department of Statistics, Berkeley, CA
2013 - 2014	Postdoctoral Associate, Intellectual Ventures Laboratory, Institute for Disease Modeling, Bellevue, WA
2014 - 2015	Biostatistician Consultant, Centers for Disease Control and Prevention, President's Emergency Plan for AIDS Relief, Maputo
2015 - 2017	Assistant Research Professor, University of Colorado Anschutz Medical Campus, Division of Health Care Policy and Research, Aurora, CO
2016 -	Director, Data Informatics and Statistics Core, Palliative Care Research Cooperative Group, Aurora, CO
2017 - 2019	Assistant Professor, Department of Biostatistics and Informatics, University of Colorado Anschutz Medical Campus, Aurora, CO
2019 -	Assistant Professor, Department of Surgery, University of Colorado Anschutz Medical Campus, Aurora, CO
2019 -	Research Director, Surgical Outcomes and Applied Research, Department of Surgery, University of Colorado Anschutz Medical Campus, Aurora, CO

Other Experience and Professional Memberships

2016 -	Member, American Statistical Association
2016 -	Member, American Society of Tropical Medicine and Hygiene
2017 -	Member, International Biometric Society

Honors

2005	Center for Bioenvironmental Research Award, Tulane University
2010 - 2013	Graduate Fellowship, University of California, Berkeley
2011 - 2013	Academic Achievement Stipend, University of California, Berkeley
2012	Keith and Mary Taylor Scholarship, University of California, Berkeley
2018 - 2020	NIH Loan Repayment Program, National Institutes of Health, NIAID

C. Contributions to Science

1. **Surgical Outcomes Research**. I have been engaged in and have led surgical outcomes research projects for the past several years. I have received both internal pilot funding and extramural research funding (AHRQ R03; PI Colborn) for this work. In September 2019, I accepted a position as Assistant Professor in the Department of Surgery to co-direct SOAR. In this role, I help surgeon investigators apply for extramural research funding and work collaboratively with them on various projects.

a. Bronsert M, Singh AB, Henderson WG, Hammermeister K, Meguid RA, **Colborn KL**. Identification of postoperative complications using electronic health record data and machine learning. Am J Surg. 2019 Oct 9; PubMed PMID: 31635792.
b. **Colborn KL**, Bronsert M, Hammermeister K, Henderson WG, Singh AB, Meguid RA. Identification of urinary tract infections using electronic health record data. Am J Infect Control. 2019 Apr;47(4):371-375. PubMed PMID: 30522837; PubMed Central PMCID: PMC6312639.
c. **Colborn KL**, Bronsert M, Amioka E, Hammermeister K, Henderson WG, Meguid R. Identification of surgical site infections using electronic health record data. Am J Infect Control. 2018 Nov;46(11):1230-1235. PubMed PMID: 29907448; PubMed Central PMCID: PMC6312639.
d. Alvord LA, Henderson WG, **Benton K**, Buchwald D. Surgical outcomes in American Indian veterans: a closer look. J Am Coll Surg. 2009 Jun;208(6):1085-92.e1. PubMed PMID: 19476896.

2. **Analysis of Large Healthcare Datasets**: Since joining the University of Colorado, I have been engaged in various research projects utilizing large healthcare datasets. In many of these projects, my knowledge of predictive analytics and machine learning have enhanced this research.

   a. Scott HF, **Colborn KL**, Sevick CJ, Bajaj L, Kissoon N, Deakyne Davies SJ, Kempe A. Development and Validation of a Predictive Model of the Risk of Pediatric Septic Shock Using Data Known at the Time of Hospital Arrival. J Pediatr. 2020 Feb;217:145-151.e6. PubMed PMID: 31733815; PubMed Central PMCID: PMC6980682.
   b. Tiwari P, **Colborn KL**, Smith DE, Xing F, Ghosh D, Rosenberg MA. Assessment of a Machine Learning Model Applied to Harmonized Electronic Health Record Data for the Prediction of Incident Atrial Fibrillation. JAMA Netw Open. 2020 Jan 3;3(1):e1919396. PubMed PMID: 31951272.
   c. **Colborn KL**, Helmkamp L, Bender BG, Kwan BM, Schilling LM, Sills MR. Colorado Asthma Toolkit Implementation Improves Some Process Measures of Asthma Care. J Am Board Fam Med. 2019 Jan-Feb;32(1):37-49. PubMed PMID: 30610140; PubMed Central PMCID: PMC6943943.
   d. Calcaterra SL, Scarbro S, Hull ML, Forber AD, Binswanger IA, **Colborn KL**. Prediction of Future Chronic Opioid Use Among Hospitalized Patients. J Gen Intern Med. 2018 Jun;33(6):898-905. PubMed PMID: 29404943; PubMed Central PMCID: PMC5975151.

3. **Palliative and End of Life Care**: For at least a decade, I have been involved in palliative care research. I have worked closely with Dr. Jean Kutner on this research. Dr. Kutner is the PI on a U2C grant from the NIH NINR (now in its third round of funding) that has established a large network of collaborators in palliative care called the Palliative Care Research Cooperative Group (PCRC). The PCRC facilitates multi-site clinical trials in palliative and end-of-life care, conducts trainings for junior researchers, consults on grant applications, awards pilots, and hosts a data repository. I am the Director of the PCRC's Data Informatics and Statistics Core (DISC). In this role, I consult on grant applications and oversee the data repository. I also participate as a mentor in the investigator trainings.

   a. Sable-Smith A, Arnett KR, Nowels MA, **Colborn K**, Lum HD, Nowels D. Interactions with the healthcare system influence advance care planning activities: results from a representative survey in 11 developed countries. Fam Pract. 2018 May 23;35(3):307-311. PubMed PMID: 29140508; PubMed Central PMCID: PMC5965096.
   b. Zerzan J**, Benton K**, Linnebur S, O'Bryant C, Kutner J. Variation in pain medication use in end-of-life care. J Palliat Med. 2010 May;13(5):501-4. PubMed PMID: 20420544.
   c. Kutner JS, Smith MC, Corbin L, Hemphill L, **Benton K**, Mellis BK, Beaty B, Felton S, Yamashita TE, Bryant LL, Fairclough DL. Massage therapy versus simple touch to improve pain and mood in patients with advanced cancer: a randomized trial. Ann Intern Med. 2008 Sep 16;149(6):369-79. PubMed PMID: 18794556; PubMed Central PMCID: PMC2631433.

4. **Malaria research**: For almost a decade, I have been involved in malaria research in various endemic countries, including Papua New Guinea, Zambia, Mozambique, and Uganda. My contributions to these projects have focused on data management, study design, statistical analysis, and dissemination of research findings. I have been PI on a Bill and Melinda Gates Foundation (BMGF) Grand Challenges Explorations grant where we developed methodology for a malaria early warning system for Mozambique. I

have also served as lead biostatistician and subcontract PI on a BMGF funded mass-drug-administration project in Uganda. During my doctoral studies at Berkeley, I worked on a longitudinal cohort study of Papua New Guinean children. The manuscript resulting from this work has been cited more than 100 times (Mueller, et. al, 2012 - nee Benton).

a. **Colborn KL**, Giorgi E, Monaghan AJ, Gudo E, Candrinho B, Marrufo TJ, Colborn JM. Spatio-temporal modelling of weekly malaria incidence in children under 5 for early epidemic detection in Mozambique. Sci Rep. 2018 Jun 18;8(1):9238. PubMed PMID: 29915366; PubMed Central PMCID: PMC6006329.
b. **Colborn KL**, Mueller I, Speed TP. Joint Modeling of Mixed <i>Plasmodium</i> Species Infections Using a Bivariate Poisson Lognormal Model. Am J Trop Med Hyg. 2018 Jan;98(1):71-76. PubMed PMID: 29182143; PubMed Central PMCID: PMC5928727.
c. Koepfli C, **Colborn KL**, Kiniboro B, Lin E, Speed TP, Siba PM, Felger I, Mueller I. A high force of plasmodium vivax blood-stage infection drives the rapid acquisition of immunity in papua new guinean children. PLoS Negl Trop Dis. 2013;7(9):e2403. PubMed PMID: 24040428; PubMed Central PMCID: PMC3764149.
d. Mueller I, Schoepflin S, Smith TA, **Benton KL**, Bretscher MT, Lin E, Kiniboro B, Zimmerman PA, Speed TP, Siba P, Felger I. Force of infection is key to understanding the epidemiology of Plasmodium falciparum malaria in Papua New Guinean children. Proc Natl Acad Sci U S A. 2012 Jun 19;109(25):10030-5. PubMed PMID: 22665809; PubMed Central PMCID: PMC3382533.

Complete List of Published Work in My Bibliography:
https://www.ncbi.nlm.nih.gov/myncbi/1nGbbI7JMLAAu/bibliography/public/

D. Additional Information: Research Support and/or Scholastic Performance

Ongoing Research Support

OPP1148566, Bill and Melinda Gates Foundation
Echodu (PI) 04/26/16-06/30/20
Operational research to accelerate and sustain malaria reduction in Uganda building on USAID/PMI investments
Objective: To drive down malaria prevalence in two communities in a high transmission area, and maintain it at a low level, using novel sequences of currently available control interventions.
Role: Consortium PI/Biostatistician

2U2CNR014637-06, NIH NINR
Kutner (PI) 07/01/18-06/30/23
Palliative Care Research Cooperative Group: Refinement and Expansion
A national cooperative for facilitating multi-site clinical trials in palliative care research. I direct the Data Informatics and Statics Core, which offers consulting services for grant submission, management of a large data repository, and training of junior investigators in quantitative and qualitative methods.
Role: Co-Investigator/**Core Director**

3U2CNR014637-07S1, NIH NINR
Kutner (PI) 07/01/2019-06/30/2020
Palliative Care Research Cooperative Group (PCRC): Refinement and Expansion (supplement)
This supplement permits the expansion of resources offered by the PCRC. Resources include additional study data and protocols added to our libraries that are specifically focused on Alzheimer’s disease and related dementias (ADRD).
Role: Co-Investigator/**Core Director**

61859687-135944, Stanford University
Aslakson (PI) 04/01/2019-03/31/2020

A Multi-Center Randomized Controlled Trial of Perioperative Palliative Care Surrounding Cancer Surgery for Patients and their Family Members (the PERIOP-PC trial)
This is a subaward for the PCRC's DISC to audit and provide feedback on the primary grant. We also assist in preparing data for the PCRC data repository.
Role: Key Personnel

1R01CA239588, NIH
Rochford (PI) 04/01/2019 – 3/31/2024
Environmental determinants of KSHV transmission in rural Uganda
In this proposal, we will capitalize on an ongoing substantive research program on KSHV and will embed the proposed work within a long-standing population-based cohort in rural Uganda, with a substantial body of existing data.
Role: Co-I/Biostatistician

Completed Research Support (selection of recent)

R03HS026019-01, AHRQ
Colborn (PI) 04/01/18-03/31/20
Identification of postoperative infections using electronic health record and administrative claims data
Using electronic health record data, we will develop a model for identifying postoperative infections using the NSQIP data. This model can be used to supplement NSQIP and allow for assessment of a larger population of patients.
Role: **PI**

U24NR014637, NIH NINR
Kutner (PI) 09/28/13-06/30/18
Refinement and Expansion of the Palliative Care Research Cooperative Group
A national cooperative for facilitating multi-site clinical trials in palliative care research. As Co-Investigator, I directed the Data Informatics and Statics Core.
Role: Co-Investigator

OPP1161891, Bill and Melinda Gates Foundation Grand Challenges Explorations
Colborn (PI) 11/01/16-04/29/18
Development of an automated early warning system for malaria
Using weekly case reports of malaria cases from 142 districts in Mozambique combined with remote sensing data, we are developing a model to predict malaria transmission fluctuations in Mozambique. This model will combine machine learning techniques with a spatio-temporal model.
Role: **PI**

Data Science to Patient Value Pilot, University of Colorado School of Medicine
Colborn (PI) 07/01/16-06/30/18
Identification of surgical site infections using machine learning
We developed a model for identifying surgical site infections using EHR data and machine learning.
Role: **PI**

HHSN2722013000151, NIH NIAID (contract)
El Sahly (PI) 01/23/17-02/22/20
Natural History and Prospective Cohort Study to Describe the Neurological, Neurodevelopmental and other Clinical Outcomes in Children after Zika Virus Infection in Rural Guatemala
Objective is to characterize the neurodevelopmental outcomes of infants with postnatally acquired ZIKV infection compared with infants who are negative for ZIKV Role: Biostatistician

Joseph Francis - Biosketch Not Required

Kelly Echevarria - Biosketch Not Required

Hillary Babcock - Biosketch Not Required

OTHER SUPPORT

Mull, Hillary

ACTIVE

IIR 18-034 Mull (PI) 4/1/2020-3/31/2024 4.2 calendar
VA HSR&D $1,145,670
How Can We Make Invasive Non-Surgical Procedures Safer? Using Big Data to Identify Adverse Events and Opportunities to Mitigate Harm
Role: Principal Investigator
This IIR addresses the VA's urgent need for targeted surveillance tools to detect adverse events and inform quality improvement initiatives in invasive non-surgical interventional cardiology, interventional radiology and gastrointestinal endoscopy procedures. The proposed work will test two hypothesized opportunities to update care guidelines or design quality initiatives to mitigate patient harm using surveillance data: whether the patient received inappropriate antibiotics and whether an anesthesiologist was involved in the procedure.

SDR 19-327 Garrido (PI) 2/15/2019 – 9/15/2023 3.0 calendar
VA HSR&D $379,286
Community Care/MISSION Act Virtual Research Network
Role: Co-Investigator
The purpose of this funding is to establish and supervise a Community Care and MISSION Act Virtual Research Network, providing leadership over the range of project activities associated with the Network and in support of individual projects supported by MISSION Act research funds.

20-004 Mull (PI) 2/2020 – 7/2020 1.2 calendar
VA CHOIR LIP $ 14,620
Association Between Diabetic Foot Ulcers and Invasive Systemic Infections in the Veterans Health Administration: Can Increased Treatment of Localized Infection Prevent Harm?
Role: Principal Investigator
This CHOIR project explores the clinical pathways associated with diabetic foot ulcer treatment and the relationship between localized infection and downstream hospitalizations and mortality from systemic infection.

PENDING

IIR 20-076 Branch-Elliman (PI) 2/2021-1/2025 2.4 calendar
VA HSR&D $1,198,820
Role: Co-Investigator
Promoting De-Implementation of Inappropriate Antimicrobial Use in Cardiac Device Procedures By Expanding Audit and Feedback
This IIR focuses on leveraging a quality monitoring system to promote de-implementation of ineffective antimicrobial use and on scaling and disseminating the electronic surveillance tool throughout the national VA healthcare system.

OVERLAP

In the event that the pending grants are funded, I will reduce my effort accordingly.

OTHER SUPPORT

Westyn Branch-Elliman, MD, MMSc

ACTIVE

K12 Branch Elliman (PI) 10/2018 to 9/2020 9 calendar months
NHLBI $ 260,000.00
Implementation of an Infection Prevention Program for Reducing Cardiac Device Infections Following Invasive Electrophysiology Procedures (NIH NHLBI 1K12HL138049-01).
Role: PI
The goals of this implementation science training project are to collect formative evaluations for implementation of a comprehensive prevention program in the electrophysiology laboratory and to design an implementation trial.

IIR 18-034 Mull (PI) 4/2020-3/2024 1.8 calendar months
VA HSR&D $1,143,215
How Can We Make Invasive Non-Surgical Procedures Safer? Using Big Data to Identify Adverse Events and Opportunities to Mitigate Harm
Role: Co-Investigator
This IIR addresses the VA's urgent need for targeted surveillance tools to detect adverse events and inform quality improvement initiatives in invasive non-surgical interventional cardiology, interventional radiology and gastrointestinal endoscopy procedures. The proposed work will test two hypothesized opportunities to update care guidelines or design quality initiatives to mitigate patient harm using surveillance data: whether the patient received inappropriate antibiotics and whether an anesthesiologist was involved in the procedure.

PENDING

IIR 20-076 Branch-Elliman (PI) 2/2021-1/2025 4.5 calendar months
VA HSR&D $1,198,820
Promoting De-Implementation of Inappropriate Antimicrobial Use in Cardiac Device Procedures By Expanding Audit and Feedback
This IIR focuses on leveraging a quality monitoring system to promote de-implementation of ineffective antimicrobial use and on scaling and disseminating the electronic surveillance tool throughout the national VA healthcare system.

1I01HX003191-01 **Branch-Elliman (M-PI)** 10/2020-3/2022 2.4 calendar months
VA HSR&D $199,150
Enhancing the Care Continuum for Veterans Who Inject Drugs Using Harm Reduction Approaches
This HSR&D innovations grant is designed to develop an implementation plan for integrating harm reduction strategies, including sterile syringe exchange programs, for Veterans who inject drugs. This proposal focuses on planning, feasibility, and developing a community of care. A future proposal will focus on testing different implementation strategies for integrating these services into VA care.

OVERLAP

In the event that the pending grants are funded, I will reduce my effort accordingly so that my grant-funded efforts are less than 90% FTE.

Other Support

Shin, Marlena, JD, MPH

ACTIVE

CIN-14-240 (Damschroder) 05/2019-9/2020 4.8 calendar months
VA HSR&D
HRO Evaluation
Role: Co-Investigator
Our specific aims are to: 1) identify and explore the use of metrics used to monitor progress and impacts of HRO and identify measurement gap; 2) use rapid qualitative analytic approaches to develop insights about strategies and their effects related to HRO Rollout; and 3) set the stage for a randomized program evaluation project.

SDR 17-306 (Taylor/ Zeliadt co-PI) 04/2018-3/2024 4.8 calendar months
VA HSR&D Merit Award
APPROACH: Assessing Pain, Patient Reported Outcomes and Complementary Health (A National VA Demonstration Project)
Role: Co-Investigator
A two-year planning grant and four-year pragmatic trial comparing 3 arms of CIH services with 18,000 patients to examine these non-pharmacological approaches to pain management.

PEC 18-204 (Rosen PI) 11/2018-4/2021 2.4 calendar months
VA HSR&D QUERI
Evaluating the Implementation of Patient Safety Practices to Ensure Timely, High-Quality Community Care for Veterans
Role: Co-Investigator
The expansion of Community Care (CC), and the increase in the numbers of Veterans using CC, make it critical for VHA to balance the need to improve access to care, and at the same time, ensure that the services that VHA purchases in the community are of high quality. As there is little known about the quality and safety of care that Veterans receive in the community, this timely start-up project will begin to close these knowledge gaps.

PENDING:

IIR 20-076 Branch-Elliman (PI) 2/2021-1/2025 2.4 calendar months
VA HSR&D
Role: Co-Investigator
Promoting De-Implementation of Inappropriate Antimicrobial Use in Cardiac Device Procedures By Expanding Audit and Feedback
This IIR focuses on leveraging a quality monitoring system to promote de-implementation of ineffective antimicrobial use and on scaling and disseminating the electronic surveillance tool throughout the national VA healthcare system.

OVERLAP
There are currently no budgetary, scientific, or administrative overlaps between the current funded studies and the current proposal.

OTHER SUPPORT

ENGLE, RYANN L.

ACTIVE

CONCERT (Hartmann & Snow) 09/01/16 – 09/30/20 4.8 Cal months
Office of Geriatrics and Extended Care Current Year: $1,200,000
VA CLC's Ongoing National Center for Enhancing Resources and Training (CONCERT)
CONCERT helps build key infrastructure to support Geriatrics and Extended Care's vision for system-wide improvement in staff engagement, Veteran experience, and person-centered care. It comprises a large, multi-disciplinary team based at CHOIR and the Tuscaloosa VA Medical Center.
Role: Program Staff

PENDING

OVERLAP

There are currently no budgetary, scientific, or administrative overlaps between the current funded studies and the proposal. The projects utilize different samples, as well as separate and distinct hypotheses and research designs.

Other Support

ELWY, AR.

ACTIVE:

NCCIH 1 U01 AT010462-01A1 5/01/2020-4/20/2024 0.6 calendar
(Vranceanu) $3,500,000
Toolkit for Optimal Recovery after Orthopedic Injury: a multi-site feasibility study to prevent persistent pain and disability
Role: Site PI

VA QUE 20-017 6/1/2020-5/31/2025 3.6 calendar
(Elwy/McInnes/Midboe/Smelson) $5,077,800
Bridging the Care Continuum for Vulnerable Veterans across VA and Community Care (Bridge CC)
Role: MPI

VA QUERI PEI 4/1/2017-9/30/2020 0.6 calendar
(Taylor/Zeliadt) $1,150,000
Complimentary and Integrative Health Evaluation Center (CIHEC)
Co-leading a center dedicated to implementing and evaluating CIH evidence based practices throughout VA.
Role: Co-I (formerly MPI)

VA HSR&D SDR 17-306 1/1/18-12/31/2024 2.4 calendar
(Taylor/Zeliadt) $5,082,604
The Assessing Pain, Patient Reported Outcomes and Complementary Health (APPROACH) Study (CIH for Pain in VA: National Demonstration Study)
Role: Co-I

PCORI DI-2017C2-7558 7/1/2018 - 6/30/2020 0.8 calendar
(Greco/Wasan) $173,438
Implementing Contextual Factors Assessment in Clinical and Research Settings
To implement HEAL assessments into clinical practice in pain management clinics to improve the care experiences of patients and their healthcare providers
Role: Co-I

NIMH R34 MH113598-01A1 7/25/2018-5/31/2021 0.6 calendar
(Wolff) $253,381
Integrating Computer-Assisted Parent Training Therapy into Community Mental Health Clinical Practice.
An open trial and process evaluation of the Parenting Wisely intervention.
Role: Co-I

NIDA 1R01 DA045396-01A1 7/15/2018-4/30/2022 0.8 calendar
(Spirito) $660,478
Brief Individual and Parent Interventions for Marijuana Misuse in Truant Adolescents
A hybrid 1 effectiveness-implementation randomized controlled trial, examining barriers and facilitators among many stakeholders prior to future scale-up and spread of the GOALS intervention.
Role: Co-I

CDC 1 U18DP006429-01-00 4/1/2019-3/31/2024 1.2 calendar
(Jelalian) $747,410
Packaging and Disseminating the JOIN for ME Program in Low-Income Settings
An implementation-focused formative and process evaluation hybrid 1 study to develop fidelity-consistent adaptations to an existing evidence-based pediatric weight loss program for housing development and primary care settings.
Role: Co-I

VA QUERI PEC 18-204 4/1/2019-3/31/2021 0.6 calendar
(Rosen) $299,088
Evaluating the Implementation of Patient Safety Practices to Ensure Timely, High-Quality Community Care for Veterans
Partnering with the National Center for Patient Safety to evaluate the Joint Patient Safety Reporting tool in VA for capturing adverse events which occur through non-VA care
Role: Co-I

PENDING
NIAAA 2 P01 AA019072-11 6/1/2020-5/31/2025 1.2 calendar
(Kahler) $7,480,035
Addressing alcohol misuse in HIV prevention and care: The Brown University Alcohol Research Center on HIV (ARCH)
Role: Co-I

NHLBI 1 UG3 HL154279-01 7/1/2020-6/30/2027 1.8 calendar
(Koinis-Mitchell/McQuaid/Canino) $8,926,632
Puerto Rico Asthma Integrated Response Program: PR-AIR
Role: Site PI

VA HSR&D IIR 20-076 01 2/1/2021-1/31/2025 1.2 calendar
(Branch-Elliman) $1,198,820
Promoting De-Implementation of Inappropriate Antimicrobial Use in Cardiac Device Procedures By Expanding Audit and Feedback
This IIR focuses on leveraging a quality monitoring system to promote de-implementation of ineffective antimicrobial use and on scaling and disseminating the electronic surveillance tool throughout the national VA healthcare system.
Role: Co-I

OVERLAP:

None

Dr. Elwy has a Memorandum of Understanding between Brown University, Providence, RI and the Edith Nourse Rogers Memorial Veterans Hospital in Bedford, MA, for a work week of 60 hours.

OTHER SUPPORT

Hawn, Mary T.

Active

Sox-Harris and Mudumbai (CO-PI) 10/1/2017-9/30/2020 0.6 Calendar
HX002314-01A1
Choosing Wisely: Barriers to De-Implementation, Patterns, and Costs of Low Value Preoperative Testing for Veterans Undergoing Low Risk Procedures

Completed Research Support

Hawn (PI) 10/01/2014-09/30/2017 1.8 Calendar
IIR 12-358-3
Improving Surgical Quality: Risks and Impact of Readmission

Hawn (PI) 10/01/2010-09/30/2014 1.8 Calendar
IIR 09-347-2
Cardiac Risk and Stent Effect on Adverse Perioperative Outcomes

Hawn (PI) 04/01/2004-09/30/2013 3.0 Calendar
REA 04-195 (VHA HSR&D)
Center for Surgical, Medical Acute Care Research & Transitions (C-SMART) an HSR&D Research Enhancement Award Program (REAP) at the Birmingham VA Medical Center

Hawn (PI) 01/01/2011-09/30/2012 1.2 Calendar
PP0-10-296 (VHA HSR&D)
Measuring Up: Associations between SCIP measures and Surgical Outcomes

Houston (PI) 01/01/2007-06/30/2010 0.6 Calendar
IAB 06-038-2 (VHA OQP)
Detailing Smoking-Attributable Risks for Post-Operative Morbidity: Planning for Policy and Clinical Interventions

Pending

None

Overlap

There is no scientific, administrative or budgetary overlap.

Rebecca Lamkin - Other Support Not Required

Daniel Sturgeon - Other Support Not Required

OTHER SUPPORT

COLBORN, KATHRYN L

Active

OPP1148566 (Echodu) 07/01/2019-06/30/2020 1.2 calendar
Pilgrim Africa $17,230 (subcontract only)
Operational research to accelerate and sustain malaria reduction in Uganda building on USAID/PMI investments
Objective: To drive down malaria prevalence in two communities in a high transmission area, and maintain it at a low level, using novel sequences of currently available control interventions. These include mass-drug-administration and indoor-residual-spraying.
Role: Consortium PI/Biostatistician

2U2CNR014637-06 (Kutner, Ritchie, Pollak) 07/01/2018-06/30/2023 0.96 calendar
NIH NINR $1,590,996
Palliative Care Research Cooperative Group (PCRC): Refinement and Expansion
A national cooperative for facilitating multi-site clinical trials in palliative care research. I direct the Data Informatics and Statics Core, which offers consulting services for grant submission, management of a large data repository, and training of junior investigators in quantitative and qualitative methods.
Role: Co-I/Core Director

3U2CNR014637-07S1 (Kutner) 07/01/2019-06/30/2020 0.6 calendar
NIH NINR $375,133
Palliative Care Research Cooperative Group (PCRC): Refinement and Expansion
This supplement permits the expansion of resources offered by the PCRC. Resources include additional study data and protocols added to our libraries that are specifically focused on Alzheimer's disease and related dementias (ADRD).

61859687-135944 (Aslakson) 04/01/2020-03/31/2021 0.6 calendar
Stanford University $11,977 (subcontract only)
A Multi-Center Randomized Controlled Trial of Perioperative Palliative Care Surrounding Cancer Surgery for Patients and their Family Members (the PERIOP-PC trial)
This is a subaward for the PCRC's DISC to audit and provide feedback on the primary grant. We also assist in preparing data for the PCRC data repository.

1R01CA239588 (Rochford) 04/01/2019 – 3/31/2024 0.6 calendar
NIH $440,722
Environmental determinants of KSHV transmission in rural Uganda
In this proposal, we will capitalise on an ongoing substantive research program on KSHV and will embed the proposed work within a long-standing population-based cohort in rural Uganda, with a substantial body of existing data. The proposed work will address the profound knowledge gap regarding factors that influence KSHV transmission.
Role: Co-I/Biostatistician

Pending

IIR 20-076 (Branch-Elliman) 02/01/2021-01/31/2025 1.8 calendar
VA HSR&D $37,916 (subcontract only)
Promoting De-Implementation of Inappropriate Antimicrobial Use in Cardiac Device Procedures By Expanding Audit and Feedback
This grant focuses on leveraging a quality monitoring system to promote de-implementation of ineffective antimicrobial use and on scaling and disseminating the electronic surveillance tool throughout the national VA healthcare system

1R01HS027417-01 (Colborn and Meguid) 09/01/2020-08/31/2025 3 calendar
AHRQ $398,727
Automated Surveillance of Postoperative Infections (ASPIN)
Infectious complications of surgery (surgical site infection, pneumonia, urinary tract infection, and sepsis) are common, occurring in about 8% of major surgical procedures, and are costly. Currently used surveillance techniques require extensive human participation, resulting in delays of at least six months before the data are available to care providers, and the data represent only a small sample of all operations. We propose to develop a machine learning-based, near real-time recurring audit and feedback system for postoperative infections.
Role: mPI

OVERLAP

In the event that the pending grants are funded, I will reduce my effort accordingly so that my grant-funded efforts are less than 90% FTE.

Joseph Francis - Other Support Not Required

Kelly Echevarria - Other Support Not Required

Hillary Babcock - Other Support Not Required

OMB Number: 4040-0010
Expiration Date: 12/31/2022

RESEARCH & RELATED BUDGET - SECTION A & B, Budget Period 1

ORGANIZATIONAL DUNS*: 0344322650000

Budget Type*: ● Project ❍ Subaward/Consortium

Enter name of Organization: VA BOSTON HEALTH CARE SYSTEM

Start Date*: 01-01-2021 **End Date*:** 12-31-2021 **Budget Period:** **1**

A. Senior/Key Person

	Prefix	First Name*	Middle Name	Last Name*	Suffix	Project Role*	Base Salary ($)	Calendar Months	Academic Months	Summer Months	Requested Salary ($)*	Fringe Benefits ($)*	Funds Requested ($)*
1 .		Hillary	Jane	Mull		PD/PI	120,888.00	3.0			30,222.00	12,146.00	42,368.00

Total Funds Requested for all Senior Key Persons in the attached file

Additional Senior Key Persons: File Name: **Total Senior/Key Person** **42,368.00**

B. Other Personnel

Number of Personnel*	Project Role*	Calendar Months	Academic Months	Summer Months	Requested Salary ($)*	Fringe Benefits*	Funds Requested ($)*
	Post Doctoral Associates						
	Graduate Students						
	Undergraduate Students						
	Secretarial/Clerical						
6	unique VA Personnel	27.6			160,614.00	53,038.00	213,652.00
**6**	**Total Number Other Personnel**					**Total Other Personnel**	**213,652.00**
						**Total Salary, Wages and Fringe Benefits (A+B)**	**256,020.00**

RESEARCH & RELATED Budget {A-B} (Funds Requested)

RESEARCH & RELATED BUDGET - SECTION C, D, & E, Budget Period 1

ORGANIZATIONAL DUNS*: 0344322650000

Budget Type*: ● Project ❍ Subaward/Consortium

Organization: VA BOSTON HEALTH CARE SYSTEM

Start Date*: 01-01-2021 **End Date*:** 12-31-2021 **Budget Period:** 1

C. Equipment Description

List items and dollar amount for each item exceeding $5,000

Equipment Item	Funds Requested ($)*
**Total funds requested for all equipment listed in the attached file**	
**Total Equipment**	**0.00**

Additional Equipment: File Name:

**D. Travel**	**Funds Requested ($)***
1. Domestic Travel Costs (Incl. Canada, Mexico, and U.S. Possessions)	
2. Foreign Travel Costs	
**Total Travel Cost**	**0.00**

**E. Participant/Trainee Support Costs**		**Funds Requested ($)***
1. Tuition/Fees/Health Insurance		
2. Stipends		
3. Travel		
4. Subsistence		
5. Other:		
**Number of Participants/Trainees**	**Total Participant Trainee Support Costs**	**0.00**

RESEARCH & RELATED Budget {C-E} (Funds Requested)

RESEARCH & RELATED BUDGET - SECTIONS F-K, Budget Period 1

ORGANIZATIONAL DUNS*: 0344322650000

Budget Type*: ● Project ❍ Subaward/Consortium

Organization: VA BOSTON HEALTH CARE SYSTEM

Start Date*: 01-01-2021 **End Date*:** 12-31-2021 **Budget Period:** 1

**F. Other Direct Costs**	**Funds Requested ($)***
1. Materials and Supplies	
2. Publication Costs	
3. Consultant Services	
4. ADP/Computer Services	
5. Subawards/Consortium/Contractual Costs	
6. Equipment or Facility Rental/User Fees	
7. Alterations and Renovations	
8 . Subtotal Costs (Other Direct, Other Sites)	9,944.00
**Total Other Direct Costs**	**9,944.00**

**G. Direct Costs**	**Funds Requested ($)***
**Total Direct Costs (A thru F)**	**265,964.00**

H. Indirect Costs

**Indirect Cost Type**	**Indirect Cost Rate (%)**	**Indirect Cost Base ($)**	**Funds Requested ($)***
		**Total Indirect Costs**	

Cognizant Federal Agency
(Agency Name, POC Name, and POC Phone Number)

**I. Total Direct and Indirect Costs**	**Funds Requested ($)***
**Total Direct and Indirect Institutional Costs (G + H)**	**265,964.00**

**J. Fee**	**Funds Requested ($)***

**K. Total Costs and Fee**	**Funds Requested ($)***
	**265,964.00**

L. Budget Justification* File Name: VA_Budget_Justification.pdf
(Only attach one file.)

RESEARCH & RELATED Budget {F-K} (Funds Requested)

OMB Number: 4040-0010
Expiration Date: 12/31/2022

RESEARCH & RELATED BUDGET - SECTION A & B, Budget Period 2

ORGANIZATIONAL DUNS*: 0344322650000

Budget Type*: ● Project ❍ Subaward/Consortium

Enter name of Organization: VA BOSTON HEALTH CARE SYSTEM

Start Date*: 01-01-2022 **End Date*:** 12-31-2022 **Budget Period:** **2**

A. Senior/Key Person

	Prefix	First Name*	Middle Name	Last Name*	Suffix	Project Role*	Base Salary ($)	Calendar Months	Academic Months	Summer Months	Requested Salary ($)*	Fringe Benefits ($)*	Funds Requested ($)*
1 .		Hillary	Jane	Mull		PD/PI	126,829.00	3.0			31,707.00	12,743.00	44,450.00

Total Funds Requested for all Senior Key Persons in the attached file

Additional Senior Key Persons: File Name: **Total Senior/Key Person** **44,450.00**

B. Other Personnel

Number of Personnel*	Project Role*	Calendar Months	Academic Months	Summer Months	Requested Salary ($)*	Fringe Benefits*	Funds Requested ($)*
	Post Doctoral Associates						
	Graduate Students						
	Undergraduate Students						
	Secretarial/Clerical						
6	unique VA Personnel	27.6			167,350.00	55,303.00	222,653.00
**6**	**Total Number Other Personnel**					**Total Other Personnel**	**222,653.00**
						**Total Salary, Wages and Fringe Benefits (A+B)**	**267,103.00**

RESEARCH & RELATED Budget {A-B} (Funds Requested)

RESEARCH & RELATED BUDGET - SECTION C, D, & E, Budget Period 2

ORGANIZATIONAL DUNS*: 0344322650000

Budget Type*: ● Project ❍ Subaward/Consortium

Organization: VA BOSTON HEALTH CARE SYSTEM

Start Date*: 01-01-2022 **End Date*:** 12-31-2022 **Budget Period:** 2

C. Equipment Description

List items and dollar amount for each item exceeding $5,000

Equipment Item	Funds Requested ($)*
**Total funds requested for all equipment listed in the attached file**	
**Total Equipment**	**0.00**

Additional Equipment: File Name:

D. Travel

D. Travel	Funds Requested ($)*
1. Domestic Travel Costs (Incl. Canada, Mexico, and U.S. Possessions)	
2. Foreign Travel Costs	
**Total Travel Cost**	**0.00**

E. Participant/Trainee Support Costs

E. Participant/Trainee Support Costs	Funds Requested ($)*
1. Tuition/Fees/Health Insurance	
2. Stipends	
3. Travel	
4. Subsistence	
5. Other:	
**Number of Participants/Trainees** **Total Participant Trainee Support Costs**	**0.00**

RESEARCH & RELATED Budget {C-E} (Funds Requested)

RESEARCH & RELATED BUDGET - SECTIONS F-K, Budget Period 2

ORGANIZATIONAL DUNS*: 0344322650000

Budget Type*: ● Project ❍ Subaward/Consortium

Organization: VA BOSTON HEALTH CARE SYSTEM

Start Date*: 01-01-2022 **End Date*:** 12-31-2022 **Budget Period:** 2

**F. Other Direct Costs**	**Funds Requested ($)***
1. Materials and Supplies	
2. Publication Costs	
3. Consultant Services	
4. ADP/Computer Services	
5. Subawards/Consortium/Contractual Costs	
6. Equipment or Facility Rental/User Fees	
7. Alterations and Renovations	
8 . Subtotal Costs (Other Direct, Other Sites)	37,731.00
**Total Other Direct Costs**	**37,731.00**

**G. Direct Costs**	**Funds Requested ($)***
**Total Direct Costs (A thru F)**	**304,834.00**

H. Indirect Costs

**Indirect Cost Type**	**Indirect Cost Rate (%)**	**Indirect Cost Base ($)**	**Funds Requested ($)***
		**Total Indirect Costs**	

Cognizant Federal Agency
(Agency Name, POC Name, and POC Phone Number)

**I. Total Direct and Indirect Costs**	**Funds Requested ($)***
**Total Direct and Indirect Institutional Costs (G + H)**	**304,834.00**

**J. Fee**	**Funds Requested ($)***

**K. Total Costs and Fee**	**Funds Requested ($)***
	**304,834.00**

L. Budget Justification* File Name: VA_Budget_Justification.pdf
(Only attach one file.)

RESEARCH & RELATED Budget {F-K} (Funds Requested)

OMB Number: 4040-0010
Expiration Date: 12/31/2022

RESEARCH & RELATED BUDGET - SECTION A & B, Budget Period 3

ORGANIZATIONAL DUNS*: 0344322650000

Budget Type*: ● Project ❍ Subaward/Consortium

Enter name of Organization: VA BOSTON HEALTH CARE SYSTEM

Start Date*: 01-01-2023 **End Date*:** 12-31-2023 **Budget Period:** 3

A. Senior/Key Person

	Prefix	First Name*	Middle Name	Last Name*	Suffix	Project Role*	Base Salary ($)	Calendar Months	Academic Months	Summer Months	Requested Salary ($)*	Fringe Benefits ($)*	Funds Requested ($)*
1 .		Hillary	Jane	Mull		PD/PI	129,366.00	3.0			32,341.00	12,998.00	45,339.00

Total Funds Requested for all Senior Key Persons in the attached file

Additional Senior Key Persons: File Name: **Total Senior/Key Person** **45,339.00**

B. Other Personnel

Number of Personnel*	Project Role*	Calendar Months	Academic Months	Summer Months	Requested Salary ($)*	Fringe Benefits*	Funds Requested ($)*
	Post Doctoral Associates						
	Graduate Students						
	Undergraduate Students						
	Secretarial/Clerical						
6	unique VA Personnel	27.6			174,553.00	57,566.00	232,119.00
**6**	**Total Number Other Personnel**					**Total Other Personnel**	**232,119.00**
						**Total Salary, Wages and Fringe Benefits (A+B)**	**277,458.00**

RESEARCH & RELATED Budget {A-B} (Funds Requested)

RESEARCH & RELATED BUDGET - SECTION C, D, & E, Budget Period 3

ORGANIZATIONAL DUNS*: 0344322650000

Budget Type*: ● Project ❍ Subaward/Consortium

Organization: VA BOSTON HEALTH CARE SYSTEM

Start Date*: 01-01-2023 **End Date*:** 12-31-2023 **Budget Period:** 3

C. Equipment Description

List items and dollar amount for each item exceeding $5,000

Equipment Item	Funds Requested ($)*
**Total funds requested for all equipment listed in the attached file**	
**Total Equipment**	**0.00**

Additional Equipment: File Name:

**D. Travel**	**Funds Requested ($)***
1. Domestic Travel Costs (Incl. Canada, Mexico, and U.S. Possessions)	
2. Foreign Travel Costs	
**Total Travel Cost**	**0.00**

**E. Participant/Trainee Support Costs**	**Funds Requested ($)***
1. Tuition/Fees/Health Insurance	
2. Stipends	
3. Travel	
4. Subsistence	
5. Other:	
**Number of Participants/Trainees** **Total Participant Trainee Support Costs**	**0.00**

RESEARCH & RELATED Budget {C-E} (Funds Requested)

RESEARCH & RELATED BUDGET - SECTIONS F-K, Budget Period 3

ORGANIZATIONAL DUNS*: 0344322650000

Budget Type*: ● Project ❍ Subaward/Consortium

Organization: VA BOSTON HEALTH CARE SYSTEM

Start Date*: 01-01-2023 **End Date*:** 12-31-2023 **Budget Period:** 3

F. Other Direct Costs	Funds Requested ($)*
1. Materials and Supplies	
2. Publication Costs	
3. Consultant Services	
4. ADP/Computer Services	
5. Subawards/Consortium/Contractual Costs	
6. Equipment or Facility Rental/User Fees	
7. Alterations and Renovations	
8 . Subtotal Costs (Other Direct, Other Sites)	32,053.00
**Total Other Direct Costs**	**32,053.00**

G. Direct Costs	Funds Requested ($)*
**Total Direct Costs (A thru F)**	**309,511.00**

H. Indirect Costs

Indirect Cost Type	Indirect Cost Rate (%)	Indirect Cost Base ($)	Funds Requested ($)*
		**Total Indirect Costs**	

Cognizant Federal Agency

(Agency Name, POC Name, and POC Phone Number)

I. Total Direct and Indirect Costs	Funds Requested ($)*
**Total Direct and Indirect Institutional Costs (G + H)**	**309,511.00**

J. Fee	Funds Requested ($)*

K. Total Costs and Fee	Funds Requested ($)*
	**309,511.00**

L. Budget Justification*	File Name: VA_Budget_Justification.pdf<br>(Only attach one file.)

RESEARCH & RELATED Budget {F-K} (Funds Requested)

OMB Number: 4040-0010
Expiration Date: 12/31/2022

RESEARCH & RELATED BUDGET - SECTION A & B, Budget Period 4

ORGANIZATIONAL DUNS*: 0344322650000

Budget Type*: ● Project ❍ Subaward/Consortium

Enter name of Organization: VA BOSTON HEALTH CARE SYSTEM

Start Date*: 01-01-2024 **End Date*:** 12-31-2024 **Budget Period:** 4

A. Senior/Key Person

	Prefix	First Name*	Middle Name	Last Name*	Suffix	Project Role*	Base Salary ($)	Calendar Months	Academic Months	Summer Months	Requested Salary ($)*	Fringe Benefits ($)*	Funds Requested ($)*
1 .		Hillary	Jane	Mull		PD/PI	131,953.00	3.0			32,988.00	13,258.00	46,246.00

Total Funds Requested for all Senior Key Persons in the attached file

Additional Senior Key Persons: File Name: **Total Senior/Key Person** **46,246.00**

B. Other Personnel

Number of Personnel*	Project Role*	Calendar Months	Academic Months	Summer Months	Requested Salary ($)*	Fringe Benefits*	Funds Requested ($)*
	Post Doctoral Associates						
	Graduate Students						
	Undergraduate Students						
	Secretarial/Clerical						
6	unique VA Personnel	27.6			179,931.00	59,388.00	239,319.00
**6**	**Total Number Other Personnel**					**Total Other Personnel**	**239,319.00**
						**Total Salary, Wages and Fringe Benefits (A+B)**	**285,565.00**

RESEARCH & RELATED Budget {A-B} (Funds Requested)

RESEARCH & RELATED BUDGET - SECTION C, D, & E, Budget Period 4

ORGANIZATIONAL DUNS*: 0344322650000

Budget Type*: ● Project ❍ Subaward/Consortium

Organization: VA BOSTON HEALTH CARE SYSTEM

Start Date*: 01-01-2024 **End Date*:** 12-31-2024 **Budget Period:** 4

C. Equipment Description

List items and dollar amount for each item exceeding $5,000

Equipment Item	Funds Requested ($)*
**Total funds requested for all equipment listed in the attached file**	
**Total Equipment**	**0.00**

Additional Equipment: File Name:

D. Travel	Funds Requested ($)*
1. Domestic Travel Costs (Incl. Canada, Mexico, and U.S. Possessions)	
2. Foreign Travel Costs	
**Total Travel Cost**	**0.00**

E. Participant/Trainee Support Costs		Funds Requested ($)*
1. Tuition/Fees/Health Insurance		
2. Stipends		
3. Travel		
4. Subsistence		
5. Other:		
**Number of Participants/Trainees**	**Total Participant Trainee Support Costs**	**0.00**

RESEARCH & RELATED Budget {C-E} (Funds Requested)

RESEARCH & RELATED BUDGET - SECTIONS F-K, Budget Period 4

ORGANIZATIONAL DUNS*: 0344322650000

Budget Type*: ● Project ❍ Subaward/Consortium

Organization: VA BOSTON HEALTH CARE SYSTEM

Start Date*: 01-01-2024 **End Date*:** 12-31-2024 **Budget Period:** 4

F. Other Direct Costs	Funds Requested ($)*
1. Materials and Supplies	
2. Publication Costs	
3. Consultant Services	
4. ADP/Computer Services	
5. Subawards/Consortium/Contractual Costs	
6. Equipment or Facility Rental/User Fees	
7. Alterations and Renovations	
8 . Subtotal Costs (Other Direct, Other Sites)	32,260.00
**Total Other Direct Costs**	**32,260.00**

G. Direct Costs	Funds Requested ($)*
**Total Direct Costs (A thru F)**	**317,825.00**

H. Indirect Costs

Indirect Cost Type	Indirect Cost Rate (%)	Indirect Cost Base ($)	Funds Requested ($)*
		**Total Indirect Costs**	

Cognizant Federal Agency
(Agency Name, POC Name, and POC Phone Number)

I. Total Direct and Indirect Costs	Funds Requested ($)*
**Total Direct and Indirect Institutional Costs (G + H)**	**317,825.00**

J. Fee	Funds Requested ($)*

K. Total Costs and Fee	Funds Requested ($)*
	**317,825.00**

L. Budget Justification* File Name: VA_Budget_Justification.pdf
(Only attach one file.)

RESEARCH & RELATED Budget {F-K} (Funds Requested)

SUMMARY BUDGET WORKSHEET - up to 5 Sites ver 6.30.17

Expense Category	Budget Period 1	Budget Period 2	Budget Period 3	Budget Period 4	Budget Period 5		
**Primary Site:**	VA Boston Healthcare System						
**VA PI ONLY** (Section A, top line)						Yr 1 Effort (Cal Mo)	Degree
PI Salary	30,222	31,707	32,341	32,988	0	3.0	PhD, MPP
PI Fringe	12,146	12,743	12,998	13,258	0		
**Other VA Personnel** (include Sr/Key VA personnel, exclude IPAs)						Total Yr 1 Effort (Cal Mo)	# unique staff
Hired - Salary	111,492	115,575	119,398	123,715	0	15.6	5
Hired - Fringe	38,302	39,770	41,019	42,523	0		
TBH - Salary	49,122	51,774	55,155	56,216	0	12.0	1
TBH - Fringe	14,737	15,532	16,547	16,865	0		
**Total Other VA Personnel Salary**	160,614	167,350	174,553	179,931	0	27.6	6
	53,038	55,303	57,566	59,388	0		
**Total Personnel** (Total Salary, Wages and Fringe Benefits A+B)	256,020	267,103	277,458	285,565	0		
**Equipment/Start-up** (total-do not itemize)	0	0	0	0	0		
**Travel**	0	0	0	0	0		
**Other Direct Costs** (List any subcategory over $5000, lump remainder)						Total Yr 1 Effort (Cal Mo)	# unique persons
IPA: Katie Colborn	0	21,424	21,424	21,424	0	0.0	1
Supplies	300	300	300	300	0		
Transcription	0	5,880	0	0	0		
	0	0	0				
	0	0	0				
	0	0	0				
**Subtotal Other Direct**	300	27,604	21,724	21,724	0		
**Subtotal Other Sites**	9,644	10,127	10,329	10,536	0		
**Subtotal Non-Personnel** (Equipment, Travel, Other Direct, Other Sites) **(Section F, Line 8)**	9,944	37,731	32,053	32,260	0		
**Total Project**	**265,964**	**304,834**	**309,511**	**317,825**	**0**	**1,198,134**	

Site #2:	Bedford VA						
**Additional VA Personnel**						Total Yr 1 Effort (Cal Mo)	# unique staff
Site PI - Salary	6,938	7,285	7,431	7,580	0	0.6	1
Site PI - Fringe	2,706	2,841	2,898	2,956	0		
Hired - Salary	0	0	0	0	0		
Hired - Fringe	0	0	0	0	0		
TBH - Salary	0	0	0	0	0		
TBH - Fringe	0	0	0	0	0		
**Additional Personnel Total**	9,644	10,127	10,329	10,536	0		
**Equipment/Start-up** (total-do not itemize)	0	0	0	0	0		
**Travel**	0	0	0	0	0		
**Other Direct Costs** (List subcategories over $5000, lump remainder)							
add...		0	0	0	0		
add...		0	0	0	0		
add...		0	0	0	0		
add...		0	0	0	0		
add...		0	0	0	0		
Other direct costs		0	0	0	0		
**Subtotal Other Direct**	0	0	0	0	0		
**Site #2 Totals**	**9,644**	**10,127**	**10,329**	**10,536**	**0**		

Site #3:	Palo Alto, CA						
**Additional VA Personnel**						Total Yr 1 Effort (Cal Mo)	# unique staff
Site PI - Salary	0	0	0	0	0	0.6	1
Site PI - Fringe	0	0	0	0	0		
Hired - Salary		0	0	0	0		
Hired - Fringe		0	0	0	0		
TBH - Salary		0	0	0	0		
TBH - Fringe		0	0	0	0		
**Additional Personnel Total**	0	0	0	0	0		
**Equipment/Start-up** (total-do not itemize)	0	0	0	0	0		
**Travel**	0	0	0	0	0		
**Other Direct Costs** (List subcategories over $5000, lump remainder)							
add...		0	0	0	0		
add...		0	0	0	0		
add...		0	0	0	0		
add...		0	0	0	0		
add...		0	0	0	0		
Other direct costs		0	0	0	0		
**Subtotal Other Direct**	0	0	0	0	0		
**Site #3 Totals**	**0**	**0**	**0**	**0**	**0**		

Budget Justification

Primary Site: VA Boston Healthcare System, Boston, MA

Personnel

Hillary Mull, Ph.D., M.P.P., Multiple Principal Investigator (Corresponding PI), (3.0 cal mos, GS 13/6, 8/8th VA, salary and fringe in year 1: $30,222/ $12,146, salary and fringe in year 2 reflecting step increases: $31,707 /$12,743, salary and fringe in year 3: $32,341/ $12,998, and salary and fringe in year 4: $32,998/$13,258.) Dr. Mull is an Investigator at CHOIR and has worked with Dr. Branch-Elliman, multiple-PI, for more than 5 years. In collaboration with Dr. Branch-Elliman, Dr. Mull will oversee all aspects of the project including coordinating with all study personnel (see below). She will provide oversight of all aspects of data collection and analysis and will manage dissemination of study results and will ensure database development and study analyses are on track. Dr. Mull will lead biweekly project meetings with the entire team and participate in conversations with individual Co-Investigators and research staff as needed. Dr. Mull has established relationships with all members of the project team and has experience in leading successful research projects as well as effectively disseminating findings to operational partners and the broader research community.

Westyn Branch-Elliman, M.D., M.MSc., Multiple Principal Investigator, (3.0 cal mos, GS 15/3, 8/8th VA, donated.) Dr. Branch-Elliman is also an Investigator at the Center for Healthcare Organization and Implementation Research (CHOIR), an HSR&D Center for Innovation (COIN) based at the VA Boston Healthcare System. She is an infectious disease physician at VA Boston Healthcare System and has significant experience using electronic medical record data algorithms to detect healthcare associated infections and an established track record in antimicrobial stewardship research. She has also completed implementation science training through a National Heart Lung and Blood Institute (NHLBI) K12 and the NIH Training Institute for Dissemination and Implementation Research in Health. Her specific role as multiple-PI will be to provide oversight into guideline-recommended practices, to correlate clinical guideline-based recommendations with data extracted by the mining tool, and to train the research assistant in manual review of charts to ensure accuracy of data as well as to provide a gold-standard clinical review. She will also assist with oversight of the sustainability and implementation aspects of the proposal and provide insights from the point of view of a practicing clinician.

Marlena Shin, J.D, M.P.H Co-Investigator, (2.4 cal mos, GS 13/6, 8/8th VA, salary and fringe in year 1: $24,177/ $9,625, salary and fringe in year 2 reflecting step increases: $25,365 /$10,098, salary and fringe in year 3: $25,873/ $10,300, and salary and fringe in year 4: $26391/$10,506.) Ms. Shin is a CHOIR core Investigator. She has led the qualitative research component of multiple studies within the VA and has extensive experience developing qualitative interview guides, conducting interviews, engaging stakeholders, and analyzing qualitative data. Her role in this study will be to develop interview guides, recruit participants, conduct the interviews, analyze the qualitative data, and map findings to implementation strategies to develop the implementation playbook.

Ryann Engle, M.P.H Co-Investigator, (2.4 cal mos, GS 13/3, 8/8th VA, salary and fringe in year 1: $22,105/ $7,361, salary and fringe in year 2 reflecting step increase: $23,252 /$7,743, salary and fringe in year 3: $23,717/ $7,898, and salary and fringe in year 4 reflecting step increase: $25,658/$8,544.) Ms. Engle is a CHOIR Research Health Scientist with extensive experience in qualitative data collection, management, analysis, reporting and dissemination with Co-Investigator experience. Ms. Engle will work with Ms. Shin to refine interview processes, recruit participants for interviews and conduct interviews. For this study, she will assist with qualitative data collection and analysis and serve as a second coder to ensure the reproducibility and reliability of results.

Rebecca Lamkin, M.A., Project Manager, (1.8 cal mos, GS 12/4, 6.4/8ths VA, salary and fringe in year 1: $14,378/ $6,066, salary and fringe in year 2, reflecting step increases: $15,110/ $6,375, salary and fringe in year 3: $15,412/ $6,502, and salary and fringe in year 4 , reflecting step increases, $16,183/ $6,827) Ms. Lamkin is a CHOIR project manager. She will coordinate with project staff across the 3 sites, maintain IRB approval and regulatory documents and will oversee the maintenance of study data in a secure fashion.

Daniel Sturgeon, MS, Programmer (6 cal mos, GS 12/5, 8/8ths VA, salary and fringe estimated in year 1: $50,831/$15,249, salary and fringe in year 2: $51,848/$15,554, salary and fringe in year 3, reflecting step

increases: $54,395/ $16,319, and salary and fringe in year 4, $55,484/ $16,645) Mr. Sturgeon is a highly skilled SAS programmer with sufficient training and experience to complete the study objectives described in the research plan. He will build and maintain data files, aggregate and merge data from the VA Corporate Data Warehouse (CDW), and program electronic algorithms. In collaboration with Drs. Branch-Elliman and Mull, the Mr. Sturgeon will conduct quality assurances tests on the study datasets and assist in downloading CDW data to refine and apply algorithms in years 1-2 of the study and merge files to create datasets for analyses and hypothesis tests in conjunction with the study statistician, Dr. Colborn. Mr. Sturgeon will contribute to the creation of presentations, reports, and manuscripts, and respond to requests from Co-Investigators.

TBD Research Assistant (12 cal mos, GS 7/1, 8/8ths VA, salary and fringe in year 1: $49,122/ $14,736, salary and fringe in year 2, reflecting step increases: $51,744/ $15,532, salary and fringe in year 3, reflecting step increases: $55,155/ $16,547, salary and fringe in year 4 reflecting step increases: $56,216/ $16,865). We will hire a CHOIR Research Assistant who will be responsible for chart review data collection and management. They will also assist with qualitative work both in interviewing subjects and coding transcribed interviews. The RA will attend all project meetings and keep study records. They will also maintain regular contact with members of the Operational Steering Committee and coordinate meetings and reports.

Equipment ($0): No equipment over $5,000 is required for this project.

Travel ($0): No travel funds are being requested for this project.

Materials and Supplies ($1200): We are requesting $300 annually for supplies to complete the project work. These include academic texts guiding research analyses, medical texts documenting current coding practices, and computer programming manuals.

Alpha Transcription ($5,880): This expense includes the cost of Alpha Transcription to transcribe 60 qualitative interviews ($98 per interview) in year 2 of the study. Other groups at CHOIR have used them in the past as they are substantially less expensive than VA-approved alternatives (e.g., Susan Zickmund/CTSP).

Publications ($0). Many of the well-respected peer-reviewed journals in the field, including *BMC Health Services Research*, require publication fees from authors to make open access available for their articles. When necessary for publication, we request reimbursement of publication fees not to exceed $3000.

IPA ($64,272)
Kathryn Colborn, Ph.D., Statistician, (VA WOC, 1.2 cal mos Y2-4). Dr. Colborn is a statistician at the University of Colorado with extensive experience developing advanced clinical informatics tools for quality measurement, including measurement of healthcare-associated infections and surgical site infections. She will contribute to all data modeling and statistical analyses work on the project and oversee the statistical analysis of quantitative data collected during the study.

Site 2: Edith Norse Rogers VA Hospital, Bedford, MA

A. Rani Elwy. Ph.D., Co-Investigator, (0.6 cal mos, GS 14/5, 8/8ths VA, salary and fringe in year 1: $6,938/ $2,706, salary and fringe in year 2, reflecting step increases: $7,285/ $2,841 salary and fringe in year 3,: $7,431/ $2,898, salary and fringe in year 4: $7,580/ $2,956) is a core investigator at CHOIR, and the Director of the Implementation Science Core in the Department of Psychiatry and Human Behavior at Brown University's Alpert Medical School. Dr. Elwy's role on this project will be to provide insight into the implementation and qualitative aspects of the proposal. This will involve providing oversight of the development of the interview guide based on the Dynamic Sustainability Framework, mapping facilitators and barriers to implementation strategies, and developing the implementation playbook. She will participate in all meetings and assist with the writing of manuscripts for publication.

Site 3: VA Palo Alto Health Care System, Palo Alto, CA

Mary Hawn, M.D., M.P.H., Co-Investigator (0.6 cal mos, 8/8th VA, GS 15-3, donated time) is a surgeon at the VA Palo Alto Health Care System, investigator at the HSR&D Center for Innovation to Implementation (Ci2i)

COIN, and Stanford Medicine Professor of Surgery and Chair of the Department of Surgery at Stanford University. Dr. Hawn has significant health services research experience in surgical quality and the Surgical Care Improvement Project along with expertise as a colorectal surgeon. Her role will be to provide insights from the point of view of a surgeon, and to help put findings into a pragmatic context. Dr. Hawn will give input into the implementation playbook. She will participate in all meetings and assist with the writing of manuscripts for publication.

RESEARCH & RELATED BUDGET - Cumulative Budget

	Totals ($)	
Section A, Senior/Key Person		178,403.00
Section B, Other Personnel		907,743.00
Total Number Other Personnel	24	
Total Salary, Wages and Fringe Benefits (A+B)		1,086,146.00
Section C, Equipment		0.00
Section D, Travel		0.00
1. Domestic	0.00	
2. Foreign	0.00	
Section E, Participant/Trainee Support Costs		0.00
1. Tuition/Fees/Health Insurance	0.00	
2. Stipends	0.00	
3. Travel	0.00	
4. Subsistence	0.00	
5. Other	0.00	
6. Number of Participants/Trainees	0	
Section F, Other Direct Costs		111,988.00
1. Materials and Supplies	0.00	
2. Publication Costs	0.00	
3. Consultant Services	0.00	
4. ADP/Computer Services	0.00	
5. Subawards/Consortium/Contractual Costs	0.00	
6. Equipment or Facility Rental/User Fees	0.00	
7. Alterations and Renovations	0.00	
8. Other 1	111,988.00	
9. Other 2	0.00	
10. Other 3	0.00	
Section G, Direct Costs (A thru F)		1,198,134.00
Section H, Indirect Costs		0.00
Section I, Total Direct and Indirect Costs (G + H)		1,198,134.00
Section J, Fee		0.00
Section K, Total Costs and Fee (I + J)		1,198,134.00
